# Identification of an R1-type pyocin previously misannotated as a prophage in Pseudomonas aeruginosa ATCC 27853

**DOI:** 10.1099/mic.0.001692

**Published:** 2026-04-13

**Authors:** Rayhaan Gerard Pais, Mathias Müsken, Belinda Loh

**Affiliations:** 1 Department of Infection Research and Diagnostics, Fraunhofer Institute for Cell Therapy & Immunology (IZI), Perlickstr. 1, Leipzig, 04103, Germany; 2 Central Facility for Microscopy, Helmholtz Centre for Infection Research, Inhoffenstr. 7, 38124 Braunschweig, Germany

**Keywords:** antimicrobials, prophage, *Pseudomonas aeruginosa* ATCC 27853, pyocin, tailocin

## Abstract

While *Pseudomonas aeruginosa* ATCC 27853 is a widely used reference strain with previously characterized prophage regions, our use of one of the latest prophage prediction tools, PHASTEST, helped reveal a critical misclassification in its genome. Using this tool, we initially identified six prophage regions, with four classified as intact; however, in-depth analysis demonstrated that one of these predicted intact prophages was, in fact, a functional pyocin-encoding region. Specifically, the region spanning 679,586–698,056 bp, initially annotated as an intact prophage, was definitively re-identified as a region harbouring an R1-type pyocin. The most recent literature regarding prophages in *P. aeruginosa* ATCC 27853 classifies the region spanning 683,173–696,044 bp as a prophage. This region falls entirely within the genomic region we describe and reclassify, further emphasizing the importance of the reclassification performed in this study. The identified R1-type pyocin was induced using mitomycin C, processed via tangential flow filtration, and its bactericidal activity was confirmed against a clinical *P. aeruginosa* isolate via spot-test killing assays and absorbance-based assays. Transmission electron microscopy revealed R-type pyocin particles averaging 133 nm in length. This misidentification of a pyocin as a prophage critically underscores the inherent limitations of current bioinformatic tools in accurately distinguishing between these distinct phage-derived elements, thereby highlighting the urgent need for more refined annotation methodologies. Accurate identification of such elements is essential, as they may influence experimental outcomes and provide new insights into bacterial defence mechanisms.

## Data Availability

The sequence of *Pseudomonas aeruginosa* ATCC 27853/DSM 1117 is available on the National Center for Biotechnology Information (NCBI Reference Sequence: NZ_CP101912.1). The R1-type pyocin-encoding gene clusters mentioned as part of the blastn search (Results, Sequence Analysis Confirms R1-Type Pyocin Identity) can be found on GenBank under the following accession numbers: ‘S14’ – OQ870459.1, ‘S19’ – OQ870464.1 and ‘S20’ – OQ870465.1 from the 2023 article by Saha *et al*. 1] confirming their identity as R1-type pyocins.

The tail-collar fibre protein from the pyocin-encoding gene cluster of *P. aeruginosa* ATCC 27853 and the tail fibre protein from the pyocin-encoding gene cluster ‘S14’ can be found under the GenBank accession numbers WP_003161927.1 and WHT11591.1, respectively.

## Introduction

Bacteriophages and phage-derived elements play a major role in bacterial evolution and ecology. Temperate bacteriophages can integrate their genomes into the chromosomes of their bacterial host, where they persist as prophages until conditions favour their reactivation [2]. Prophages can influence host biology in diverse ways, conferring both benefits and disadvantages [3]. Among the most consequential benefits is the enhancement of bacterial virulence, which contributes substantially to microbial diversity [2].

Among phage-derived elements are pyocins, which are phage tail-like bacteriocins produced by *Pseudomonas* spp., capable of killing closely related bacterial strains. Pyocins are broadly classified into three major types: the phage tail-like R-type and F-type pyocins, and soluble S-type pyocins. R-type and F-type pyocins are high-molecular weight, protease-resistant particles that resemble bacteriophage tails but, unlike bacteriophages, cannot replicate; R-type pyocins resemble the rigid, contractile tails of myovirus-like phages, whereas F-type pyocins are non-contractile and flexible, resembling the tails of siphovirus-like phages [4,5]. Both R-type and F-type pyocins bring about cell death by depolarizing the target bacterial cell membrane [4,6]. The mechanism of killing action for R-type pyocins has been previously described [7]; however, the killing mechanism of F-type pyocins is still not well understood [4]. S-type pyocins are soluble, protease-sensitive bacteriocins, which are similar to the colicins of *Escherichia coli* and bring about bacterial cell death via their endonuclease or pore-forming ability [5]. Among the different types of pyocins, R-type pyocins are of particular interest as alternative antimicrobial therapies due to their potent antibacterial activity against closely related strains, known receptors and well-characterized mechanism of action [8]. R-type pyocins are further classified into five distinct subtypes (R1–R5) based on variations in the tail fibre protein, which is responsible for receptor specificity [7].

As pyocins mediate selective killing of closely related bacterial strains, they shape the microbial community structure and provide competitive advantages in interbacterial interactions [9]. The structural and evolutionary similarity between pyocins and temperate phages can lead to misclassification in genomic annotations, as current prophage-prediction tools often rely on phage homology without distinguishing functional differences.

*Pseudomonas aeruginosa* ATCC 27853 is a well-established reference strain widely used in studies of biofilms, antibiotic susceptibility and metabolic activity [10]. Despite its extensive characterization, we found that a prophage-prediction tool, as well as the most recent prophage-focused literature on this strain [10], had misidentified a functional R1-type pyocin gene cluster as a prophage. Here, we identify and classify the pyocin type and perform induction, tangential flow filtration (TFF) and basic characterization to confirm its activity against a clinical *P. aeruginosa* isolate.

This article highlights a key limitation of current computational approaches and underscores the need for more accurate discrimination between prophages and pyocins, or more broadly, tailocins. Accurate discrimination is essential for precise genome annotation, avoiding unintended experimental artefacts in *Pseudomonas* research and uncovering overlooked antibacterial systems that may inform the development of new antimicrobial strategies.

## Methods

### Bacterial strains

*P. aeruginosa* ATCC 27853/DSM 1117 was acquired from the DSMZ-German Collection of Microorganisms and Cell Cultures. The clinical *P. aeruginosa* isolate, *P. aeruginosa* 008, was acquired from the University Hospital Leipzig.

### Prophage prediction and manual curation of pyocin-associated regions

The complete genome sequence of *P. aeruginosa* ATCC 27853 (NZ_CP101912.1) was uploaded to the PHASTEST Version 3.0 webserver (PHAge Search Tool with Enhanced Sequence Translation) [11] and analysed using both lite and deep annotation modes.

All predicted prophage regions were subsequently inspected manually. The predefined criteria used to differentiate putative prophage sequences from pyocin-encoding gene clusters included: (i) the presence of the flanking *trpE–trpG* gene pair, a known hotspot for pyocin integration in *P. aeruginosa* [12]; (ii) the presence of the regulatory genes *prtN* and *prtR* [12]; and (iii) the absence of hallmark phage structural or replication genes, such as capsid, terminase or integrase genes [13].

### Bioinformatic determination of pyocin type

The putative pyocin-encoding gene cluster in *P. aeruginosa* ATCC 27853 (genomic coordinates: 679,269–696,399) was analysed using blastn [14] against standard databases with a maximum of 500 target sequences, followed by sorting the results in ascending order of accession length. Within this cluster, the amino acid sequence of the phage tail-collar fibre protein (WP_003161927.1; genomic region 685,629–687,734) was extracted and subjected to a global pairwise alignment via EMBOSS STRETCHER [15] against the tail fibre protein sequence of the most similar pyocin-encoding region identified in the blastn search [4].

### Induction and TFF of the R1-type pyocin

A single colony of *P. aeruginosa* ATCC 27853 was grown overnight in 5 ml of Lysogeny Broth (LB) at 37 °C, 220 r.p.m. The culture was diluted 1:20 (v/v) into 100 ml of LB in a 500 ml Erlenmeyer flask and grown at 37 °C, 220 r.p.m. until OD_600_≈0.60 (OD_600_ refers to absorbance measured at 600 nm). Pyocin production was induced by adding 3 µg ml^−1^ of mitomycin C, followed by incubation at 37 °C, 220 r.p.m. for ~18 h. Cultures were centrifuged at 8,000 ***g*** for 30 min at 4 °C, and supernatants were filtered through a 0.22 µm filter and stored at 4 °C. An additional control sample was also prepared in the same manner, except that, after mitomycin C induction and incubation at 37 °C, 220 r.p.m. for ~18 h, 10 µl of chloroform was added to the sample to lyse any remaining cells.

Uninduced controls were prepared identically, omitting the addition of mitomycin C. A subset of uninduced cultures was lysed by either 10 µl of chloroform treatment or four freeze–thaw cycles (−20 to 25 °C) prior to centrifugation and filtration.

Removal of small molecules (<100 kDa) and buffer exchange of R1-type pyocin from LB to storage buffer (10 mM Tris-HCl, pH 7.0; 10 mM MgSO_4_) (HCl = hydrochloric acid, MgSO_4_ = magnesium sulphate) were performed using TFF (µPulse TFF system) with a 100 kDa molecular weight cut-off (MWCO) polyethersulfone (PES) filter, according to the manufacturers’ instructions. Twenty millilitres of pyocin-containing supernatant was exchanged into 20 ml of storage buffer. Retentates containing R1-type pyocins were stored at 4 °C until use. Uninduced control samples underwent TFF in the same manner.

### Soft-agar overlay assay

A single colony of the *P. aeruginosa* 008 clinical isolate was grown in LB at 37 °C with shaking at 220 r.p.m., to an OD_600_ of 0.60. Soft-agar overlays were performed by mixing the culture 1:10 (v/v) with 0.7% (w/v) LB soft agar, vortexed and poured over an LB agar plate. After solidification, 2 µl of serially diluted pyocin preparations (10^0^ to 10^−4^ in LB) was spotted onto the bacterial lawn and allowed to dry. Plates were incubated at 37 °C for 16 h before zones of growth inhibition were recorded. All assays were performed in biological triplicates.

### Verification of the lack of amplification potential

A single colony of *P. aeruginosa* 008 was inoculated into LB and cultured at 37 °C with shaking at 220 r.p.m. to an OD_600_ of 0.60. TFF-processed R1-type pyocin in storage buffer was added at a 1:100 (v/v) ratio, and the culture was incubated under the same conditions for 16 h. Cells were removed by centrifugation at 8,000 ***g*** for 10 min at 4 °C. The supernatant was treated with chloroform to a final concentration of 0.05% (v/v), centrifuged again under the same conditions, and the clarified supernatant was collected. Samples were serially diluted (10^0^ to 10^−3^) in LB, and 2 µl of each dilution was spotted onto *P. aeruginosa* 008 lawns prepared as described in the ‘Soft-agar overlay assay’ section.

To determine pyocin activity prior to the amplification assay (no-incubation control), the TFF-processed R1-type pyocin was diluted 1:100 (v/v) in LB and immediately serially diluted (10^0^ to 10^−3^) in LB. Aliquots (2 µl) of each dilution were spotted onto *P. aeruginosa* 008 lawns and incubated at 37 °C for 16 h. Pyocin activity before and after the amplification assay was compared. All assays were performed in biological triplicates.

To assess whether the incubation and processing steps involved in the amplification assay itself did not adversely affect pyocin activity, TFF-processed R1-type pyocin in storage buffer was added to LB, in the absence of *P. aeruginosa* 008, at a 1:100 (v/v) ratio and subjected to identical incubation (16 h, 37 °C, 220 r.p.m.), chloroform treatment and centrifugation as described above. Clarified supernatants were serially diluted and spotted onto *P. aeruginosa* 008 lawns as described above.

As a positive control, the lytic bacteriophage P0080_Pa from our laboratory was added at a 1:100 (v/v) ratio to a log-phase culture of *P. aeruginosa* 008 (OD_600_=0.60) to achieve a final titre of 2.5×10^4^ plaque-forming units (PFU) per millilitre. The culture was then processed as described above. Collected supernatants were serially diluted (10^0^ to 10^−7^) in LB, and 2 µl of each dilution was spotted onto a *P. aeruginosa* 008 lawn. Plates were incubated at 37 °C for 16 h. Phage activity before and after the amplification assay was evaluated.

### Transmission electron microscopy

Electron microscopy sample preparation was performed as previously described [16]. In brief, pyocin samples were absorbed onto thin carbon films, washed, negatively stained with 2% (w/v) aqueous uranyl acetate solution (pH 5.0) and examined with an EM 910 transmission electron microscope (Carl Zeiss, Oberkochen, Germany) at an accelerating voltage of 80 kV. Size determination of particles was performed using ITEM Software (Olympus Soft Imaging Solutions, Münster).

### Absorbance-based growth inhibition assays

*P. aeruginosa* 008 was grown overnight in LB at 37 °C, 220 r.p.m. Ten-times LB was diluted to 1× in TFF-processed R1-type pyocin in storage buffer (10 mM Tris-HCl, pH 7.0; 10 mM MgSO_4_), after which the overnight culture was added at a 1:33 (v/v) ratio. Absorbance (OD_600_) was recorded every 60 min for 6 h (Fisherbrand Cell Density Meter model 40). For proteinase K experiments, prior to performing the absorbance-based assay, the enzyme was added to TFF-processed R1-type pyocin in storage buffer to a final concentration of 0.2 mg ml^−1^ and incubated at 37 °C for 1 h. For self-susceptibility experiments, *P. aeruginosa* ATCC 27853 was used instead of *P. aeruginosa* 008.

Control assays were performed using TFF-processed uninduced lysates (chloroform-lysed or freeze–thaw-lysed cultures), substituted in place of pyocin in storage buffer. An additional growth control consisted of *P. aeruginosa* 008 (or ATCC 27853) cultured in 10× LB diluted to 1× in storage buffer to assess the growth of the strain in the absence of potential growth-inhibiting agents.

All assays were performed in biological triplicates. The data from the absorbance-based assays were analysed and represented as line graphs along with the mean and sd using GraphPad Prism 10.

## Results

### Reclassification of a prophage-encoding region as a pyocin-encoding gene cluster in *P. aeruginosa* ATCC 27853

**Fig. 1.**
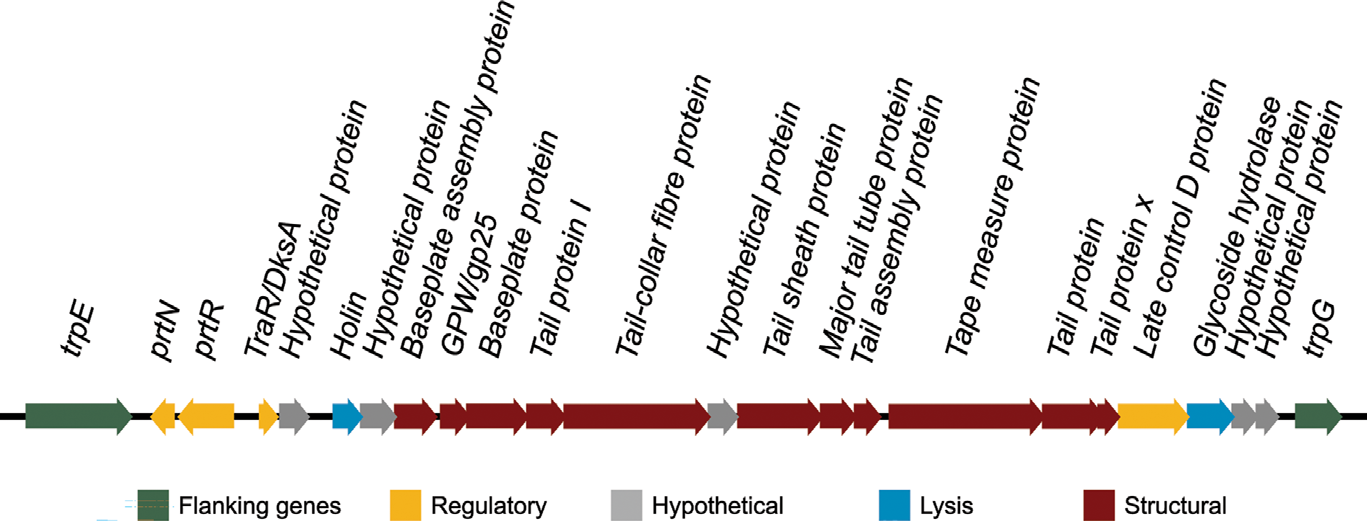
Schematic representation of the pyocin-encoding gene cluster (nucleotide range: 679,269–696,399) situated between the *trpE* and *trpG* genes in *P. aeruginosa* ATCC 27853. Arrowheads point in the direction of transcription.

As part of an analysis to characterize the genomic landscape of the reference strain *P. aeruginosa* ATCC 27853, we performed a screen for mobile genetic elements and prophages. One of the latest prophage analysis tools, PHASTEST, identified six prophage regions, with four predicted as ‘intact’ (Supplementary Information 1, available in the online Supplementary Material). Manual curation revealed that one predicted ‘intact’ region (region 1; genomic coordinates 679,586–698,056) lacked canonical prophage features such as capsid, terminase and integrase genes [13]. Instead, the *trpE* gene (677,790–679,268) was found upstream of region 1, and the *trpG* gene (696,400–697,005) at the end of region 1; the *trpE*/*trpG* flanking gene pair is a known hotspot for pyocin integration in *P. aeruginosa* [12]. The region also contained the regulatory genes *prtN* and *prtR* [12]. These characteristics are consistent with a pyocin-encoding biosynthetic gene cluster rather than a prophage (Fig. 1). This reclassification rectifies not only a misidentification by PHASTEST but also a prior misidentification in the literature, which was performed using the prophage prediction tools Prophinder [17] and PHAST [18], that labelled a similar genomic region (683,173–696,044 bp) as a prophage [10].

The remaining prophage regions (regions 2–6) in the genome of *P. aeruginosa* ATCC 27853 predicted by PHASTEST did not fulfil the criteria consistent with a *P. aeruginosa* pyocin-encoding biosynthetic gene cluster. For prophage regions 2–6, none of the regions (or regions upstream and downstream) contained the flanking gene pair *trpE–trpG* or the regulatory genes *prtN* and *prtR*. In addition, prophage regions 2–6 contained at least one gene related to prophage features, such as capsid, terminase and integrase genes (Supplementary Information 1).

### Sequence analysis confirms R1-type pyocin identity

The structural components of this biosynthetic gene cluster were analysed to confirm its pyocin identity. blastn analysis of the putative pyocin cluster showed the highest similarity to R1-type pyocin gene clusters, namely S14 (99.37% identity, 100% query coverage), S19 (99.36% identity, 100% query coverage) and S20 (99.36% identity, 100% query coverage) (Fig. S1) [1]. Global pairwise alignment of the tail-collar fibre protein (WP_003161927.1) from the pyocin-encoding gene cluster of *P. aeruginosa* ATCC 27853 with the corresponding tail fibre protein (WHT11591.1) from the closest homologue, the ‘S14’ cluster, showed that both amino acid sequences were 100% identical to each other (Supplementary Information 2). These results unambiguously assign the encoded bacteriocin as an R1-type pyocin [4].

### Induction, activity, lack of amplification potential and structural confirmation of the R1-type pyocin

**Fig. 2.**
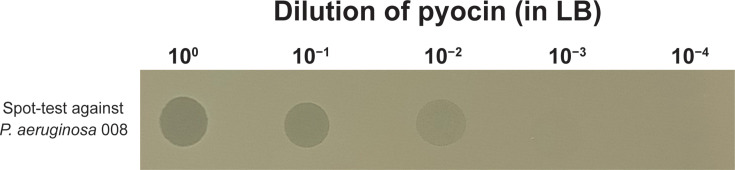
Qualitative serial dilution spot-test assay showing killing of the clinical isolate *P. aeruginosa* 008, suggesting preliminary evidence of activity potentially mediated by a non-replicative toxin, i.e. a pyocin, rather than a replicative phage.

Although bioinformatic analysis identified a putative functional R1-type pyocin gene cluster, experimental validation was necessary to confirm its inducibility and production. Pyocin synthesis was induced in the *P. aeruginosa* ATCC 27853 reference strain using mitomycin C, and bacteriocin activity was evaluated by a spot-test killing assay. Clear zones of inhibition were observed on bacterial lawns for the undiluted, 10-fold- and 100-fold-diluted samples (Fig. 2, raw image in Fig. S2A). Notably, the absence of plaque formation at higher dilutions could suggest preliminary evidence that the zones of inhibition were due to pyocin activity rather than phage replication, supporting the presence of a non-replicative toxin [4,13].

*P. aeruginosa* ATCC 27853 is predicted to harbour multiple prophage regions (regions 2–6 predicted by PHASTEST, Supplementary Information 1). To verify that the activity of the active antibacterial agent was not attributable to a replicative agent, such as an induced prophage, but instead to a non-replicative agent, i.e. the R1-type pyocin, amplification potential was assessed. To this end, putative TFF-processed R1-type pyocin was incubated with a susceptible host culture (*P. aeruginosa* 008), after which cell-free supernatants were recovered and evaluated for changes in bactericidal activity relative to the pre-incubation sample.

**Fig. 3.**
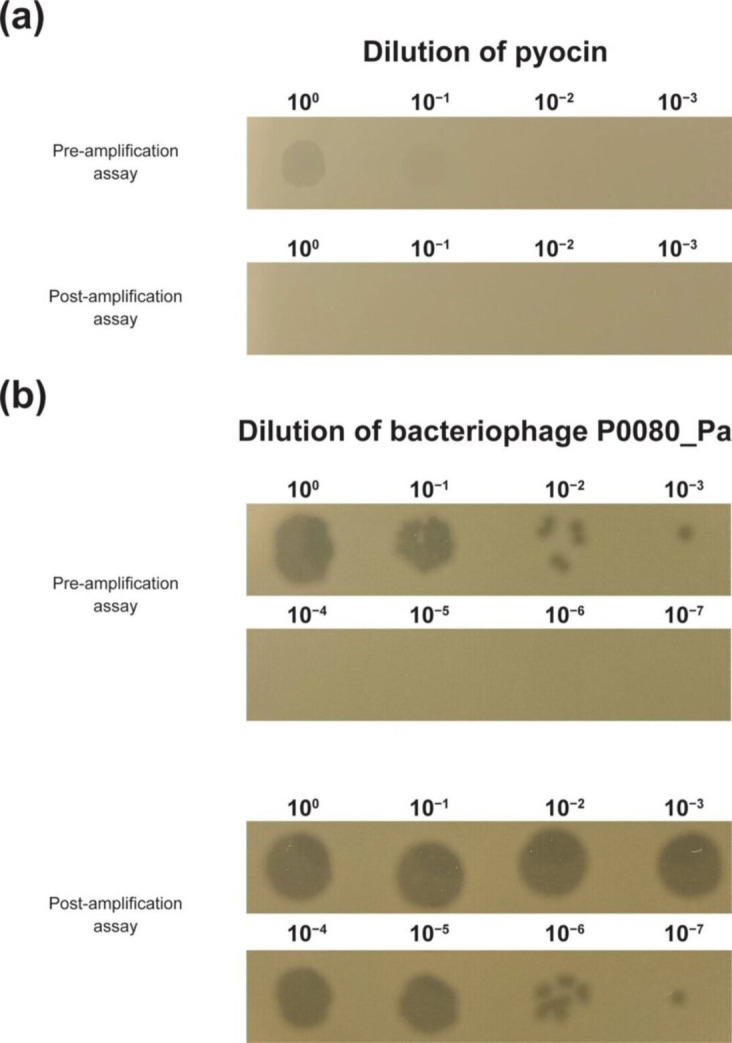
Lack of replicative ability of the active agent in TFF-processed R1-type pyocin samples. (**a**) Serial dilution spot-test assay of TFF-processed R1-type pyocin before and after subjection to the amplification assay, confirming lack of replicative ability. (**b**) Serial dilution spot-test assay of bacteriophage P0080_Pa (assay control) before and after subjection to the amplification assay, confirming that the assay is able to support amplification of a replicative agent.

Supernatants collected following the amplification assay showed no increase in detectable bactericidal activity across serial dilutions compared with TFF-processed R1-type pyocin prior to the assay, indicating that no amplification of the active agent had occurred (Fig. 3a, raw image in Fig. S2B). Notably, supernatants recovered in the post-amplification assay showed no detectable bactericidal activity. As the amplification assay involves incubation of the pyocin with a susceptible host in liquid culture, i.e. *P. aeruginosa* 008, this loss of activity is consistent with the non-replicative, single-use mechanism characteristic of R-type pyocins [19].

In contrast, supernatants obtained after subjecting TFF-processed R1-type pyocin to the amplification assay in the absence of *P. aeruginosa* 008 retained bactericidal activity comparable to that of the pre-amplification sample, confirming that the assay conditions themselves do not inherently compromise R1-type pyocin activity (Fig. S3).

When the control bacteriophage P0080_Pa was subjected to the same amplification assay, an ∼10^4^-fold increase in detectable bactericidal activity was observed across serial dilutions relative to the pre-amplification sample (Fig. 3b, raw image in Fig. S2C). This observation confirms that the assay supports amplification of a replicative agent.

**Fig. 4.**
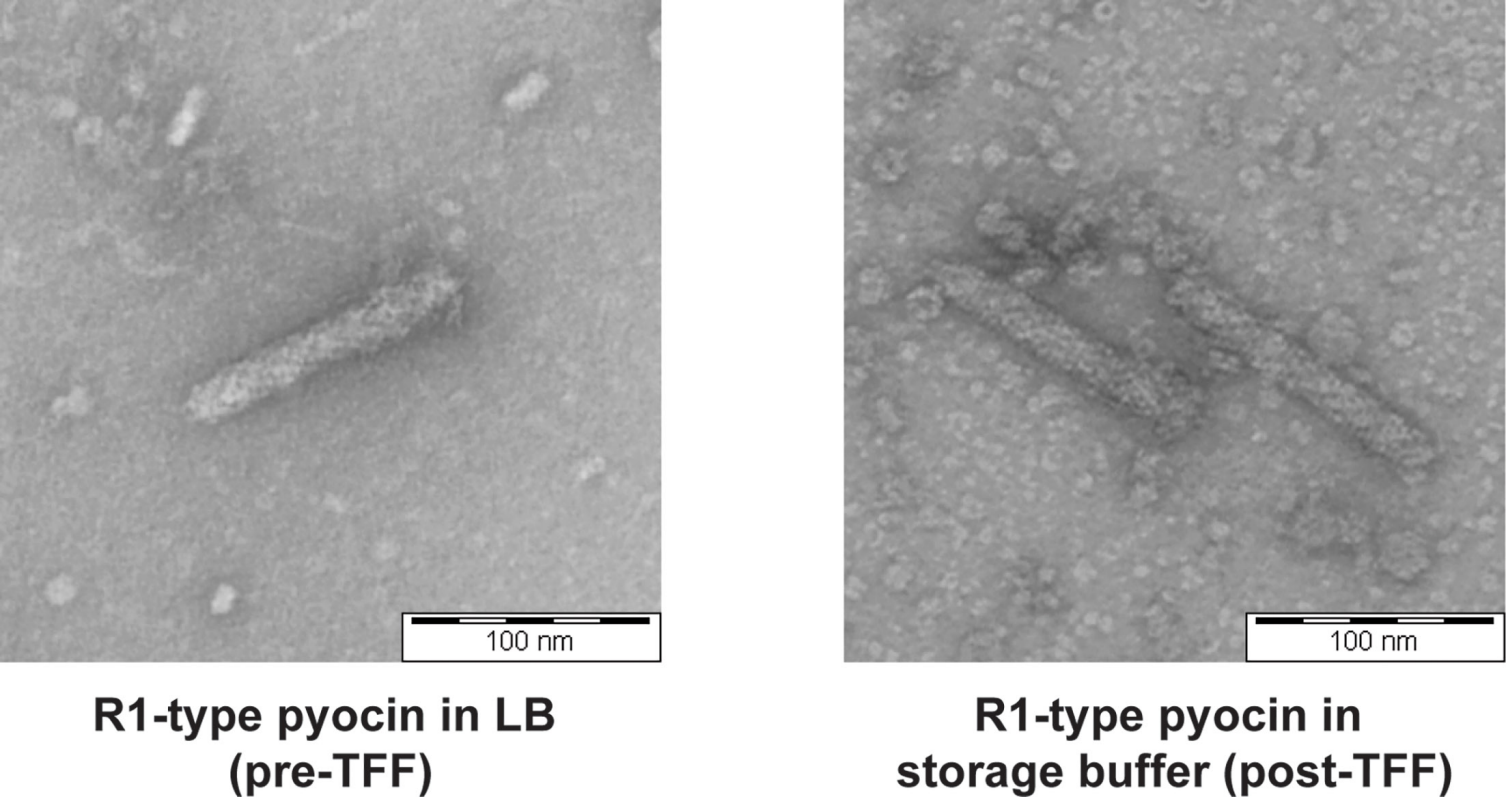
Transmission electron micrographs showing the presence of R-type pyocin particles pre-TFF in LB (left) and post-TFF in storage buffer (right).

R1-type pyocins structurally resemble the contractile tails of myovirus-like bacteriophages [20]. Therefore, to confirm that the functional pyocin is indeed an R1-type pyocin, transmission electron microscopy of the pyocin samples was conducted. Images showed the presence of R-type pyocins (Fig. 4, raw images in Fig. S4A, B) with an average length of 133 nm.

### Validation of R1-type pyocin activity against a clinical *P. aeruginosa* strain

**Fig. 5.**
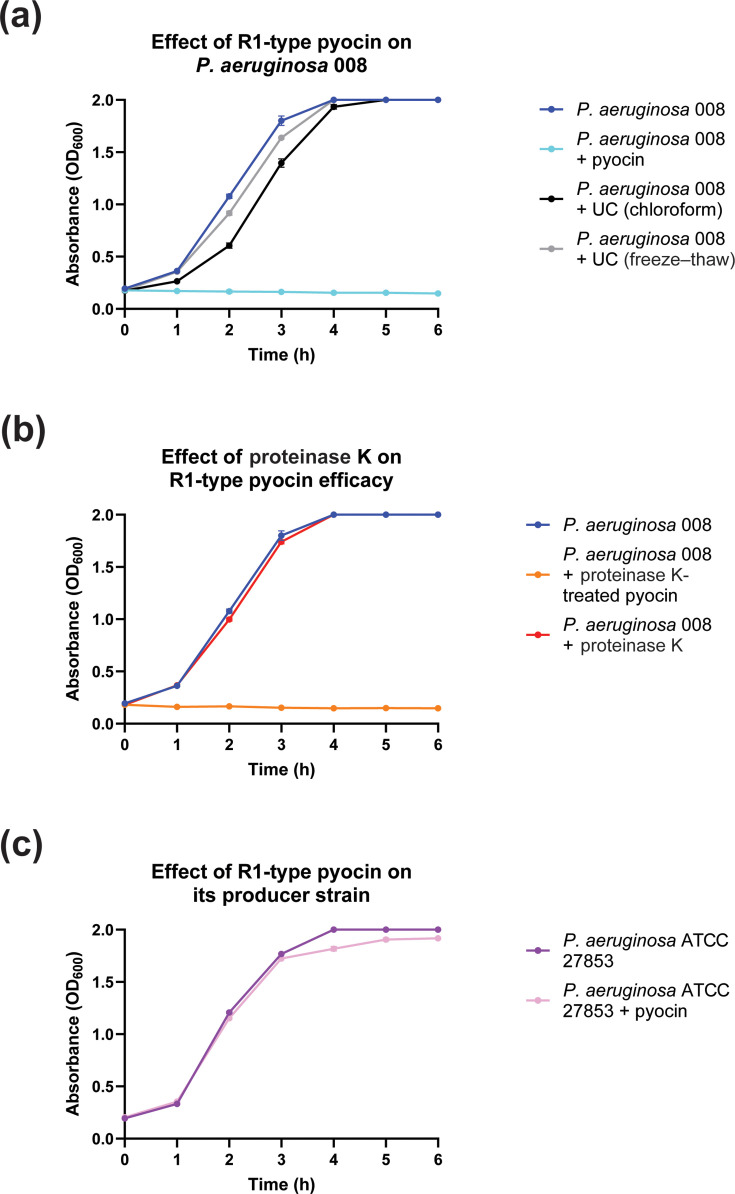
Absorbance-based assays confirming the killing activity of the R1-type pyocin. (**a**) Effect of TFF-processed R1-type pyocin on *P. aeruginosa* 008. (**b**) Effect of proteinase K on the efficacy of the R1-type pyocin. (**c**) Effect of the R1-type pyocin on its producer strain, *P. aeruginosa* ATCC 27853, to evaluate self-susceptibility. All line graphs are represented as mean and sd of biological triplicates (*n*=3) for each condition. UC, uninduced control.

Initial experiments were performed using pyocin-containing lysates, which also contained mitomycin C, other proteins and cell debris that could interfere with and misrepresent activity measurements. Therefore, pyocin preparations were processed using TFF to remove media components and small molecules and to exchange the pyocin into a Tris-based storage buffer. The resulting buffer-exchanged pyocin was then subjected to a series of controlled growth assays to further characterize the R1-type pyocin. TFF-processed R1-type pyocin inhibited the growth of the clinical isolate *P. aeruginosa* 008, whereas uninduced lysates, storage buffer alone or proteinase K alone showed comparatively no inhibitory effect on bacterial growth, confirming that growth suppression was specifically due to the R1-type pyocin (Fig. 5a, b). An additional TFF-processed preparation generated by mitomycin C induction, incubation and subsequent chloroform lysis produced inhibitory activity comparable with the ‘*P. aeruginosa* 008 + pyocin’ condition shown in Figs 5a and S5.

Because S-type pyocins are protease-sensitive, while R-type pyocins are protease-resistant, persistence of growth inhibition after proteinase K treatment confirmed that S-type pyocins were not involved in growth inhibition (Fig. 5b) [1,4].

The response of the pyocin-producing strain, *P. aeruginosa* ATCC 27853, to its own R1-type pyocin was also evaluated. Consistent with the general observation that bacterial cells are resistant to their own R-type pyocin, *P. aeruginosa* ATCC 27853 cells were not killed when treated with their endogenous R1-type pyocin (Fig. 5c) [21]. However, it is crucial to note that the general observation of self-resistance cannot be stated as a strict rule or dogma for all cases, since recent work by Mei *et al*. successfully challenges the general dogma of self-resistance and indicates that self-susceptibility to an endogenous R1-type pyocin can occur in R1-type pyocin-producing strains [8]. The absorbance values recorded for each condition can be found in Supplementary Information 3.

## Discussion

*P. aeruginosa* ATCC 27853 (National Center for Biotechnology Information, NCBI Reference Sequence: NZ_CP101912.1) is a well-established reference strain in biomedical research, adopted as a model to study antibiotic susceptibility, biofilm formation and metabolic activity. Despite its extensive characterization, our results reveal that even well-studied genomes can harbour functional elements that are misannotated.

The reclassification of a previously predicted prophage as an R1-type pyocin gene cluster highlights a key limitation in current computational prophage prediction tools. Tailocins are phage-derived proteinaceous particles that mediate bacterial competition through cell lysis of target strains while sparing the producing population. They represent a form of ‘altruistic suicide’ that contributes to microbial fitness and ecological success.

Our findings underscore that pyocins, and more generally, tailocins, may be underrepresented in genome annotations due to misclassification as prophages. Accurate identification of these elements is critical for understanding bacterial ecology, interstrain competition and experimental outcomes in microbiology research. The identification of the R1-type pyocin in *P. aeruginosa* ATCC 27853 emphasizes the need for improved bioinformatic tools to distinguish tailocins from prophages.

Based on the insights gained in this study, several refinements to current prophage prediction tools could improve annotation accuracy. Integration of additional genomic context features, for example, the presence or absence of genes essential for intact prophages, such as capsid-associated genes, integrases and terminases, could enhance discrimination. Predicted regions lacking such hallmark prophage components could be penalized within scoring algorithms, thereby reducing the ‘completeness’ score of a predicted region. Alternatively, the region can simply be flagged as ‘questionable’, with explicit reporting of the missing features. Incorporating these criteria would prompt closer manual inspection of high-scoring but incomplete regions. A recently released preprint describes a computational approach to identify pyocin-encoding gene clusters [22]. This may complement current prophage-prediction tools when annotating bacterial genomes while allowing the specific identification of pyocins.

Ultimately, this study supports broader exploration of tailocin diversity and function, which may uncover novel antibacterial strategies and deepen our understanding of microbial competition, ecological dynamics and the evolutionary significance of phage-derived bacteriocins.

## Supplementary material

10.1099/mic.0.001692 Uncited Supplementary Material 1.

## Abbreviations

DSMZ: German Collection of Microorganisms and Cell Cultures
LB: Lysogeny Broth
NCBI: National Center for Biotechnology Information
TFF: tangential flow filtration
