# Supplementary material for: Identification of an R1-type pyocin previously misannotated as a prophage in Pseudomonas aeruginosa ATCC 27853
Source: Microbiology (Reading). 2026 Apr 13;172(4):001692. doi: 10.1099/mic.0.001692 (PMC13075995; doi:10.1099/mic.0.001692)
Supplement: Uncited Supplementary Material 1. [file mic-172-01692-s001.pdf]

Supplementary Figure 1

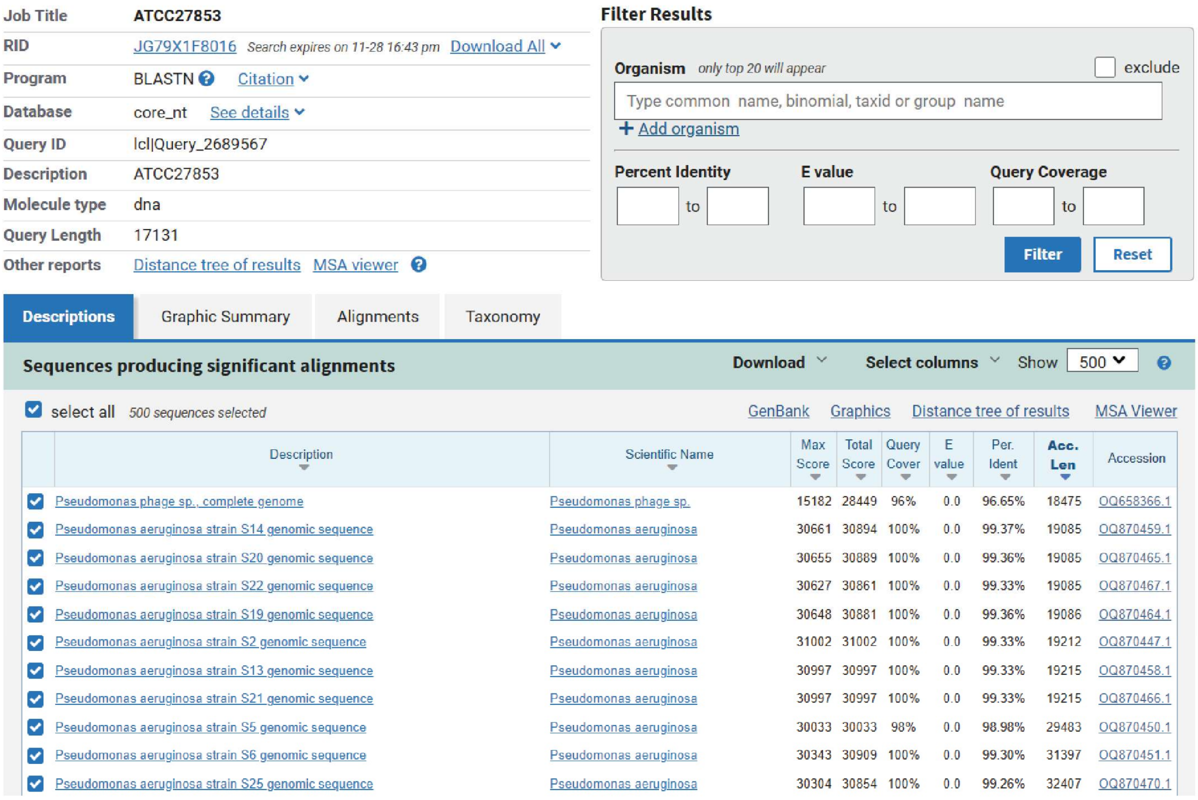

Job Title	ATCC27853
RID	JG79X1F8016 *Search expires on 11-28 16:43 pm* Download All
Program	BLASTN Citation
Database	core_nt See details
Query ID	lcl|Query_2689567
Description	ATCC27853
Molecule type	dna
Query Length	17131
Other reports	Distance tree of results MSA viewer

Filter Results

Organism *only top 20 will appear* exclude

Type common name, binomial, taxid or group name

+ Add organism

Percent Identity to E value to Query Coverage to

Filter Reset

Descriptions Graphic Summary Alignments Taxonomy

Sequences producing significant alignments Download Select columns Show 500

select all *500 sequences selected* GenBank Graphics Distance tree of results MSA Viewer

Description	Scientific Name	Max Score	Total Score	Query Cover	E value	Per. Ident	Acc. Len	Accession
Pseudomonas phage sp., complete genome	Pseudomonas phage sp.	15182	28449	96%	0.0	96.65%	18475	OQ658366.1
Pseudomonas aeruginosa strain S14 genomic sequence	Pseudomonas aeruginosa	30661	30894	100%	0.0	99.37%	19085	OQ870459.1
Pseudomonas aeruginosa strain S20 genomic sequence	Pseudomonas aeruginosa	30655	30889	100%	0.0	99.36%	19085	OQ870465.1
Pseudomonas aeruginosa strain S22 genomic sequence	Pseudomonas aeruginosa	30627	30861	100%	0.0	99.33%	19085	OQ870467.1
Pseudomonas aeruginosa strain S19 genomic sequence	Pseudomonas aeruginosa	30648	30881	100%	0.0	99.36%	19086	OQ870464.1
Pseudomonas aeruginosa strain S2 genomic sequence	Pseudomonas aeruginosa	31002	31002	100%	0.0	99.33%	19212	OQ870447.1
Pseudomonas aeruginosa strain S13 genomic sequence	Pseudomonas aeruginosa	30997	30997	100%	0.0	99.33%	19215	OQ870458.1
Pseudomonas aeruginosa strain S21 genomic sequence	Pseudomonas aeruginosa	30997	30997	100%	0.0	99.33%	19215	OQ870466.1
Pseudomonas aeruginosa strain S5 genomic sequence	Pseudomonas aeruginosa	30033	30033	98%	0.0	98.98%	29483	OQ870450.1
Pseudomonas aeruginosa strain S6 genomic sequence	Pseudomonas aeruginosa	30343	30909	100%	0.0	99.30%	31397	OQ870451.1
Pseudomonas aeruginosa strain S25 genomic sequence	Pseudomonas aeruginosa	30304	30854	100%	0.0	99.26%	32407	OQ870470.1

Supplementary Figure 1: BLASTn search results for the manually identified pyocin-encoding gene cluster (679,269-696,399) in the genome of *P. aeruginosa* ATCC 27853.

Supplementary Figure 2

(A)

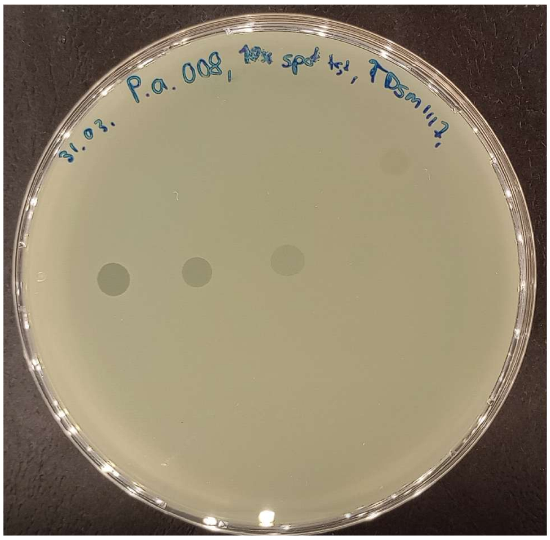

(B)

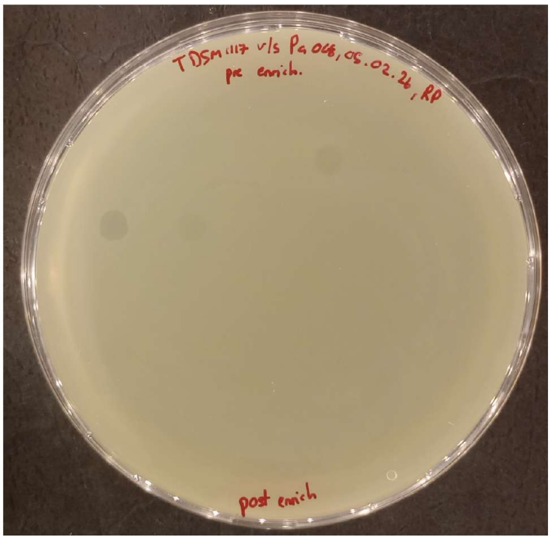

(C)

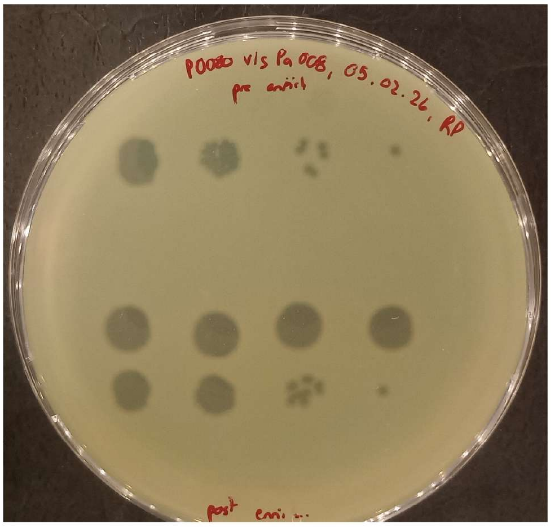

Supplementary Figure 2: (A) Raw image of the serial dilution spot-test depicted in Figure 2. (B) Raw image of the serial dilution spot-tests depicted in Figure 3A. Top half of the plate was used for the “pre-amplification assay” condition and bottom half of the plate was used for the “post-amplification assay” condition. (C) Raw image of the serial dilution spot-tests depicted in Figure 3B. Top half of the plate was used for the “pre-amplification assay” condition and bottom half of the plate was used for the “post-amplification assay” condition.

Supplementary Figure 3

(A)

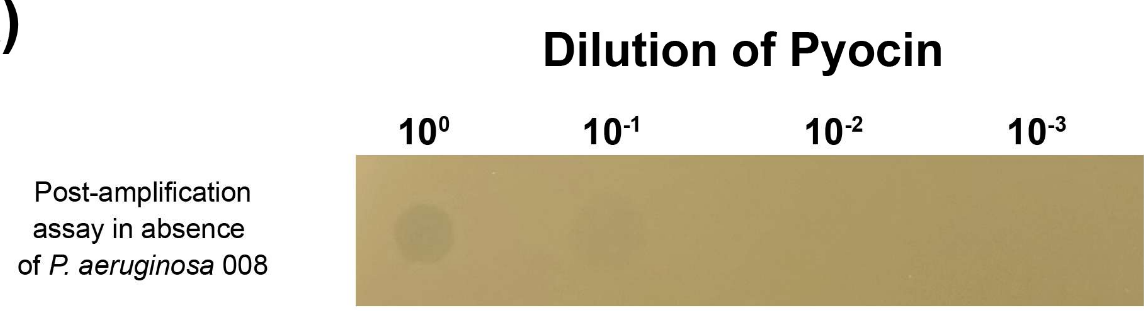

(B)

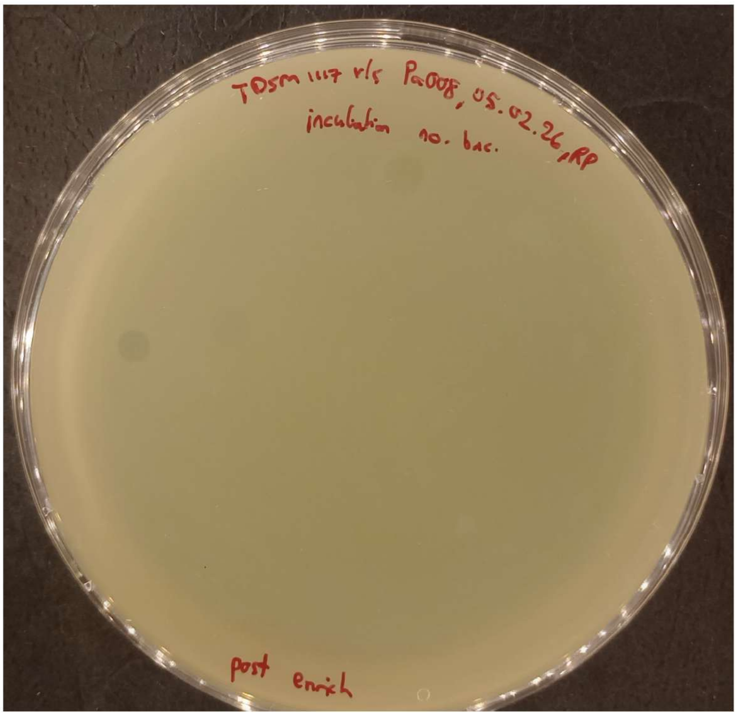

Supplementary Figure 3: (A) Serial dilution spot-test assay confirming that incubation and processing steps involved in the amplification assay itself are not detrimental to the activity of the R1-type pyocin.(B) Raw image of the serial dilution spot test depicted in Supplementary Figure 3A.

Supplementary Figure 4

(A)

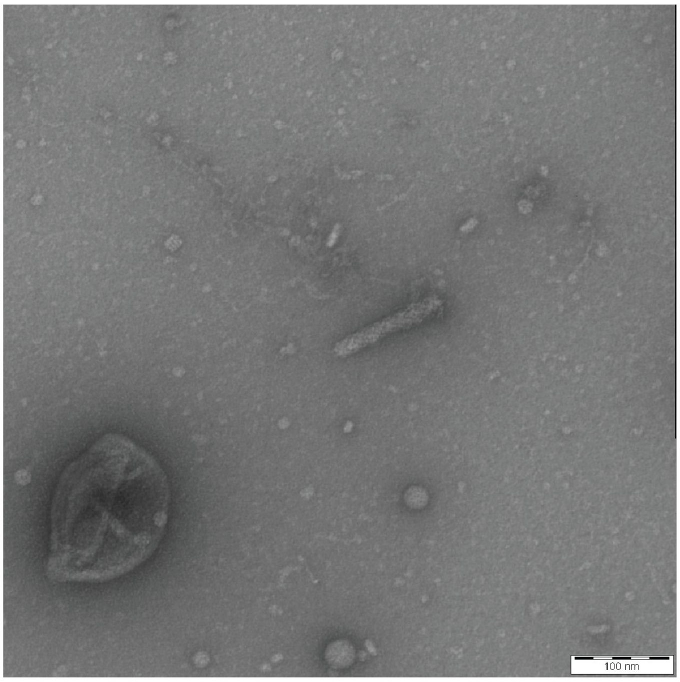

(B)

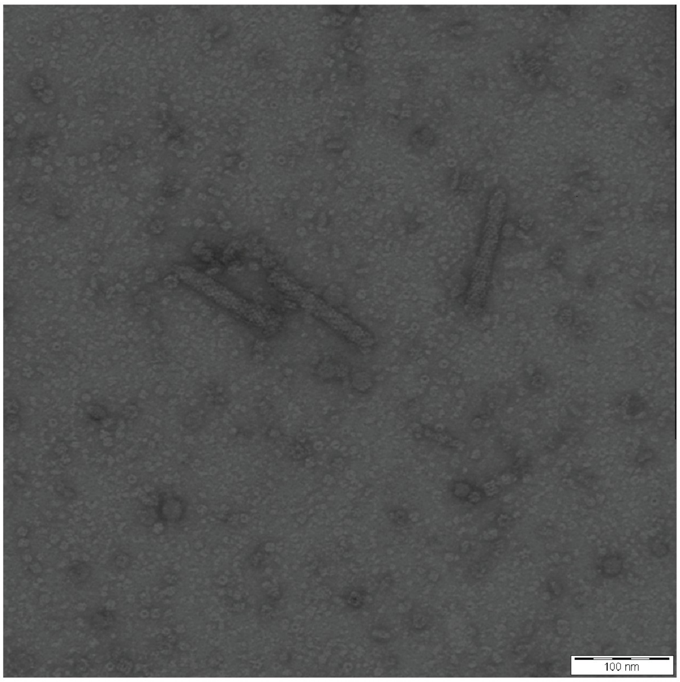

Supplementary Figure 4: (A) Raw transmission electron micrograph of the R1-type pyocin in LB in Figure 4 (left). (B) Raw transmission electron micrograph of the R1-type pyocin in storage buffer in Figure 4 (right).

Supplementary Figure 5

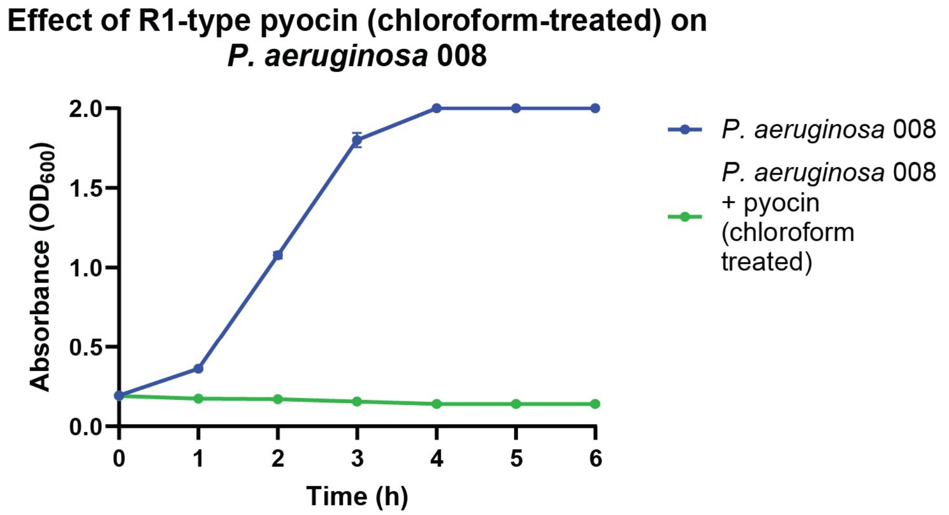

Supplementary Figure 5: Effect of R1-type pyocin (chloroform-treated) against *P. aeruginosa* 008.

Supplementary Information 1: PHASTEST output file of predicted prophage regions and phage-related genes in the 6 predicted prophage regions of *Pseudomonas aeruginosa* ATCC 27853.

Criteria for scoring prophage regions (as intact, questionable, or incomplete):

Method 1:

1. If the number of certain phage organism in this table is more than or equal to 100% of the total number of CDS of the region, the region is marked with total score 150. If less than 100%, method 2 and 3 will be used.

Method 2:

1. If the number of certain phage organism in this table is more than 50% of the total number of CDS of the region, that phage organism is considered as the major potential phage for that region; the percentage of the total number of that phage organism in this table in the total number of proteins of the region is calculated and then multipled by 100; the percentage of the length of that phage organism in this table in the length of the region is calculated and then multipled by 50 (phage head's encapsulation capability is considered).

Method 3:

1. If any of the specific phage-related keywords (such as 'capsid', 'head', 'integrase', 'plate', 'tail', 'fiber', 'coat', 'transposase', 'portal', 'terminase', 'protease' or 'lysin') are present, the score will be increased by 10 for each keyword found.
2. If the size of the region is greater than 30 Kb, the score will be increased by 10.
3. If there are at least 40 proteins in the region, the score will be increased by 10.
4. If all of the phage-related proteins and hypothetical proteins constitute more than 70% of the total number of proteins in the region, the score will be increased by 10.

Compared the total score of method 2 with the total score of method 3, the bigger one is chosen as the total score of the region.

If the region's total score is less than 70, it is marked as incomplete; if between 70 to 90, it is marked as questionable; if greater than 90, it is marked as intact.

In total, 4 intact prophage regions have been identified.

gi|00000000|ref|NC_000000| Pseudomonas aeruginosa strain ATCC 27853 chromosome, complete 6839761, gc%: 66.09%

REGION REGION_LENGTH COMPLETENESS(score) SPECIFIC_KEYWORD REGION_POSITION RNA_NUM TOTAL_PROTEIN_NUM PHAGE_HIT_PROTEIN_NUM HYPOTHETICAL_PROTEIN_NUM PHAGE+HYPO_PROTEIN_PERCENTAGE BACTERIAL_PROTEIN_NUM ATT_SITE_SHOWUP PHAGE_SPECIES_NUM MOST_COMMON_PHAGE_NAME(hit_genes_count) FIRST_MOST_COMMON_PHAGE_NUM FIRST_MOST_COMMON_PHAGE_PERCENTAGE GC_PERCENTAGE

----

1 18.4Kb intact(130) tail,plate 679586-698056 0 24 22 1 95.8% 1 no 15 PHAGE_Pseudo_Dobby_NC_048109(6),PHAGE_Pseudo_phiCTX_NC_003278(6),PHAGE_Entero_Arya_NC_031048(5),PHAGE_Escher_vB_EcoM_ep3_NC_025430(4),PHAGE_Escher_vB_EcoM_ECOO78_NC_041926(4),PHAGE_Vibrio_VP58.5_NC_027981(4),PHAGE_Escher_vB_EcoM_ECO1230_10_NC_027995(4),PHAGE_Pseudo_JBD44_NC_030929(3),PHAGE_Pseudo_PPpW_3_NC_023006(3),PHAGE_Vibrio_vB_VpaM_MAR_NC_019722(3),PHAGE_Pseudo_YMC11/02/R656_NC_028657(3),PHAGE_Klebsi_ST15_OXA48phi14.1_NC_049454(2),PHAGE_Pseudo_PMG1_NC_016765(2),PHAGE_Pseudo_D3_NC_002484(2),PHAGE_Vibrio_VHML_NC_004456(2),PHAGE_Pseudo_MD8_NC_031091(2),PHAGE_Klebsi_ST13_OXA48phi12.1_NC_049453(2),PHAGE_Geobac_E3_NC_029073(1),PHAGE_Pseudo_KPP25_NC_024123(1),PHAGE_Mannhe_vB_MhM_587AP1_NC_028898(1),PHAGE_Halovi_HGTV_1_NC_021328(1),PHAGE_Burkho_phiE255_NC_009237(1),PHAGE_Klebsi_4LV2017_NC_047818(1),PHAGE_Shigel_SfII_NC_021857(1),PHAGE_Pseudo_PAJU2_NC_011373(1),PHAGE_Burkho_BcepMu_NC_005882(1),PHAGE_Klebsi_ST437_OXA245phi4.1_NC_049448(1),PHAGE_Salmon_SW9_NC_049459(1),PHAGE_Pseudo_phiR18_NC_041964(1),PHAGE_Pseudo_H66_NC_042342(1),PHAGE_Burkho_AP3_NC_047752(1),PHAGE_Burkho_KL3_NC_015266(1),PHAGE_Shigel_SfIV_NC_022749(1),PHAGE_Escher_500465_2_NC_049343(1),PHAGE_Erwini_ENT90_NC_019932(1),PHAGE_Bordet_vB_BbrM_PHB04_NC_047861(1),PHAGE_Klebsi_ST512_KPC3phi13.2_NC_049452(1),PHAGE_Salmon_SEN5_NC_028701(1),PHAGE_Pseudo_F116_NC_006552(1),PHAGE_Escher_pro483_NC_028943(1),PHAGE_Escher_500465_1_NC_049342(1),PHAGE_Burkho_AH2_NC_018283(1),PHAGE_Salmon_SEN4_NC_029015(1),PHAGE_Mannhe_phiMHaA1_NC_008201(1),PHAGE_Pseudo_phi2_NC_030931(1),PHAGE_Salmon_SP_004_NC_021774(1),PHAGE_Klebsi_ST437_OXA245phi4.2_NC_049449(1) 4 25% 65.02%

2 40.4Kb intact(114) transposase,integrase,portal,head,virion,tail 796824-837266 0 54 53 1 100% 0 yes 10 PHAGE_Pseudo_JBD25_NC_027992(42),PHAGE_Pseudo_JBD18_NC_027986(35),PHAGE_Pseudo_B3_NC_006548(33),PHAGE_Pseudo_JBD67_NC_042135(32),PHAGE_Pseudo_vB_PaeS_PM105_NC_028667(30),PHAGE_Pseudo_JD024_NC_024330(4),PHAGE_Pseudo_MP38_NC_011611(4),PHAGE_Pseudo_H70_NC_027384(4),PHAGE_Escher_D108_NC_013594(4),PHAGE_Ralsto_RS138_NC_029107(3),PHAGE_Pseudo_JBD30_NC_020198(3),PHAGE_Pseudo_JBD5_NC_020202(3),PHAGE_Burkho_BcepMu_NC_005882(3),PHAGE_Pseudo_JBD88a_NC_020200(2),PHAGE_Pseudo_MP29_NC_011613(2),PHAGE_Rhodov_RS1_NC_020866(2),PHAGE_Pseudo_LPB1_NC_027298(2),PHAGE_Entero_Mu_NC_000929(2),PHAGE_Pseudo_FHA0480_NC_041851(2),PHAGE_Rhodob_RC1_NC_020839(2),PHAGE_Pseudo_JBD93_NC_030918(2),PHAGE_Pseudo_JBD69_NC_030908(2),PHAGE_Burkho_AP3_NC_047752(1),PHAGE_Pseudo_D3_NC_002484(1),PHAGE_Pseudo_D3112_NC_005178(1),PHAGE_Pseudo_PAK_P5_NC_022966(1),PHAGE_Burkho_phi1026b_NC_005284(1),PHAGE_Pseudo_PA1phi_NC_023700(1),PHAGE_Vibrio_12B12_NC_021070(1),PHAGE_Pseudo_vB_PaeS_PAO1_Ab30_NC_026601(1),PHAGE_Burkho_KS5_NC_015265(1),PHAGE_Entero_SfMu_NC_027382(1),PHAGE_Pseudo_PMG1_NC_016765(1),PHAGE_Burkho_phiE125_NC_003309(1),PHAGE_Staphy_SPbeta_like_NC_029119(1),PHAGE_Aeromo_65_NC_015251(1),PHAGE_Haemop_SuMu_NC_019455(1),PHAGE_Pseudo_MP48_NC_024782(1),PHAGE_Rhodob_RcapMu_NC_016165(1),PHAGE_Pseudo_DMS3_NC_008717(1),PHAGE_Rhizob_RR1_B_NC_021557(1),PHAGE_Burkho_phiE255_NC_009237(1),PHAGE_Burkho_KS9_NC_013055(1),PHAGE_Pseudo_JBD24_NC_020203(1),PHAGE_Stenot_Smp131_NC_023588(1),PHAGE_Mannhe_vB_MhM_3927AP2_NC_028766(1),PHAGE_Vibrio_VP882_NC_009016(1) 25 77.77% 62.93%

3 42.9Kb intact(110) integrase,lysis,tail,portal,head,capsid 1337187-1380105 0 65 59 6 100% 0 yes 18 PHAGE_Pseudo_MD8_NC_031091(41),PHAGE_Pseudo_F10_NC_007805(28),PHAGE_Pseudo_phi2_NC_030931(26),PHAGE_Pseudo_YMC11/02/R656_NC_028657(5),PHAGE_Pseudo_phi297_NC_016762(4),PHAGE_Pseudo_PMG1_NC_016765(4),PHAGE_Pseudo_vB_PaeP_Tr60_Ab31_NC_023575(4),PHAGE_Pseudo_vB_PaeM_PAO1_Ab03_NC_026587(3),PHAGE_Pseudo_D3_NC_002484(3),PHAGE_Entero_c_1_NC_019706(3),PHAGE_Pseudo_JBD44_NC_030929(3),PHAGE_Pseudo_PAJU2_NC_011373(3),PHAGE_Entero_N15_NC_001901(2),PHAGE_Phage_Gifsy_2_NC_010393(2),PHAGE_Pseudo_phiR18_NC_041964(2),PHAGE_Entero_HK97_NC_002167(2),PHAGE_Entero_HK629_NC_019711(2),PHAGE_Entero_mEp235_NC_019708(2),PHAGE_Pseudo_F116_NC_006552(2),PHAGE_Entero_mEp213_NC_019720(2),PHAGE_Entero_HK106_NC_019768(2),PHAGE_Pseudo_KPP25_NC_024123(2),PHAGE_Entero_DE3_NC_042057(2),PHAGE_Cronob_ENT47670_NC_019927(1),PHAGE_Idioma_Phi1M2_2_NC_025471(1),PHAGE_Salmon_118970_sal4_NC_030919(1),PHAGE_Pseudo_R12_NC_048662(1),PHAGE_Rhodob_RcRhea_NC_028954(1),PHAGE_Edward_eiAU_183_NC_023555(1),PHAGE_Entero_phiP27_NC_003356(1),PHAGE_Edward_GF_2_NC_026611(1),PHAGE_Entero_HK022_NC_002166(1),PHAGE_Ralsto_RS138_NC_029107(1),PHAGE_Salmon_epsilon15_NC_004775(1),PHAGE_Entero_mEp460_NC_019716(1),PHAGE_Salmon_SEN22_NC_028696(1),PHAGE_Entero_lambda_NC_001416(1),PHAGE_Shewan_1/44_NC_025463(1),PHAGE_Salmon_SI7_NC_049460(1),PHAGE_Serrat_Parlo_NC_048758(1),PHAGE_Pseudo_PS_1_NC_029066(1),PHAGE_Pseudo_SM1_NC_041877(1),PHAGE_Mannhe_vB_MhS_587AP2_NC_028743(1),PHAGE_Pseudo_phiPSA1_NC_024365(1),PHAGE_Aurant_AmM_1_NC_027334(1),PHAG

E_Rhodob_RcCronus_NC_042049(1),PHAGE_Pseudo_nickie_NC_042091(1),PHAGE_Pseudo_PPpW_3_NC_023006(1),PHAGE_Entero_mEp237_NC_019704(1),PHAGE_Pseudo_MP42_NC_018274(1),PHAGE_Entero_IME10_NC_019501(1),PHAGE_Edward_eiAU_NC_042029(1),PHAGE_Salmon_SEN34_NC_028699(1),PHAGE_Pseudo_DMS3_NC_008717(1),PHAGE_Burkho_BcepC6B_NC_005887(1) 17 63.07% 61.49%

4 55.2Kb questionable(84) head,coat,tail 2508737-2564035 0 68 60 6 97% 2 yes 19 PHAGE_Pseudo_phi297_NC_016762(39),PHAGE_Pseudo_YMC11/07/P54_PAE_BP_NC_030909(36),PHAGE_Pseudo_JBD44_NC_030929(18),PHAGE_Pseudo_PMG1_NC_016765(14),PHAGE_Pseudo_D3_NC_002484(13),PHAGE_Pseudo_H66_NC_042342(10),PHAGE_Pseudo_F116_NC_006552(8),PHAGE_Pseudo_YMC11/02/R656_NC_028657(5),PHAGE_Pseudo_PAJU2_NC_011373(4),PHAGE_Salmon_epsilon15_NC_004775(3),PHAGE_Pseudo_F10_NC_007805(3),PHAGE_Pseudo_phi2_NC_030931(2),PHAGE_Mannhe_vB_MhS_587AP2_NC_028743(2),PHAGE_Pseudo_phiPSA1_NC_024365(2),PHAGE_Ralsto_RsoM1USA_NC_049432(2),PHAGE_Pseudo_vB_PaeP_Tr60_Ab31_NC_023575(2),PHAGE_Flavob_Fpv5_NC_031921(1),PHAGE_Phage_Gifsy_2_NC_010393(1),PHAGE_Thermu_OH2_NC_021784(1),PHAGE_Pseudo_phiR18_NC_041964(1),PHAGE_Bacill_1_NC_009737(1),PHAGE_Pseudo_Dobby_NC_048109(1),PHAGE_Sinorh_PBC5_NC_003324(1),PHAGE_Clostr_phiCT19406B_NC_030947(1),PHAGE_Salmon_g341c_NC_013059(1),PHAGE_Clostr_phiCT19406A_NC_030950(1),PHAGE_Flavob_Fpv10_NC_031932(1),PHAGE_Flavob_Fpv11_NC_031945(1),PHAGE_Pseudo_MD8_NC_031091(1),PHAGE_Phage_Gifsy_1_NC_010392(1),PHAGE_Cronob_phiES15_NC_018454(1),PHAGE_Pseudo_phiCTX_NC_003278(1),PHAGE_Flavob_Fpv8_NC_031919(1),PHAGE_Stenot_vB_SmaS_DLP_5_NC_042082(1),PHAGE_Pseudo_AF_NC_019923(1),PHAGE_Geobac_GBSV1_NC_008376(1),PHAGE_Gordon_BetterKatz_NC_031247(1),PHAGE_Salmon_SPN3UB_NC_019545(1),PHAGE_Mycoba_Sandalphon_NC_051706(1),PHAGE_Mycoba_Bipper_NC_031253(1),PHAGE_Rhizob_RR1_A_NC_021560(1),PHAGE_Lactob_LL_H_NC_009554(1),PHAGE_Pseudo_O4_NC_031274(1),PHAGE_Pseudo_KPP25_NC_024123(1),PHAGE_Burkho_vB_BmuP_KL4_NC_047958(1),PHAGE_Xylell_Xfas53_NC_013599(1),PHAGE_Clostr_phiCTC2B_NC_030951(1),PHAGE_Myxoco_Mx8_NC_003085(1),PHAGE_Arthro_Vibaki_NC_049465(1),PHAGE_Flavob_Fpv7_NC_031905(1),PHAGE_Clostr_phiCTC2A_NC_030949(1),PHAGE_Mannhe_vB_MhS_1152AP2_NC_028956(1),PHAGE_Pseudo_PA11_NC_007808(1),PHAGE_Salmon_SEN34_NC_028699(1),PHAGE_Klebsi_ST437_OXA245phi4.1_NC_049448(1) 18 57.35% 62.55%

5 49.9Kb intact(150) portal,terminase,capsid,head,tail,lysis,virion,plate,integrase,coat 5102870-5152806 0 59 50 6 94.9% 3 yes 10 PHAGE_Pseudo_Dobby_NC_048109(35),PHAGE_Pseudo_phiCTX_NC_003278(34),PHAGE_Burkho_KS14_NC_015273(12),PHAGE_Pseudo_Pf1_NC_001331(7),PHAGE_Stenot_Smp131_NC_023588(6),PHAGE_Burkho_AP3_NC_047752(6),PHAGE_Burkho_phiE12_2_NC_009236(5),PHAGE_Ralsto_RsoM1USA_NC_049432(5),PHAGE_Burkho_KL3_NC_015266(5),PHAGE_Pseudo_phi3_NC_030940(4),PHAGE_Ralsto_RSY1_NC_025115(4),PHAGE_Burkho_KS5_NC_015265(4),PHAGE_Burkho_phiE202_NC_009234(4),PHAGE_Ralsto_RSA1_NC_009382(3),PHAGE_Erwini_ENT90_NC_019932(3),PHAGE_Salmon_RE_2010_NC_019488(3),PHAGE_Salmon_Fels_2_NC_010463(3),PHAGE_Salmon_SEN8_NC_047753(3),PHAGE_Serrat_Parlo_NC_048758(2),PHAGE_Rhizob_RR1_A_NC_021560(2),PHAGE_Salmon_SW9_NC_049459(2),PHAGE_Burkho_phi52237_NC_007145(2),PHAGE_Bordet_vB_BbrM_PHB04_NC_047861(2),PHAGE_Pseudo_H66_NC_042342(2),PHAGE_Escher_pro483_NC_028943(2),PHAGE_Erwini_EtG_NC_047833(2),PHAGE_Escher_500465_1_NC_049342(2),PHAGE_Pseudo_KPP25_NC_024123(1),PHAGE_Klebsi_3LV2017_NC_047817(1),PHAGE_Faecal_FP_Lugh_NC_047912(1),PHAGE_Erwini_PEp14_NC_016767(1),PHAGE_Pseudo_PPpW_3_NC_023006(1),PHAGE_Dickey_vB_DsoM_JA29_NC_048053(1),PHAGE_Pseudo_PMG1_NC_016765(1),PHAGE_Erwini_vB_EamM_Yoloswag_NC_047815(1),PHAGE_Shigel_SfII_NC_021857(1),PHAGE_Klebsi_4LV2017_NC_047818(1),PHAGE_Entero_186_NC_001317(1),PHAGE_Vibrio_vB_VpaM_MAR_NC_019722(1),PHAGE_Entero_PsP3_NC_005340(1),PHAGE_Pseudo_C5a_NC_047790(1),PHAGE_Mycoba_Butters_NC_021061(1),PHAGE_Entero_mEp390_NC_019721(1),PHAGE_Entero_phi80_NC_021190(1),PHAGE_Lactob_JCL1032_NC_019456(1),PHAGE_Entero_P4_NC_001609(1),PHAGE_Sinorh_PBC5_NC_003324(1),PHAGE_Dickey_vB_DsoM_JA11_NC_048077(1),PHAGE_Pseudo_phiR18_NC_041964(1),PHAGE_Escher_500465_2_NC_049343(1),PHAGE_Bacill_vB_BceS_MY192_NC_048633(1),PHAGE_Klebsi_ST13_OXA48phi12.1_NC_049453(1),PHAGE_Salmon_SP_004_NC_021774(1),PHAGE_Salmon_SEN1_NC_029003(1) 27 59.32% 61.85%

6 15.1Kb questionable(85) integrase,coat,recombinase 5787383-5802527 1 19 15 3 94.7% 1 no 7 PHAGE_Pseudo_Pf1_NC_001331(8),PHAGE_Gordon_Daredevil_NC_048021(2),PHAGE_Lactob_Lj965_NC_005355(2),PHAGE_Methan_psiM100_NC_002628(2),PHAGE_Aeromo_Aes012_NC_020879(1),PHAGE_Pseudo_phi3_NC_030940(1),PHAGE_Klebsi_4LV2017_NC_047818(1),PHAGE_Entero_186_NC_001317(1),PHAGE_Azospi_Cd_NC_010355(1),PHAGE_Rhodoc_E3_NC_021347(1),PHAGE_Klebsi_ST13_OXA48phi12.1_NC_049453(1),PHAGE_Klebsi_ZCKP1_NC_047994(1),PHAGE_Temper_phiNIH1.1_NC_003157(1),PHAGE_Aeromo_phiO18P_NC_009542(1),PHAGE_Haemop_HP2_NC_003315(1),PHAGE_Erwini_EtG_NC_047833(1),PHAGE_Erwini_ENT90_NC_019932(1),PHAGE_Strept_315.4_NC_004587(1),PHAGE_Methan_psiM2_NC_001902(1) 7 42.1%

gi|00000000|ref|NZ_CP101912.1| Pseudomonas aeruginosa strain ATCC 27853 chromosome, complete 6839761, gc%: 66.09%

CDS_POSITION BLAST_HIT EVALUE PID GO_COMPONENT GO_FUNCTION GO_PROCESS prophage_PRO_SEQ

#### region 1 ####

complement(679586..679900) PHAGE_Burkho_AH2_NC_018283: excisionase; PP_00628; phage 1.06e-07 prtN NONE NONE NONE
MQPSIAPSTPIPRQETVELVYRIFGDVLVPLEQVRERWFRNLNKENFSKALACGRIALPVTTLDDSHKAMQFVALDHLAAYVDQRANQAGGARRAQPGADSIAS

complement(680000..680770) PHAGE_Pseudo_phi2_NC_030931: HTH-type transcriptional regulator PrtR; PP_00629; phage 2.21e-93 prtR NONE NONE NONE
MDKSTQIPPDSFAARLKQAMAMRNLKQETLAEAAGVSQNTIHKLTSGKAQSTRKLIEIAAALGVSPVWLQTGEGAPAARSAVSVADGSPLVLEPLHPWDSDTPLDEDEVELPLYKEVEMSAGAGRTAVREIEGRKLRFSYATLRASGVDPSAAICAQLTGNSMEPLIMDGSTIGVDTATTHITDGEIYALEHDGMLRVKFVYRLPGGGIRLRSFNREEYPDEEYSPEDMRSRQISMIGWVFWWSTVRHRRGPSLVR

681228..681428 PHAGE_Pseudo_Dobby_NC_048109: TraR/DksA family transcriptional regulator; PP_00630; phage 4.84e-11 - NONE GO:0008270 - zinc ion binding [Evidence IEA]; NONE
MADLADHANELVLARLDGLLAARPALAIRESAEDCEDCGEPIPQARRRAAPGCSRCIDCQDRHERR

681476..681835 hypothetical protein; PP_00631 N/A - NONE NONE NONE
MIKAIDEMLKLWAEEMHAPGSNGGGYAGGNLIAMLIASKGEVVRGHRGSRVILDRVAEVDRLVNRLPEELKNVVVEHYLNRDSFPEQKYRHCGCSRNTFYLRLHVAHQGIQDGLLRRAA

682294..682647 phage holin family protein; PP_00632 N/A - NONE NONE NONE
MGNEPQTLTEMPLWVLILLAALGGVSGEMWRADKAGLGGWALLRRLALRSGASIVCGVAVMLLALACGAALLFAAALGSLTAAAGAEIAVGLYERWAARRLGVCELPEEQSDDRGPH

682669..683184 PHAGE_Entero_Arya_NC_031048: putative minor tail protein; PP_00633; phage 7.27e-08 - NONE NONE NONE
MPEQAVTLEALYAAIEQVLRERLPEAQLIGFWPGVPENTPAVSLEIAELLPERDPGTGESALLCRLQARIMVPPGADRQAVSIACGIVRTLREQTWNLSLEPARFVRSAVDGSREELKSLRVWLVEWTQSLRLGDPEWAWEDQPPGSLMLGFDPQTGPGHEPDYFAPEALA

683181..683738 PHAGE_Pseudo_PPpW_3_NC_023006: putative baseplate assembly protein V; PP_00634; phage 3.46e-26 - NONE NONE NONE
MSYVSAEHDRMLAAMILPCVVVAVDLAAARVRVRSGDWTSGWLRWHSLAAGKVRHWRAPSIGEQGTLLSPSGGVSMGTFIPGLYGDAGTAPDNSASSETWRFDDGASLSYDWAAHRYRVELPSGTVEVKVGASEVRVSDGAVSLKAPKISLEGPVEIAGTLTVSGDILGGGSIIDTAGNSNHHTH

683891..684217 PHAGE_Entero_Arya_NC_031048: putative baseplate assembly protein W; PP_00635; phage 4.21e-25 - NONE NONE NONE
MIGMDRRSGLPLSGLAHLKQSVEDILTTPLGSRRMRPEYGSKLRRMVDMPVSEGWKSAVQAEVARSLGRWEPRIALSAVRVVAVVDGRVDLLLSGVFEGENINMEVSA

684214..685101 PHAGE_Klebsi_4LV2017_NC_047818: baseplate assembly protein; PP_00636; phage 4.24e-119 - NONE NONE NONE
MIIDLSQLPEPEVIENLDFETIYQELLGDFREAMAGEWTAEVESDPVLKLLQLAAYRELLLRARINDAARAVMLAYASGADLDQIGAGFNVQRLLIRPAQPEAVPPVEAQYESDKSLRNRIQLAFEQLSVAGPRNAYIAHALGADGRVADASATSPAPCEVLISVLGVEGNGQAPETVLQAVRLALNAEDVRPVADRVTVRSAGIVPYQVKAQLYLFPGPEAELIRAAAEASLRDYISAQRRLGRDIRRSALFATLHVEGVQRVELQEPAADVVLDETQAAYCTGYAITLGGVDE

685094..685627 PHAGE_Pseudo_Dobby_NC_048109: tail protein I; PP_00637; phage 1.00e-49 - GO:0044423 - virion component [Evidence IEA]; GO:0005198 - structural molecule activity [Evidence IEA]; GO:0016032 - viral process [Evidence IEA];
MSSRLLPPNRSSLERSLGDVLPAELPVPLRELNDPARCEAALLPYLAWTRSVDRWDPDWSDEAKRNAVATSFVLHQRKGTLTALRQVVEPIGALSEVTEWWQRSPLGVPGTFEITVDVSDRGIDEGTVLELERLLDDVRPVSRHLTRLDLRITPVIRSRHGLAVTDGDTLEIFPWKQ

685629..687734 PHAGE_Pseudo_Dobby_NC_048109: tail fiber protein; PP_00638; phage 2.23e-38 - NONE NONE NONE
MTTNTPKYGGLLTDIGAAALAAASAAGKKWQPTHMLIGDAGGAPGDTPDPLPSAAQKSLINQRHRAQLNRLFVSDKNANTLVAE

VVLPVEVGGFWIREIGLQDADGKFVAVSNCPPSYKAAMESGSARTQTIRVNIALSGLENVQLLIDNGIIYATQDWVKEKVAADFKG
RKILAGNGLVGGGDLSADRSIGLAPSGVTAGSYRSVTVNANGVVTQGSNPTTLAGYAIGDAYTKADTDGKLAQKANKATTLAGY
GITDALRVDGNAVSSSRLAAPRSLAASGDASWSVTFDGSANVSAPLSLSATGVAAGSYPKVTVDTKGRVTAGMALAATDIPGLD
ASKLVSGVLAEQRLPVFARGLATAVSNSSDPNTATVPLMLTNHANGPVAGRYFYIQSMFYPDQNGNASQIATSYNATSEMYVRV
SYAANPSIREWLPWQRCDIGGSFTKTTDGSIGNGVNINSFVNSGWWLQSTSEWAAGGANYPVGLAGLLIVYRAHADHIYQTYVT
LNGSTYSRCCYAGSWRPWRQNWDDGNFDPASYLPKAGFTWAALPGKPATFPPSGHNHDTSQITSGILPLARGGLGANTAAGA
RNNIGAGVPATASRALNGWWKDNDTGLIVQWMQVNVGDHPGGIIDRTLTFPIAFPSACLHVVPTVKEVGRPATSASTVTVADVS
VSNTGCVIVSSEYYGLAQNYGIRVMAIGY

687742..688182 PHAGE_Pseudo_Dobby_NC_048109: tail protein; PP_00639; phage 6.09e-31
- NONE NONE NONE
MIFFHAATGGFYSKEIHGSRMPLEDEMHPLEDAEYQALLRAQSEGKRIVTDHTGRPICVDPPAPAKDILVQRERIWRDRQLQLTD
GPLARHRDEQDLGKTTTLSQEQLRELTLYRAVLRDWPIAAEFPDLNARPEPPAWLQSLITP

688225..689385 PHAGE_Vibrio_vB_VpaM_MAR_NC_019722: tail sheath protein; PP_00640; phage
3.86e-108 - NONE NONE NONE
MSFFHGVTVTNVDIGARTIALPASSVIGLCDVFTPGAQASAKPNVPVLLTSKKDAAAAFGIGSSIYLACEAIYNRAQAVIVAVGVEAA
ETPEAQASAVIGGISAAGERTGLQALLDGKSRFNAQPRLLVAPGHSAQQAVATAMDGLAEKLRAIAILDGPNSTDEAAVAYAKNF
GSKRLFMVDPGVQVWDSATNAARNAPASAYAAGLFAWTDAEYGFWSSPSNKEIKGVTGTSRPVEFLDGDETCRANLLNNANIA
TIIRDDGYRLWGNRTLSSDSKWAFVTRVRTMDLVMDAILAGHKWAVDRGITKTYVKDVTEGLRAFMRDLKNQGAVINFEVYADP
DLNSASQLAQGKVYWNIRFTDVPPAENPNFRVEVTDQWLTEVLDVA

689398..689901 PHAGE_Escher_vB_EcoM_ep3_NC_025430: tail protein; PP_00641; phage 5.27e-41
- GO:0044423 - virion component [Evidence IEA]; GO:0005198 - structural molecule activity [Evidence IEA];
GO:0016032 - viral process [Evidence IEA];
MIPQTLTNTNLFIDGVSFAGDVPSLTLPKLAVKTEQYRAGGMDAPVSIDMGLEAMEAKFSTNGARREALNFFGLADQSAFNGVFR
GSFKGQKGASVPLVATLRGLLKEVDPGDWKAGEKAEFKYAVAVSYYKLEVDGREVYEIDPVNGVRAINGVDQLAGMRNDLGL

689916..690260 PHAGE_Escher_vB_EcoM_ECOO78_NC_041926: hypothetical protein; PP_00642; phage
5.82e-14 - NONE NONE GO:0098003 - viral tail assembly [Evidence
IEA];
MTQENRLPGWLTLDADAALVRLSRPAQCNGVSVDTLTLRAPTVRDIRLAGKVAGDDAEERELQLFASLAQVSRQDLEGLKLSDY
QRLQGAYFRLVQDDTDDTFAYASTGEAPGH

690430..692667 PHAGE_Burkho_BcepMu_NC_005882: gp44; PP_00643; phage 1.41e-08
- NONE NONE NONE
MSKDMDLVVSIGGIADPSLGKAFETVKARLDSLQERARQASSLRDVLGDAIRLERELADMRKVGDRGVAEHARQLGERQEQLKR
LGIEARAAGDAYARLGEMQRGLDMQVRGLQRLEQASQAMPLASAFSGLVVEASKTAAGYQARLRDLAIRNGLDAGREPALASLI
QDSASQSGLGRTATLDMLEHLNATGMGFAAAQMNLGLAGRFGFGQGIASAEAAGLVRALQLAQGSDSPEQLSASLDRLVVLGK
GRVGSEALARRLPALLSALGNAGEATAGDVGALGALLEIQAKNTTPDKADVRMKAWLEFVGSGSLKRAYGQDYDRDLEALRKD
GASLLEANLELAARYRDKGGKLSAGVASPALEAYRASRGEFQGLLESQQSSVGSSERDAQRRKGMSQELWKASSDSWERAQT
ALGSALNPYLDNLAKGSKALGESTAELLEAYPRTTAGLTAAAGAVLSGYLAYKGGRGAIDVLRGGRLGRRGTAAVGDLIERGAGR
VSGGSEIQRVFVTNWPVPGGDSTLESARRPAQRKRGQTPRRKRGKGGGLKARSLPSLGFSAGGGLGAMAGKLPRLSRLPIRNA
PLQVASSLIDVAEVYSSDLSESEKTVAYGEAGGSLAGSLAGAALGASIGSVVPVVGTLIGGLVGGAIGAWGGSELGGRLGRSLAG
DPPAASDNKPAVAVPQAGPVAAAPNWTFAPQINLTVQGNVHEPQRLADELLPYLQRMLVDFADERQRRSLYDPAMV

692677..693549 PHAGE_Escher_vB_EcoM_ECOO78_NC_041926: putative tail protein; PP_00644; phage
1.03e-23 - NONE NONE NONE
MAYLEQLQAGLRYLGRAGESGRKSLDKVVAPVNGAISEIRGAAAELENLPGVSPEMAARLQRAMRGIGQAQGKVNRVVSTYDR
ASRALLGIDERLDALKVQVNSAAQAVGKVAGDISPTLAGVLPSWLLAPSATPPSEAAASLPHLLVLQPLTANAQPFYFNLNTAAFD
ALQRNSAYNWSGQVRLGRRPALQSVGMGEESILLKGAVFPLRRQVGNQEKVVGLEQLEALRRLAERREPLILSSGYGEVQMGL
WCLVRISENQSALLGNGAPRKQTFDLEFKRYGDDLPNR

693524..693730 PHAGE_Klebsi_ST15_OXA48phi14.1_NC_049454: tail protein; PP_00645; phage 1.28e-
09 - NONE NONE NONE
MATTCRTADGDMLDSLCYHVYGHLLGCVEATLDANPGLADEQQPFRAGLLISFPDMPVVNVEQVRLWD

693788..694777 PHAGE_Vibrio_vB_VpaM_MAR_NC_019722: putative tail protein; PP_00646; phage 1.23e-
56 - NONE NONE NONE
MQPSFRIVADGTDVTQRLNDRLLKLTLLDKPGMESDSLTLRIDDRDGQVALPRRGAVLEVHLGYAGEPLMRMGRFTVDTLQWA
GPPDCLTVTAKAGDMRGSGKTIRSGGWEGTTLAQVCRDVGARNGWRVECPLQVAIARVDQVNESDYHFVTRLARQYDCTAKL
AEGMLMVLPRQSGQSATGRRIEPLVLGRADVGSFDVTFDDRSLMRTVKTRYQLPGSGEVKSVELKNPKAPATAMGEHVDRHLY
TSRGEAEQAAKARLASFSRSSASVRLELPGRGDLFAERSLLLQGFKAGIDGEFLIDSVEHTYSSSGWTTVVQCNGGRGGKG

694810..695439 PHAGE_Pseudo_JBD44_NC_030929: lytic enzyme; PP_00647; phage 1.01e-100
- NONE NONE NONE
MKLTEQQLLRIFPNARLVAGVFVVALQRAMDEREIDTPARRAAFLAQVGHESSQLTRLVENLNYSAQGLAATWPGRYLGPDGQP
NALALRLARNPQAIADNTYATRNGNGDEASGDGWRFRGRGLLQITGRANYRLVGEALGEPLEAEPWRLEQPVPAARSAAWWW
AGHGLNELADRGEFAAITRRINGGLNGQAERLALWQRARAVLS

695436..695798 PHAGE_Pseudo_D3_NC_002484: hypothetical protein; PP_00648; phage 3.30e-19
- NONE NONE NONE

MSRLALLLPAVLLVLLAGALLGGGLVARHYRPQLEEALGQLTASRVASGQLEALLDEQQRALAAVRASAERRAKDVEQALGEAR AQAAEQYAAAVRLLQEPDIGVDCQAAGAAIDRELGL

695795..696052 PHAGE_Pseudo_JBD44_NC_030929: hypothetical protein; PP_00649; phage 5.95e-26 - NONE NONE NONE
MTRLLLGLCLLFAGCAASPTTPRPVRVEVPLAVPCRVPDVRPPSWAGATLKAGDSLQAKVRALLAERRQRQGYELELQAALRAC R

696400..697005 PHAGE_Halovi_HGTV_1_NC_021328: carbamoyl phosphate synthase small subunit; PP_00650; phage 1.07e-08 - NONE NONE NONE
MLLMIDNYDSFTYNLVQYFGELKAEVKVVRNDELSVEQIEALAPERIVLSPGPCTPNEAGVSLAVIERFAGKLPLLGVCLGHQSIG QAFGGEVVRARQVMHGKTSPIHHKDLGVFAGLANPLTVTRYHSLVVKRESLPECLEVTAWTQHADGSLDEIMGVRHKTLNVEGV QFHPESILTEQGHELLANFLRQQGGVRGEGN

697007..698056 PHAGE_Geobac_E3_NC_029073: putative pyrimidine nucleoside phosphorylase; PP_00651; phage 4.99e-05 trpD NONE GO:0004048 - anthranilate phosphoribosyltransferase activity [Evidence IEA]; GO:0000162 - tryptophan biosynthetic process [Evidence IEA];
MDIKGALNRIVNQLDLTTEEMQAVMRQIMTGQCTDAQIGAFLMGMRMKSETIDEIVGAVAVMRELADGVQLPTLKHVVDVVGTG GDGANIFNVSSAASFVVAAAGGKVAKHGNRAVSGKSGSADLLEAAGIYLELTSEQVARCIDTVGVGFMFAQVHHKAMKYAAGPR RELGLRTLFNMLGPLTNPAGVRHQVVGVFTQELCKPLAEVLKRLGSEHVLVVHSRDGLDEFSLAAATHIAELKDGEVREYEVRPE DFGIKSQTLMGLEVDSPQASLELIRDALGRRKTEAGQKAAELIVMNAGPALYAADLATSLHEGIQLAHDALHTGLAREKMDELVAF TAVYREENAQ

#### region 2 ####

complement(796824..797192) PHAGE_Pseudo_JBD25_NC_027992: mor transcription activator family protein; PP_00743; phage 8.49e-85 - NONE NONE NONE
MKQSSILAETRHELLDDIAAHTATVLSEHGIDAGLAEQAGHAVADHLANQWRGATLYIPSDYRHQVTKRDLQILSEFNGRNHHAL ARKYGLTPSSIYKLLKRIQDRKFERDQGKLDLGDGLA

complement(797189..797737) PHAGE_Pseudo_JBD25_NC_027992: hypothetical protein; PP_00744; phage 1.60e-130 - NONE NONE NONE
MQLQCPCCGEQFPVEAGFADTDGKRLAALFAGLDPKLGRAILNYLRLFSPAKRGLRMTRAIKLVEELLNLVNTGTVQKDARSNDT KPASPRLWTTGIEQMITGRERLQLPLENHNYLRAVVWGLASDPAQALAASSKRPQAGGPSTQQLLQDQVGRIQSDIVLGLITKED GERQIAALKGGA

complement(797737..798303) PHAGE_Pseudo_JBD25_NC_027992: PF06252 family protein; PP_00745; phage 6.78e-131 - NONE NONE NONE
MALARGLLSKIHIARQQLGLQDDVYRQKLQVMFGKGSARDLNLRQAEQLLTEFKRLGWQPQPSKRAAGKPHNWRQLPAEVEVI EAQLTNMGLPWSYADAIAKRQFGVAKVAWLKKPEQLKAVLAALHVEQEKRGLLGSVEELLKLLGEHDPNWRVDLEHLPKGWER RRPILKSLVETLRAAASARGLL

complement(798290..798757) PHAGE_Pseudo_JBD25_NC_027992: hypothetical protein; PP_00746; phage 4.76e-110 - NONE NONE NONE
MERYHSTAGDPPRRDADVKRQEAQELDELVQQFLAGGGQIEKVGYKMRELPDTFVINPMKTPVYNGALAENSSLKAKPAAPRT QAKTEPQRSPAPLPALQPAPGVNPKVWLSRMIKAQALLAAQTARLARELGVSDAELRRLGRRHGMEVFHGTR

complement(798757..798948) PHAGE_Pseudo_JBD18_NC_027986: hypothetical protein; PP_00747; phage 4.13e-40 - NONE NONE NONE
MSQANPFIRPDKDYGAVSADDRLRALDSFNLEQCRAALSVPGLQKTVEKKLHSRIRQLNKEAR

complement(798950..799639) PHAGE_Pseudo_B3_NC_006548: hypothetical protein; PP_00748; phage 3.53e-168 - NONE NONE NONE
MDQDRILDKIKKCLEMAKGRGSNPNEAEIALRHAHKLMEAYNLEMGDVLASMAGETKVPAGSDGKPPAWRVSLAQVCCHAFGT HLIICTSYFESASFLFVGCAAAPELTGYAYQVLERQLQKARKDFLSTQKRCKRSTKVARGDAFAHGWIEAVYAKVDQFAGVDDNI ADAIQAYMAKHHADVGKFEMKRRKLKARDEVASEAGYAAGKRARLHQGIGHQAVARLTQGV

complement(799641..800264) PHAGE_Pseudo_B3_NC_006548: hypothetical protein; PP_00749; phage 3.29e-148 - NONE NONE NONE
MAEQPVHVPAGYRMDAKGRLVPEEMIKPIDLERDRLVQEIVAKGKALNKALLDFKLATFGDIEAFITLSAEQYQAKVGGKKGNASL VSFDGRYKVIRAMADNIAFDERLQAAKALIDECLHEWTEGARAEVITLINDAFRVDQAGNIRTGSVLALRRLQIDDERWQRAMQAI GEAVQVVSTKAYVRIQERVGDTDQYRSIPLDIAGV

complement(800257..800457) PHAGE_Pseudo_JBD25_NC_027992: hypothetical protein; PP_00750; phage 9.24e-40 - NONE NONE NONE
MADVLEIDCPACSTPYPEITAGSAAHDPSLIELVITCSNCGHTLNAFVSLAEMSVVPNPEEEPSHG

complement(800450..800983) PHAGE_Pseudo_JBD18_NC_027986: hypothetical protein; PP_00751; phage 2.43e-127 - NONE NONE NONE
MSVEAYIRGMAARGFSRSAAAAALGMHWVKFMDLLERMPDIEWGYPYKSFDRRRHAKNLKGYRFRDSEGRQRSVAALRAVNQ ARRHEYTVFGVTDSLSNLVKRFGCVAKSTVQKRLAKGMSIEQALTTPRSDHLSGLKRKPESHPWKRADRRGVINHRERQLKAKR DQRQAEERLHG

complement(800973..801653) PHAGE_Pseudo_JBD25_NC_027992: hypothetical protein; PP_00752; phage
1.26e-165 - NONE NONE NONE
MDSKLSASMAALRKVVKSPHPAMFWQETMGHVAVVLDHLQELISDGSTAPAIEVQPGDHSELRRIAVALKNPLLNGQEASDLMV
RYEALTMPDHIIALIDGLPQHVLDKQCRDDVARALGLCPNQERGFAWSYLLTSIKSCVKASEDTCRAQHSVPAGWKLVPIEPTPE
MLDARRDCEDGMDGYLVEDTEYYFPDRGAVRDFLACVYHGMLAAAPAPSKRSADDER

complement(801653..801937) PHAGE_Pseudo_JBD25_NC_027992: hypothetical protein; PP_00753; phage
9.19e-63 - NONE NONE NONE
MTVITHAYTPLMDVDSMSEEDCRLALKDVLQDGFAKDQQLVELKTGIHKLDRMLVKLIDLFIAGDFSKLHAELQSMAAYLQEQRAA
QKSARKVH

complement(801934..802275) PHAGE_Pseudo_JBD25_NC_027992: hypothetical protein; PP_00754; phage
3.24e-76 - NONE NONE NONE
MNVVPITGRLPEEQPKATHLPLCTVLTPELARCLEAVNSATRALRQAGIPIEQTSVLDRRLFIREEDSLRLHRRFRNAIRGIRQTTH
GMVTVHVVSLLGVDVAWTTPVKEQDQ

complement(802277..803443) PHAGE_Pseudo_B3_NC_006548: putative transposase B subunit; PP_00755; phage
0.0 - NONE NONE NONE
MLKLKEVLASLGKPQTDLARAVDLSPAAIAQLINHSQWPKSLDQQQLAWRITEYLMAQGAQFDTVRQAFDEVGPRRSNVGAPAT
PEDAQENEECEPMLMRKQVLLPATKKAFDIRRDPFDELHSADDIFINADIRYVREAMHQVAMHDGFLAVIGESGAGKSTLRRDLE
HRLEGSPVTVIQPYVLGMEDNDTKGKPLKSEHIAEAILAEIAPDQTPRNSSQARWAQLHKALKASHTAGSRHLLIIEEAHSLSTPTI
KHLKRYRELELGYTKLVSIILIGQPELLIKLSPRNGEVREVAQRIEIVELPPLTVGGLEQHLAFRFERVGKALSDVIDASGLQAVIERL
GGVKENKPSLLYPLAIGNLVKAAMNYAALVGEPRVTADVVREA

803085..803096 attL N/A
TCGAAGGGGTCG

complement(803443..805227) PHAGE_Pseudo_JBD18_NC_027986: integrase core domain protein; PP_00756; phage
0.0 - NONE NONE GO:0015074 - DNA integration [Evidence IEA];
MSAVITQALVDLERALRAAPRGQRVEIAQSTAQRLDMSLATLYRKLREVTADSKPRKRRSDAGTSALSREDALTISSALMESARR
NEKRLYSLEDAVEALRASKMIRADVVDEDTGEIRPLSISAISRALYSFGVHPQQLLQPAPVTELGSCHPNHVWQIDASLCVLYYLK
PGADEHGNGLRVMEHDQFYKNKPKNVARIASNRVWSYEITEHASGWIYLKYVMGAESGENLCDVLIDAMQERGGNDILHGVPKI
LMMDPGSANTSAMARNLCRALRIRVIVHKPGAARVTGQVENARNLIERKFEAGLRFQPVADLDELNAAAKTWRAWFNAAKKHSR
HGMTRSEAWMRIREHQLVKAPSVEVCRQLAIAEPESRKVTSKLRVSFQGTEYDVSVVPGVMNGEKLMITRNPWQSDAAQAITFD
QDGHEVFHVIPRIEKDNFGFDVRAPMIGEEFRPHAETPAQKARKEAARLAMGVDTDAEEQAARKAKAIPFGGRLKPYQHIEDAQL
PTFMPRKGSELQLDVTLPTVESKPLSHPAAAKILRARLDGVWSPESMLWLKSNYPDGVLEDQLDSIVEQLQAASSRPALRVVGG
NS

complement(805231..806205) PHAGE_Pseudo_JBD18_NC_027986: hypothetical protein; PP_00757; phage
0.0 - NONE NONE NONE
MARKASPVKVESIPEVNQQAYQAEAGALTMLGDIAQGMHEERDLVNQLLGQAQMAGAFEEFSRTVRTSKLAYVKENKLYRAIAG
KKSPNGSEFSGTWDEFCSLLGISVDKANMDIANLRTFGEEALESMSRMGIGYRELRQWRKLPDDARSALIEAAKQGNKDAVEYL
AEELIATHTKEKAALEKQVEDLRADNEALGERMARKSRELDETVHELEKTKRRIQTMKADEAEKELRQEATAIAFEAEADISGKLR
EAFSVMLDHAEKTGTDPRTFQAGLVRHLEKLLLQIREEFQLPDGEAPDDISEFGWIEQMGKSQPAGVAED

complement(806215..806529) PHAGE_Pseudo_JBD67_NC_042135: hypothetical protein; PP_00758; phage
2.37e-68 - NONE NONE NONE
MSTEKYRSEQVQRTLRVMLALAANEFRGLLLKEVAVAAECDASAALRALENLRIAGLADRSPHDDKRWLLGPRLVQVAFGFDEA
LRRGQDELNERRQRYTRLPN

complement(806526..806786) PHAGE_Pseudo_JBD67_NC_042135: hypothetical protein; PP_00759; phage
9.43e-53 - NONE NONE NONE
MSESINLQALLQRLDEQAYEQLCIEAARLAEENEYLRTELTRMEECAEGWCNEAQHLHQQLAEATGGQAAITQSGALVVIPMER
CA

complement(806779..807267) PHAGE_Pseudo_JBD18_NC_027986: hypothetical protein; PP_00760; phage
1.47e-117 - NONE NONE NONE
MKRRNWKHWVPRSPAEALDGCAQLAMQRYNRGIERLATDHLCQNNASTLYKWMGNGRLPLTMVLSLEKACGLPLITRYLAAAH
GKLLVDIPVGKACNANDLQQLQGVLHNATGALMAFYDGKQTAEQTLDAIRAGLESLAWHHGNVAQAETPQLDFGVADDE

807391..807615 hypothetical protein; PP_00761 N/A -
NONE NONE NONE
MTTPDHLVLDASMRSAFVALARRLAIDHGLDLQGLACDLETLADAQSGETWQMPHRDLAGVLRYVAERAQAGGS

complement(807900..808130) PHAGE_Pseudo_JBD18_NC_027986: BcepMu gp16 family phage-associated protein;
PP_00762; phage 8.55e-51 - NONE NONE NONE
MNVPYPLPTRTPYTGERVKELFRAAGITISAWAEANGYPRHQVYMVINGQFKGRRGTSHEIALKLGMKLSVEQLAA

808592..809095 PHAGE_Pseudo_JBD25_NC_027992: putative lipoprotein; PP_00763; phage 3.48e-
110 - NONE NONE NONE
MDFKVLLGAVMLVSLSVAGCSTKNYGRQPELTDFERQTMSCREIDLEQAKVQGFLTHVREESEFDGRSVLSFLGDFGIGNLMEK
DAAVDSANQRLTQLAGAKMQRGCTYAYEAEAPAQQPYAPPRAYAPDGPASASARSVDAQLDELNRMQLPYEEYQRRYREITG
Q

809247..809543 PHAGE_Pseudo_vB_PaeS_PM105_NC_028667: putative holin protein; PP_00764; phage 4.87e-63 - NONE NONE NONE
MSSPQPRRRRAPRMTSWTLVTLVLLIILAAIRPEQLQVVAYKLVLVTLGAVAGYWIDRSLFPYVARPHECSANLVVVGAWLRRGLI
VLACILGLTLGL

809546..809704 PHAGE_Pseudo_JBD18_NC_027986: hypothetical protein; PP_00765; phage 3.59e-30 - NONE NONE NONE
MGAPQIIWIVLAAVVLVTSYACDGLTNVISFKQRVFDVIAMTALVWWGGFFG

809701..810330 PHAGE_Pseudo_JBD25_NC_027992: transglycosylase SLT domain protein; PP_00766; phage 6.22e-149 - NONE NONE NONE
MKRLLTLGLLGLLSACQPAFATDRIPTAAEQYRRTLVRSAHAEWGLSAPIATFAAQVHQESRWRADARSPVGAQGLAQFMPGTA
EWIAGLYPTALGTNQPFNPGWALRALVTYDRWLYDRNQASSECDRWAFVLSAYNGGQGWVNRDRRLASASGADPLAWFDSV
ERFNAGRSAANFRENRNYPRLILLRYERIYLQWGDGVCGERYTL

810532..811155 PHAGE_Pseudo_JBD25_NC_027992: hypothetical protein; PP_00767; phage 1.02e-147 - NONE NONE NONE
MGILSLLRSNWFWIALIAVLYSVAVVIHGSASYDRGYATARAEGDAALLNLQLQHSNELAKIAEDNFLQFQQQVTRANQAEARFLS
AQDQFTALQQQLSERIAHVSTQYRPAPGASPVPAPRFVVTCGWLRDYNHALGADLPSPAACRTAASPQETAWPASGADAELLE
SGVSAADILAHARDYGKWSLTNLAQLNALLDVNDKETH

811155..811475 PHAGE_Pseudo_JBD25_NC_027992: PF10805 family protein; PP_00768; phage 2.06e-70 - NONE NONE NONE
MDLDFVLRAGQFVFTAAVGLYSLAAARRSSSKAEAEHLTNRLSSQDNRLLTLEQQMLHLPDSQQLSELAGDMKAMRAELSGLAK
ALDPLTRSVDRINDYLLSERRP

811472..811774 PHAGE_Pseudo_JBD25_NC_027992: hypothetical protein; PP_00769; phage 2.26e-67 - NONE NONE NONE
MTSNYSDFISQDRRLVILRILAEMPTYQANSSVLHAVLSQWGHDPSRDQVKGELRWLEEQQLVKIEDVSNGAVLVAKLTERGAD
VAAGRARVDGVKRPGA

811777..812325 PHAGE_Pseudo_JBD25_NC_027992: PF11985 family protein; PP_00770; phage 6.10e-124 - NONE NONE NONE
MGRKSSIDKLPPDVRSFIERSLRENRLTLDELIEQLQERFPGKEKPSRSAIGRYKVSFDEMTRRLREQQAMASLLVEELGENPDE
RAGALLVQSITTLTTHAAFAAQTEDEVDIEDVRKLARAAKDVLQARKASMEERRQIEREAREKLLQEQEQRLEEQRGSDGMSEQ
LENRIRGILLGKA

812327..814000 PHAGE_Pseudo_JBD25_NC_027992: hypothetical protein; PP_00771; phage 0.0 - NONE NONE NONE
MAMRATTAELGKRLTATSAPRKIDLAEEMELLGVDVPQEISEAQPANEPVFLPYQQRWFEDESQIMIAEKSRRTGLTWAEAGRN
VINAAKPRRRGGCNTFYVGSKQEMALEYIAACALFARAFNELAEADVYEQTFWDEGKKEEILTYMIRFPKTGRKIQALSSRPSNLR
GLQGDVVIDEAAFHESLEELLKAALALTMWGNKVRLISTHNGVDNPFNTYIQDAREGRKDYSIHRITLDDAIAEGLYKRICYVTGQA
WSPESEKAWRDGLYKNAPNIESAEEEYGCVPKKSGGAYLSRVLIEQAMVADHSIRIHRYEAPAGFESWTPELREAEVRTWCEEN
LLPELARLSDQNRHTFGEDFARRGDLTVFTPLAISPTLRKRVPFQVELRNLTYEAQRDIMRFICDRLPRLSGLAFDATGNGGYLAE
QAALKYGAGMVDQVQLNLAWYATWMPKLKGEFEAFNLEIPRHQTELDDLLSIKVEKGIPVIDKGRTKDLESASGKGKRHGDAAIS
LVMAVRASYMEGGEIAFTALPRHSRGFDNVQDHNNDIELPEPSAW

813994..815568 PHAGE_Pseudo_MP29_NC_011613: portal protein; PP_00772; phage 1.35e-153 - NONE NONE NONE
MVTMTRILGPDGQPLRLNEIREPQTAQLTSLHHEVAGHPSRGLTPSRLASLLDSAEQGDIVAQYELFEDMEEKDGHIHAEMSKRR
RAVAQLDWDIVPPDNATAKEKEAAAALYNLMQGLDDFEEVIFDTTDAIGKGFACQEFDGWQRVDGNWLPKAIIHRPQSWFQLPR
GVRQEIRLRGPSGGTPLQPFGWITHVHKSKSGYLERSALFRVLVWPYLFKNYSVGDLAEFLEIYGIPMRVGKYPTGATEKEKLTLL
RALAALGHNAAGIIPLGMELDFLNAAQGDPAAFQLMIEWCERTQSKAILGGTLTSQADGKTSTNALGNVHNGVRKDLRDADAKLL
AKTLSRDLVYPIAVLNGLVDSWARCPRLVFDVQEAEDLSAYATALPPLVKLGMQIPRSWAQQRLAIPEPAEGEEVLATVIEPVVPP
AQVPTRALGKAVATAETPPPKTADQQLDDALRPTTDRWIDQVRALVQSASSLDEIRDGLEQLLPDMTLEQYADAMAQALAAAAL
QGRVEILQEVAGGA

815558..816796 PHAGE_Rhizob_RR1_B_NC_021557: head morphogenesis protein; PP_00773; phage 8.27e-82 - NONE NONE NONE
MALRATSLPFAEQNQFFRRKLNLPTNAWTDIYTREHDYAFVVAGANRDDLVQDFRQAVEKAIADGTTLEEFRRDFDRIVAKYGW
SYRGGRNWRSRVIYETNMRSSYMAGRLEQLMAVREERPYWQYLHSDAVEHPRPKHESWNGLVLRWDDPWWQYHFPINAWG
CQCSVRALSEDDLRRMGKDGPDEAPPIVWQARTIGQNSPDGPRVVEVPEGIDPGFEYMPGQARLDTAVPQPRNGGPTPPAGL
PSTPASDPLPAPRPVPTNQLLDQDMLDADAIKRFLRPFGATLDKPAVFQDVVGQRVVVGREMFVSRAGGDLLVAESGMSKKWL
MLAAEALRRPAEIWVRLDWVESLKKAVVRRCYLASLQVSGEAAPVQVVVELDANGWAASAAVVQPGQQPLAPYRQGVRLYQE
T

816798..817373 PHAGE_Pseudo_JBD25_NC_027992: phage virion morphogenesis family protein; PP_00774; phage 7.76e-139 - GO:0044423 - virion component [Evidence IEA]; GO:0005198 - structural molecule activity [Evidence IEA]; GO:0016032 - viral process [Evidence IEA];
MAGVTLEYSSEKVLEALRAAADLMRSPAPMFRDMGEYMLIALDERFESQSAPDGTPWQALSPAYQRRKRKNQDKILVLDGYLK
NTIRYQASDDELAVGSNRAYAAIHQFGGEIQIAARSQQAYFRHDAKTNEVSPQFVNRRRANFSQWVSLGPYTIKIPARPWLGTSN
RDDDELLAIAQKHLDRALSGKSS

817584..818693 PHAGE_Pseudo_JBD25_NC_027992: Mu-like prophage I protein; PP_00775; phage 0.0 - NONE NONE NONE
MKTKPLLAAIALAACSFDIQAPTEGNLITLQVTPAGQFKPRDNREMKVPAWNIDAALAAAVVQRFAAKKTPPVLDYEHQTLWKEE
NGQPAPAAGFFRALEWREGQGLFAQVELTARAKQYITDGEYRYFSPVFLFDPVTGDVLDLQMGALTNNPAIDGMQALSERAAAT
FQLTIDPSNEESLVKPLLKAVLAALGLAENTTEEQAIAALSAHTTDLASMRKQLGLDDTAACSAMLAACTGLKAKAATAVDPAKHV
PVTVVDELKSEIAALTIRLGQRDEKELDAEIATALEDGRLHKSMEKWARELGKENRASLTAYLSAAQPIAALSGSQTRGQPPVPDE
KTGLTADELAVCTAMGITIEAFKAAKEA

818699..819103 PHAGE_Pseudo_JBD25_NC_027992: hypothetical protein; PP_00776; phage 2.35e-91 - NONE NONE NONE
MALTKDRNTPRRDGMQFNDPVAANAKIFAGSLVCLDASGNAVPGALSTTIAARGIAQEQVDNTGGAAGAKRIETRRGVFQLANS
ASADQITRADIGKECFIVDDQTVAKTSATDTRSVAGVVRDVDDGGVWVEI

819118..820014 PHAGE_Pseudo_JBD25_NC_027992: Mu-like prophage major head subunit gpT; PP_00777; phage 0.0 - NONE NONE NONE
MIINQQNLRNLFIGYRAAFQNAFAGVQPDFNQFVLTVTSGNASEQYGWLGNSTAFREWLGDRVIQNLGVHDYTIKNKTFENTVG
VPRESIEDDSYGLFTPLMGQLGQDSAMHPAELVYALLSGGFTQTCYDGQYFFDTDHPVTSAAGNEVSVSNFQGGSGTPWFLLD
TTRIMKPLILQKRKDYNFVTMDAEKDENVFMRKEYVYGVDARLNAGFGLWQLAYSSREALDASSFNDVYAAMQSLRGDKGKRL
GIRPKLLVVPPSLRSQALEVVKAERNAAGATNINRDVVDVLVTPWLAA

820027..820461 PHAGE_Pseudo_JBD25_NC_027992: hypothetical protein; PP_00778; phage 7.45e-68 - NONE NONE NONE
MAGKKRTADQPEERSGVAVSADTGLSTGDFTSPPGVSAGPQVVEQPGETIAQDAQLGQQSGDAAPSPDAALQPMASAKGAAG
EDEVEALFVRSVPDSFRRCGHRFTREGHGIALSLLSDAQVDALLNDPNLVVEHCSFALKDVS

820464..820979 PHAGE_Pseudo_JBD25_NC_027992: PF07030 family protein; PP_00779; phage 2.24e-122 - NONE NONE NONE
MDYITLVHLAERPGAKELAQVATAQHLKIVDSALLDAALRGGDLSAWTPEQVAGVDLVLERITEAMTEAESIVNGYLAKRGYGLPL
SPVPGLVTGWVRDIGRYLLHKDRISDDKDAILRNYKDALKFLQMVADGTFSLGAEDPIANNPMLADVRFDADENVFNRQQLRSFR

820976..821428 PHAGE_Pseudo_JBD25_NC_027992: hypothetical protein; PP_00780; phage 1.05e-103 - NONE NONE NONE
MSNAPFDHRLVIERLTATVPALRLIGTAADFGAVKALRDYPTPAAYVLLAEESGEPRPTGNSGGPARQRVGALFGVVLAVRSYRY
DQLADAADDLQSILDQVRGAMVGWVPSLPLARGTQFVIGKVLDSDDTTLLWGEIYSTQHAIGRDP

821425..821628 PHAGE_Pseudo_JBD25_NC_027992: hypothetical protein; PP_00781; phage 5.91e-41 - NONE NONE NONE
MSTKQVADTAEVKREKVILIADHTHGEQKCKTGDEISVTSIEKEWLIRHKRIAAPADQAAAAGKAKE

821635..822375 PHAGE_Pseudo_vB_PaeS_PM105_NC_028667: putative tail sheath protein; PP_00782; phage 1.23e-92 - NONE NONE NONE
MSLISLQGKIWMAERSAQGKALKQTWVGNAPTCELQLATETTNKTESFSGNRLQYGQLDRGKTATINLALDEWLLPNLILGLYAQ
QVAIPGVTVTGEALPTPIAVGDVFRLAKPFISDLVLTQSSTPLVAGTDYKIESATAGLIEFLTAQASAVSAAYESEEAVALTMFTQRP
PERWLFLDGIDTETGKSVLVDLFRCKFNPVGTLAMIHEEYGNLPLTGSVLYDPLNAGDPMLGGYGRYIEKKAA

822378..822860 PHAGE_Pseudo_JBD25_NC_027992: hypothetical protein; PP_00783; phage 9.42e-115 - NONE NONE NONE
MARKVTRKKSATGAEDLSVIHPDRTIIIAGRDVVMREYGFFESLELLPLLEPILVDLEEQAKANAPWPGIEAVPSFLGNHFSVLVHLI
AKAASVDMEWMRGLGADDGYELVWWWWIVNGPFCKRCAEKRLLTAQAIAEQARKRDAGQTPSTTSSPPVTAP

complement(822815..822997) PHAGE_Pseudo_JBD25_NC_027992: hypothetical protein; PP_00784; phage 5.15e-37 - NONE NONE NONE
MTFSVLTARKSEVDVDHPFSAFAPMSSLHRVELLQDLPTSHLANVGYGAVTGGDEVVDGV

823114..826755 PHAGE_Pseudo_B3_NC_006548: tail length tape measure protein; PP_00785; phage 0.0 - NONE NONE NONE
MANRDLEIALRLRADMKDGQAAVEALAAAIRDVGSKASEAGTGLQKVGATGAVDQAQGAIDKLGHSLDGVSAKASEAGKGLQKV
GTTGGVDQAQAPIDKLGHSLDGVSDKASEASKDLQKVGATGAVDQAQASVDRLGQSLDSTGKRAGDASRQITQVGESAEQQA
ARIKAMVAASLQQKSAQDEAADSTQRLNTAVQAGNTAWRDSAQAQSNAMNTFHNAERARVQQVAAEKRAAEAAAAAAAETSR
QEAAARKLLGAIDPTYRSLAQLADQERQLTEHFRAGRIEATAYASALDRIRARRDVLNGIGNDARTSTVALNSMGAAIRRVQGLLV
AGVAGYGVASFSKEVVNTNLQWQQALYTMEAATGSAAKARQELEYVREVSERLGLELLNTSQAYSRLVAAAKETPELGGSLRTI
FEGVASATTALHLTRQETNGILLALEQMVSKGKVQTQELVLQLGQRVPGAFSLAAKALDTNTKQLSEWLEKGMIPAAEFLPRFGA
ALQEAYGPASQKAATGLQAELNRLENAFTDLKIQAGESGFIDTFTQAVRDLRDVLKDPAVVEGLNLLIKGLGTAIGYAAKGAAGVV
NVTKFVAEEIAARVNGPAADDVPRLDDAIARETEYMARVQSALDDAYEKNDQKRIQRYEEALSKAQAQIQQWQDQRDAVLNGA
GQVAALPATTVTGTGPTTKTPFTPSGGEDKAAARLAKQNEDWVKQLEKEAATYGKGRAALREYELDQRNLTGALEARARAAWA
TLDAAEKQKKADEQAKKDATTLKQLNLDYLRATGQTVEAAGAEIEKKYGDLQKRLLATGDTEGAGLVSKLMGIEKAKAELQQLQD
QVDRIFGEQSRQESSIQAAQQAGLVSELAARQQLLDLHRSTADEVEQLVPRMEELAKATGDPAAIERVKDLRQQLENTRVAADQ
LTLALRSGIENGMQDALRGLADGTLSLQEAAVSFLQAVSRSLADVAAQQLAQKATAGLMSPFGQGEQDTSMVTGAAAVTSAAG
ALAAAGGTLVTGAAALQSAAGSLALANGVKGAGVAASGAGAAGAAGAAAGGSGWWSSITSMFGFASGGHIKGPGTGTSDSIPIL
ASNDEFMTRAAVVRQPGALAFLEQFNRYGMAALSAWANPVRHATGGLMGTPAPAMPAPGLAASRLQEPAKNLSATLKNNQNF
YLVDDPSRIGDVMAGRYGDEAMVLHISRDPQKFRQLLGIN

826755..827711 PHAGE_Pseudo_JBD67_NC_042135: hypothetical protein; PP_00786; phage 0.0 - NONE NONE NONE

MATEFGTAANHADLVERLVQFLTANPTLVAAGQAYEKTFDNTIPASGTAIAIRQVTLRAPGLGGDDSIFMGVQSYGDTALDYYNV
RLMGGTAFNPGALPPGGDFWTAFVNYSPRVQLLAWNQPMPYWFFANGRRFWLVVKVSTIYESAGAGFILPPCPPSQFPYPLAV
VGSYRGDVATRWSDVSDRHRGISSPYERSCYLRDPAGRWLGFTVAGGSANESDYSNRTLLPLGCGRYAGSSEAVVNQLRDSF
GKFPLKALSLVTRETEGRRYLGDFDGAWYVPTLNSGAEDVITENGVDHVVFQTAWRSGNPWLFAIRAD

827714..828637 PHAGE_Pseudo_vB_PaeS_PM105_NC_028667: hypothetical protein; PP_00787; phage
0.0 - NONE NONE NONE
MAYFTGTANNPSDLLGKLRTHAETLGWVTDRASASEWLCHNADGYWSFNAGSNQWQLAGNTGFDNGLAWNAQPGSSVQNN
PYSSKEATIAQLSAGPFTRYHLFATAAYLHLHVEIAAGQFRPVMIGSLNKRGVAYTGGQYVCGSFIYSPGQALTNNWSSHPFDGY
HIRYSGGGSVLRLDSLDGSPSPDWLPFDYTTNVSRRVVGPGRGNYSSQYHPDVGLIDASANELNSSTNVVPCAIYAFGAQQRSR
YIGEAPDFGLCRMDFLAPGDSITIGTDTWRVFPLLQRGTASDFGNTSALVGYAFRVVE

828640..830346 PHAGE_Pseudo_JBD18_NC_027986: hypothetical protein; PP_00788; phage 0.0
- NONE NONE NONE
MATFPGFQVPRPVEAVVAGITPNISALFLNQDITLGSASASIWAGSYAAHQPVDVIHSAYSAVHQSDLAENFYNRIWLIPGRLDLG
NVVSVQERPVSVWNAHFTPRTLSQIDREDADGISLAGQPSPPLPFAALQERIWTVAVSTDGPPVVDARIVWQLQDEQPLILVITG
NRITAWPFAPDWADGVQESLEWLTELLTSTSGVEQRRSLRLSPRRSFEAEFYAEGRERVLLDLSLAGWGGRIWALPVWPDIQLL
ASVTAAGALTVECDTRWRDFRAGGLALLRGESAFEYEVVEIQALAASAIQLARPVQRRWPAGSRLYPIRTAQLTEQPALTRLTDT
LYSAQARFLVMDSSDWPEVMPSTTYRGWPVLEQRPEESEDLSVSYQRLIDVLDNETGLPQFSDQAGIGFPVHGFRWQTEGREE
HAALRSLLYALRGRQKAIWIPTHAADLVLVDTVAATSSVLDVELVGLARFFRADAPGRRDIRIELYGGQVFHRRILDVSELNVDVER
MAIDSALGTVVRPSDVARISFMTLCRQDSDSVQITHETDTDGISTASTVFRGVRDELQ

830333..831151 PHAGE_Pseudo_JBD18_NC_027986: TIGR02218 family protein; PP_00789; phage 0.0
- NONE NONE NONE
MSFSDRERSLADGQAISLYDFRLGPIRWTYTTANRDIEFNNMTFRARPVSDDGRRMTGQVSADIMTVTGPSDLEVAQLYRGARP
SKAPTLTVWDIHWNEPQGLVVWMGRIDEVNWPADSRVQIKCRLLGTEPRTSISLAWGRECPYTVFDHNCRADREQYRVPFTVE
LRDGNSVTGAGNAIGGYPDAWFRGGYVEWDSGQGVIEQRGIQLHTGNRLVLVGGTSLLAPGTRAVAFPGCDQLIQTCNDKFDN
TPNCGAVPFLPGKSPFDGDPWW

831161..831391 PHAGE_Pseudo_B3_NC_006548: hypothetical protein; PP_00790; phage 1.60e-49
- NONE NONE NONE
MWVQIAILVASYLISSATSAKAPKPKPEALTSEDLPQTEDGTGHYVIFGDVWIEDWIVLGTGHERMQAVKSKGSKK

831371..831382 attR N/A
TCGAAGGGGTCG

831605..833815 PHAGE_Pseudo_B3_NC_006548: hypothetical protein; PP_00791; phage 0.0
- NONE NONE NONE
MGGRSKAQTVGFRYLMGILMGFARGPLDELVEIKAGDRTAWKGSVKSNQTIQINAGELFGGDKAEGGIVGPLDVMFGAPDQPV
NPRLAAMVGGLVPAFRGVTTAFFDGQLCAMNKYPKAWMSRWRRALNGWDGGTWYPEKAVISLAGDQVKAMNPAHILFECQTN
RDWGRGKDRGLLDQASYRKAADTLFAEGFGLCLKFRVADELDNFEQTVLDHIGATQFLSRSTGLWTLRLIRDDYDVATLPVFDE
DSGLLGIDDDSITSLDGTANQFVVVWHDPITNTDRRARAKNAGAIRAAGGVITTTKEYPGLPTGELAGRVAARDCNVSTSAIRKLQ
VRLDRRAYALNPGDVFCVRSRKRGIELIVLRAGKIDYGTLTKGTIAITALEDVFGLPAAGTSAVQPPNWTPPDRTPRVIATRRLIEA
PYRDLAAALSDADLAQLQPETGVLAVIGMRPSGLQMNYALLSRVGSAPFEERTSGDFCPVATISADIGRGLSSVSVTLVQGVDLD
LVEVGSAAMIDDEIFRVDAINAAAGTAVLARGCVDTLPAPHEAGALIWFYEDWTAEDTREYVTGETVQVKLLSRTSSATLAENLAP
VDSLRMNQRQARPYAPGRVLVCGVAYPTKTYGVLTVSWAHRNRLLQADQLVDSSASSISLEAGTTYTLSIYSGTSLKKSYTGLTG
TTWTYPVEDDIAHGLLPVLHIVLFSVRDGLQSWQQHDITIERHGLGFRLGEELGGVAQ

833812..834960 PHAGE_Pseudo_JBD30_NC_020198: hypothetical protein; PP_00792; phage 0.0
- NONE NONE NONE
MTLYMGPNTGLLINGLPGEGHYSDLIRMWRWDDFLRQPVVKGRVASLPTSGQAEGDTYIFTGSGANQNRIARWWATGATTPIW
EYMPPRLGWRVQVANETTPSGQVKTYEYSGSAWTELVGGMSDAPSDGKAYARESGAWTELGSAAKSALNVLPFMNLMPDMG
RFAGTAANPLNTMFTTSWTPSTFINGWNGAALADGGKFSFDNSTNGGAGPALNARVQALLTAMGRTWTSVSRYGVEFFTTVLT
AGSQTTTGSAGADGVTRYLCCSNGSKTVFNAGAWATVVMWLRVESGSAHISSAPYTTHRLWINGAVAAPGVVLPAGQWVHLR
FSMQSYNGYDNACPYIYASAGAQIAFACPAWFGGLVDPGIHVAPILTINGASA

834957..835247 PHAGE_Pseudo_H70_NC_027384: hypothetical protein; PP_00793; phage 5.16e-59
- NONE NONE NONE
MTMKRVLLKGEFFAEWDGTLDEAAALAGVPVGDLAFHPDDVLAEVKELRRQAYRTESDPLRLEAEFDAIAAGSEPDLAAWVAAV
QAIKARYPLPSE

835386..835577 PHAGE_Pseudo_B3_NC_006548: Com translational regulator; PP_00794; phage 1.05e-
20 - NONE NONE NONE
MKDIRCGGCNRLLARAGQFDQIQIKCPRCGTLNHLKAESLLIAPLSAPCRQEASCPPNPSSPG

835547..836341 PHAGE_Pseudo_B3_NC_006548: Dam modification methylase; PP_00795; phage 0.0
- NONE NONE NONE
MSAQPIIPWIGGKRRLADRIFQLFPRHSCYVEPFAGGAALFFLRPVPAEVEVLNDVNGDLVNLYRVVQHHLEEFVRQFKWALSSR
QVFKWLQETRVETLTDIQRAARFYYLQQSAFGGRVDGQSYGTATTQPPGLNLLRIEEALSAAHLRLSNTYIEHLTWQDCMKRYD
REHTLFYMDPPYWETEGYGVPFGFEQYLEMAEMLKRLKGKAIISLNDHPDIRRCFTDYHIEATDIRYTVGGGKGSDAREVLIFSW
DIQAEPAGLF

836460..837266 PHAGE_Staphy_SPbeta_like_NC_029119: bifunctional AAC/APH; PP_00796; phage
1.75e-06 - NONE NONE GO:0046677 - response to antibiotic [Evidence

IEA];
MHDAATSMPPQAPATWADYLAGYRWRGQGEGCSAATVHRLEAARRPTLFVKQEVLSAHAELPAEIARLRWLHGAGIDCPQVLN
ETQSDGRQWLLMSAVPGDTLSALAQRGELEPERLVRLVAAALRRLHDLDPAACPFDHRLERRLDTVRQRVEAGLVDEADFDDD
HRGRSATELYRLLLDRRPAVEDLVVAHGDACLPNLLAEGRRFSGFIDCGRLGVADRHQDLALAARDIEAELGAAWAEAFLVEYG
GDIDGERLAYFRLLDEFF

#### region 3 ####

1337187..1337212 attL N/A
CAGGCTTTGATGCCGTAGAGAACGTA

complement(1337285..1338388) PHAGE_Pseudo_phi2_NC_030931: integrase family site-specific recombinase; PP_01259; phage 0.0 - NONE GO:0003677 - DNA binding [Evidence IEA]; GO:0008907 - integrase activity [Evidence IEA]; GO:0015074 - DNA integration [Evidence IEA];
MAPRPRKEGSKDLPPNLYKKTDSRSGVTYYAYRDPVSGRMFGLGKDKARAIREAIEANHTEALQPTIADRLNSEPSRPPRLFDD
WLIEYEKIYAERGLAAASVRNTRMRLKRLRARFGTMDIRDIGTIDVAGYFSEMAKEGKAQMARAMRSLLRDVFMESMAAGWTDK
NPVEVTKAARVKIKRERLTLETWRLIYAEAKQPWLKRAMELAVITGQRREDLAAMQFKDEQDGYLQVVQSKTGMRLRISTSIGLA
VLGLDLASVIKSCRGRVLSRYMIHHHRTISRAKAGQPIMLDTISAAFADARDRAAKKHGLDFGASPPSFHEMRSLAARLHEEEGR
DAQRLLGHRSAKMTDLYRDSRGAEWIDVA

complement(1338369..1338611) PHAGE_Entero_mEp235_NC_019708: excisionase; PP_01260; phage 8.71e-08 - NONE GO:0003677 - DNA binding [Evidence IEA]; GO:0006310 - DNA recombination [Evidence IEA];
MKLVTLEEWAAEHFRTPPSINTLRRWARDGCIIPTPVKHGRSYYVSPDAEYSSQEPAKRSAPGDSLISRIKSARHGTKAA

complement(1338693..1338815) hypothetical protein; PP_01261 N/A - NONE NONE NONE
MKKALSRLAAVAVIGASLVALHALIELAPAFAALQWGCSF

complement(1338831..1339121) PHAGE_Pseudo_phi2_NC_030931: hypothetical protein; PP_01262; phage 3.03e-64 - NONE NONE NONE
MRTKTFRPPRRHELAGLRYYRTASAYNWLGITMAHPTRAIQLLLEQCEPDVLSPMFEIEIDAILRQADEYAKTGQVLEREQLREML
MHLISKAAGD

complement(1339193..1339513) PHAGE_Pseudo_vB_PaeM_PAO1_Ab03_NC_026587: hypothetical protein; PP_01263; phage 1.50e-22 - NONE NONE NONE
MAEELKPCPFCGEEAAFVELEDGGIVAVCASKGCVASGVARYACGDDPRPLIAETWNTRAVPAGHVVVSEALLRRLARPADRYD
EVFTLDRHEAAEELRALLSEQA

complement(1339506..1339706) PHAGE_Pseudo_YMC11/02/R656_NC_028657: hypothetical protein; PP_01264; phage 3.52e-43 - NONE NONE NONE
MLHEPEEYRLFSLWMLVFMAIGWFGGWIHAHYTVAEECRKLGKFYVGKTVFECKAITEEDKENGNG

complement(1339696..1340358) PHAGE_Pseudo_D3_NC_002484: hypothetical protein; PP_01265; phage 2.17e-60 - NONE NONE NONE
MSEVKRFDHVNHAHVDDCEHIDNPEGAWVKASDYDALAAEAQALREEVARAEQHRNDQADLIVSLRTEVAALRMARDDLKLER
DLARQNFCDEQAANYQLQAHLKACLGELSELRARVVVLPSVDNVMNIVMRYQWNEKTNVTGTTNWAANLGMRVVEVVKRLNG
KTVSEGLLRRISDLFPADMGDDGSGRAGWFPHVRETVAELRALLNQDKENGDHAA

complement(1340351..1340632) PHAGE_Pseudo_JBD44_NC_030929: hypothetical protein; PP_01266; phage 2.75e-63 - NONE NONE NONE
MAVYVDDMNATFGRMKMCHMLADTTEELLAMADKIGVQRKWIQHAGTIKEHFDICLSKKSAALAAGAVAITYPDGVAEIMKKRRA
ASKEVGHE

complement(1340632..1341348) PHAGE_Pseudo_D3_NC_002484: hypothetical protein; PP_01267; phage 4.94e-46 - NONE NONE NONE
MNDYKSAFNAAAADISAIIALLGFTKYPGVDPVLRAIADLILEKAENQVLREERDAANKRADTLEHRMMGMMTRHFAEAWRNRDT
QKALDGYLSAGIAQLEQERDAAQARVAELTATRNRYGLDAHYFNKNLQRILRDFESFTPDELARSLIILAKVADEKVVAQAQHSVP
EGWMLVECGIWTQEQVDEMQKTVARFRNSEFVDDRALAMAVADAGGQCKAPEISLAELLAAAPGKEGL

complement(1341341..1341724) hypothetical protein; PP_01268 N/A - NONE NONE NONE
MKQSEFRAELVKIMPGYNWTVHASRSSEKLLVATGIQSSGSNRLSTLRVERRDDYAGSGKPRYEVSSAGYGTRSPWLHTAQDK
TLVRALRSLQEHYEGNARKYSIHASDLQRGRIATPPTPGASTDE

complement(1341736..1343154) PHAGE_Pseudo_phi297_NC_016762: hypothetical protein; PP_01269; phage 6.17e-35 - NONE NONE NONE
MTKANECTCPSGDGSLRHPCPAHIGPFDKLVGSPVHNESEPDLPPLDEHLAQVFETGMALRESVEQAGGDERAAFEAWWQSA
EVLKCKRDVAMEAWQARAALAQPSQAQAEQATADDYEEVLADHRRLVRELDVLLNGEEGAAKQASLCDLVGQVSAIVRERRVP
LLSRSQAEDAPVIGCLCGMPMTEGHHSPEGCTSLEEFAPQAQAEQAEAERPEVVAWRYGSSGGIVSDKACLDEWKSGGEYQS
LMTVAQHERIVGELRAENERLANDRDRANQFANGAALEINAALARVAELEHVLRVVVNAADHGSWPTTVMHGIEKVREVLAGSA

PPAKAQHSVPEGWKLVPSDKGCVTSSMKAECIGEFSFYIRAACAECLDVGTDSDCHVCGGDIEYDQKIDVPWDTCKEIYKTMLA
AAPGKAQHSVPEVSGIGRDFAYPRSVVLYLRTEPTDDDLRAIHDGLRSLAAAPQPVA

complement(1343151..1343360) PHAGE_Pseudo_PMG1_NC_016765: hypothetical protein; PP_01270; phage
6.69e-37 - NONE NONE NONE
MPFDESPAVRRINALYPSNAPARYLHIPTGIHWVVIDSLGEVIQLENIERRRRLITVSDLETEAWRKLP

complement(1343585..1344346) PHAGE_Burkho_BcepC6B_NC_005887: putative methyl transferase protein; PP_01271;
phage 1.78e-73 - NONE GO:0008168 - methyltransferase activity [Evidence IEA];
NONE
MPGAYYNEFDPYAAQWLRNLIAAGHIAPGDVDERSIEDVHPDDLKHYTQCHFFAGIGVWSLALRRSGWPDDRPVWTGSCPCQP
YSKAGKRLGFADPRHLWPSWSHLIRERRPAELFGEQSPEALVHGWFDLVLGDLEEAGYAAGAIHFAAASCGEPILRKRVYFAAK
HLGEGAQGQQSRRSPCQAGPRRWRGEADLRAIADSPLQPGDRWPQPIVRSMDHGYSGRMGALHAIGNALNAEAATQFIAADL
DATS

complement(1344429..1345046) PHAGE_Pseudo_PMG1_NC_016765: hypothetical protein; PP_01272; phage
2.21e-42 - NONE NONE NONE
MSAEKPRERPILFNDQMVRAILEGRKTVTRRAVKGLQIPTEDKTTPHEGLRWSALGQRHLRYGFNVFGSTEEECAHELARCGVC
PFGKPGDRLWVRETFADIGCRLTFRADLEDGAHCSVTRWTPSLHMPRWASRILLEITAVRVERLQQITIGEICKEGLARSMYEFIP
VTTAFDAFAELWNSTGGDWDANPWVWVIEFKRVTP

complement(1345076..1345612) PHAGE_Pseudo_MD8_NC_031091: hypothetical protein; PP_01273; phage
1.65e-124 - NONE NONE NONE
MMRRVYLSGPMTGIPDFNYPAFNAEERRIRALGYIVENPAVNMVYRGAPWETFMRDGIKRLMDCDILALLPGWERSRGANIERN
LAITLGMHVVDAEALPAPDFVCKCRAIQFTCCSVPSDNDPFVCRRLADMPAYLSPEDQLANARQALEKIAALTDVSTGGIGMDVL
KIAKQALSN

complement(1345609..1345986) hypothetical protein; PP_01274 N/A -
NONE NONE NONE
MSTFAVFGMTRDVALAMANKEVKSVRKTPLGDEHVPMSEWLAAVERKADNIMTGTKVVQLSQLLDTPDFCQQFIDLARKTLECR
DMQIRARVQLWNDDGTPVLTKKRKHKVEWQQFGHQPGRAAA

complement(1346051..1346353) PHAGE_Pseudo_phi2_NC_030931: hypothetical protein; PP_01275; phage
8.80e-64 - NONE NONE NONE
MAKTNAQRQREKRQRQREAGIPERKLPSPPAIDAAFERLQAVGDFEDWREAFSTLLLNASALPDADLLPLLVVSRHEYTPSENVS
RQLLAAELSVADDEQ

complement(1346353..1346667) PHAGE_Pseudo_phi2_NC_030931: hypothetical protein; PP_01276; phage
1.38e-44 - NONE NONE NONE
MSLPINALKDDELLHYSQFDSGAADELARRLATGDLHIVDELSELEEYARELEEGKEEADDDLEVERAKCRDAIAVLEATVQFKPK
TVDDALHAIRSAIEILEG

complement(1347102..1347350) PHAGE_Pseudo_MD8_NC_031091: hypothetical protein; PP_01277; phage
1.88e-48 - NONE NONE NONE
MLKHQEQTEVLAGLPSQTALARLAFVQRLMAPAVEEPYQVVPQGRGFFHIVETATGAVRGFRRNHNEACAYAEHLKRQQAAK

complement(1347361..1347744) PHAGE_Pseudo_phi2_NC_030931: LuxR family transcriptional regulator; PP_01278;
phage 4.43e-82 - NONE NONE GO:0006355 - regulation of DNA-
templated transcription [Evidence IEA];
MQAIQCGGWIGRQGLGLAPRELEATAWSASELTAKEVARRMGIAPGTVEKRLDDAKFKLGVRSVRGLVLEAFRRGIISPAVVLLA
FLVAGHPLIDDDHMNRNRRPSNERRITEARTVRRIDEITINA

complement(1348055..1348921) PHAGE_Pseudo_phi2_NC_030931: ParB-like partition protein; PP_01279; phage
0.0 - NONE NONE NONE
MAAKSFKQMIKDGDLKRADAMKARLEDLHEEPGFNLRAEGEDLEQSIADLADYLHQGGIVPALEVRPREEGGMWVVDGHRRRR
AYLKLDAEGRLPRDPNGEFWVPIVAFAGNDAERVLRVITSQEGRKLSPLELAHGYKRLIAFGWTVEQIAQKMGRTRQHVDQVLV
VGNANTDVQQLISSGAVAATTAARIVRKHGEKAGQVLGQQLAKVIAAGGTKVTPRAVAEPTVPRAILDDLLKVTTDIVDAFPTALR
AGLAEGPESITLTTRSAWVERLMDLVAQAKESLQG

complement(1349187..1349408) hypothetical protein; PP_01280 N/A -
NONE NONE NONE
MRVHEKFSKKGARPLECVQPTVRTIAGAVHPVAVEFFQTSDSCDGRTLTAFMTPQEAMKLALHLLHVVQGAMR

complement(1349613..1349891) hypothetical protein; PP_01281 N/A -
NONE NONE NONE
MNQISIVGYESDCNCEHCGRALKHGVRLSDGRLVGATCLDKKLTKPRQYKGKSFRFGAEHIIKIAKVVQFYSPSNWARFGVSASS
TTFEGIA

complement(1349951..1350118) hypothetical protein; PP_01282 N/A -
NONE NONE NONE
MNAYKAGDKACYLGRARATVLGKTSRGYRIEYWGQGARDGELIRATVPARDLMPI

complement(1350633..1350839)	PHAGE_Pseudo_phi2_NC_030931: hypothetical protein; PP_01283; phage
7.46e-44	-	NONE	NONE	NONE
MAQFNVDAHLSNGKRLDWIALPEGNETPDDVLIKVRQAAMKKFGDLIWFNRWDHVVASNGYITVRMHA

complement(1351412..1352161)	PHAGE_Entero_DE3_NC_042057: repressor protein C2; PP_01284; phage
2.27e-28	-	NONE	NONE	NONE
MQTIRAAFIARLKEAASDAGFQEWGLGARLAKITKRTPKAVSKWMNLESMPERDAMLSIADAFGVRVDWLEHGQGEKNSRYMT
SNRTEDAVRNLVAERAGDYGNVQPTAQPSRKKKGYPLISWVAAGAWAESHDNFQPGDAEEWIESEAKAGENGYWLEVHGDS
MLPSFPEGTRILVQPEGFDLVSGKFYVALLYEPGKQRETTFKQYVRDAGREYLMPLNKDYKPLQVTENVRVIGRVIDLKPPKSLL

1352651..1353364	PHAGE_Pseudo_MD8_NC_031091: DNA-binding protein; PP_01285; phage	3.56e-
103	-	NONE	GO:0003677 - DNA binding [Evidence IEA];	NONE
MSQVAVIQQGPVLAMSSREIAALVESRHDNVKRTIERLGEKGVIRFTPSEETSHAGAGARPVSVYLVDKRDSFVVVAQLSPEFTA
RLVDRWQELESQLAHGVPAVPTNLADALRLAADQVEKNQALQLVISEQAPKVQALERLSGAAGTMCITDAAKHLKINPARLFDWL
QQNRWIYRRSGSARWIGYQPRIQDGWIMHKVTVLGRDDQGDERAASQVRITAKGLSVLARKIEEGKL

1353429..1354190	PHAGE_Pseudo_phi2_NC_030931: hypothetical protein; PP_01286; phage	5.39e-
148	-	NONE	GO:0003677 - DNA binding [Evidence IEA];	NONE
MSTIIMSACWPLQGLTPAQKAVLISLADNANDEGVCWPSVAKIAERTCLSERAVQQAIKVLNECKALSIEARQGRSTMFTVTPAAF
APPQKVHPRRKCTPAASAPTPADAAPPPPQEMHPTPADAAPRTVIEPTREPSGEPSPLPTRSGPAAGEALQEACRSVWAAYRA
AYEARWSVQPVRNAKVNSQVKQLVAALGAEAPAVAAFFVGLDDKFLVDSCHEFGLLLAKAGAYRTKWATAGSAPSTDWTDQV
QL

1354187..1354870	PHAGE_Pseudo_MD8_NC_031091: hypothetical protein; PP_01287; phage	2.85e-
160	-	NONE	NONE	GO:0006270 - DNA replication initiation [Evidence
IEA];
MTRRQFEPQSVGAVLAHVNQGAGLRPLSQPAVKVDPQTRGEVDRLFLRIKAICPGWRSSWPSDEVENAAKAEWLAEIVRQQVA
RREQLQAGVRALSAQARPLVPSAGQFCAWCWAPEVFGLPSLDDAYREALANTHPAMVGAAKWSCPAVYWAAAGAGFSRLQA
LARKDGLAALEISYRQIIKKLARGEALGKAPEGEVTHQKARTQSVGIAALAQLRKQLKGGDRS

1354867..1355073	PHAGE_Pseudo_MD8_NC_031091: ninG protein; PP_01288; phage	4.60e-42
-	NONE	NONE	NONE
MKWSVLNDYLMVSDTQPPYKVCKLLVAGEAHYRASVQGEFICTPVATAKEACGVCERHHQITYPREVA

1355070..1355651	PHAGE_Pseudo_phi2_NC_030931: NinG family protein; PP_01289; phage	1.43e-
138	-	NONE	NONE	NONE
MTLSARKPRPKKCAVSTCRAPFVPVKSFQTWCSPECGIVIARQKQEKERKSIQQRERREVKVRKEKLKSRADHLREAQAAFNEFI
RWRDWDRPCISCGRFHDGQYHAGHYRSVGSHPELRFDEDNVHKQCAPCNNHKSGDVVNYRINLVAKIGAAAVARLEGPHDAR
KWTVEEIKAIKALYRTKARDAKRAAA

1355648..1355944	PHAGE_Pseudo_MD8_NC_031091: hypothetical protein; PP_01290; phage	6.61e-
55	-	NONE	NONE	NONE
MKKHGPDLTNKPRHLVPCPACNGHGQHRGVFYDIDCDACGAAGFVDGATGLALEQRDAVVQLRMWVKRLLEEQRRQASRLAR
EENNQRGAGGSHFRGD

1355946..1356320	PHAGE_Pseudo_vB_PaeP_Tr60_Ab31_NC_023575: Putative antitermination Q protein;
PP_01291; phage	5.32e-14	-	NONE	GO:0003677 - DNA binding [Evidence IEA];
GO:0060567 - negative regulation of termination of DNA-templated transcription [Evidence IEA];
MNIKALEFLMEQYGLWVWSDNGTPRGSSPMLALMKRNPANEKRFAAVIPCISDDRALQVDRFLARLYDEDPDAIRSLILYFIHGM
SYRDIQDRMGISYADARMLVRAGLSALLACFVMEDKKAA

1356590..1356922	PHAGE_Pseudo_MD8_NC_031091: holin; PP_01292; phage	1.11e-73
-	NONE	NONE	GO:0019835 - cytolysis [Evidence IEA];
MKMPDKPDTWAALFAWLSQHAPIIYASLLSWAMAMARIIYGGGTRRQALLEGALCGGLALTIISGFEFFGVPQSMATFIGGWIGFL
GVEKIRDLADRYAGIKLPRRGSGE

1356919..1357536	PHAGE_Pseudo_MD8_NC_031091: glycoside hydrolase family 19; PP_01293; phage
1.67e-133	-	NONE	NONE	NONE
MKITADQLDRATGCGAATASTWVEHINGAMARFEINTPERVAMFLAQVGHESQSLKRLVENLNYSAEGLLKTWPKRFTPVEAKQ
YARQPERIANRVYANRMGNGSPDTGDGYRYRGRGLIMITGHDNYAEAARALALPLVAQPELLEQRTWAAIASAWWWKSRGLNE
LADQGRFERITLKINSGYNGADDRAARLEWARAALKGE

1357536..1357775	PHAGE_Pseudo_PS_1_NC_029066: hypothetical protein; PP_01294; phage	2.20e-
26	-	NONE	NONE	NONE
MLGFTTKDEARQLGVSHHGSYYGIPMWLGDVDSDCPLAFAKWAPLEMVVSLLSVIEGIVNSMLNQEPTFMFKVGRRIDQ

1357772..1358242	PHAGE_Pseudo_F10_NC_007805: Putative lysis protein Rz; PP_01295; phage	8.04e-
95	-	NONE	NONE	GO:0019835 - cytolysis [Evidence IEA];
MTWRPWLVVTLVAALVLWRLDHVTAQRDDLQAAVEQSAETITAMAQQAQRDIQAQVQTDALARTYQAALQASHEENQLRRDAI
GTGARVVYVKARCPADGVHQAPGASGSADAGRAVLAAADGQVVSDLRAGVERRELMIEALRKHIAGLPRYCRR

1358239..1358982	PHAGE_Rhodob_RcRhea_NC_028954: minor tail protein; PP_01296; phage	6.92e-
09	-	NONE	NONE	NONE
MISIKPEGFQQQLADLTELEQRQIPYATATALTRTAQGLMDRLRDEMRVVFDRPTPYTLNSLRMVPARKDRLEARVWFKDEADG

AQPASVWIAPEVYGGPRRNKPAELQLRAKGILPEGKYVVPGAGADLDRYGNIRRGQVTRALSGIRGFSQAGYNANATDSRRSRA
KGNARRYFVMTRKGQPIGIAERTGRGRDAVSVIMAFVSRPSYRRRLSFFEIAQQYADENLPREFEVAMRGVAARFAARR

1359102..1359647 PHAGE_Pseudo_F10_NC_007805: Putative small subunit (Nu1 homolog) of DNA packaging dimer; PP_01297; phage 5.67e-129 - NONE NONE NONE
MSTEDLQKKRGWLNKSEMAASLGISPQAFDKWGVEPAAKIGREVFYTAQAVLQNRLDHVTQKQQPEGLDAEGLDPLAEKKLLQ
ERLRLTTAQADAQEQKNQVQAKTLVPTPFATFALARIASKIGSKLETVCKTVRSQIPDTPPLVLEAFEREIALARNLAVEFAEDLPEI
LDEYSATLDE

1359619..1361598 PHAGE_Pseudo_F10_NC_007805: Putative large subunit (GpA homolog) of DNA packaging dimer; PP_01298; phage 0.0 - NONE NONE NONE
MSTLPPWMNDLRKAVDLGLQGLYKSPPMTAVEWAEDPDDGFYMSAESSYNEGKWKTAPFQVAILNAMGNDLIRVVNFVKSARI
GYTKMLMANIGYKIQHKRRNVLMWSPTDPDAEGISKSHVNGLIRDVPVLLALAPWYGRKHSDNTLDTKVFANRRTLWTLGGKAA
RNYRERSADEVIYDELSKFDADIEGEGSPTFLGDQRLRGAVYPKSIRGSTPGTEGQCQITKAADESPRRLRYYIPCPHCGHEQTL
KWGGKDCAFGVKYIANDLGEASSVWYACENERCSGTFEHHEMVVASERGRWKCEVSGVWTRDAMEWFGPDDQPIRTPRSVA
FYCWAVYSTWTSWLDLIDEWLKVKGDREKLKTFTNTILGEVWVEDEGERVEWQTLYARRENYPKVPPQALVLMGGIDTQDDRY
EGRVWAFGLGEEAWLVHRFILTGDPASEELRRKVGLEIHRQFTRADGVPMRVERWCWDAGGHYSDEVEAESIKHGVHWVVPT
FGASTYGKPIANFPKRRKRKVYKTELGTDNAKELIYSRLRIDVPIPWQPTPGCVHFPIDSDICDEDELKQITAEKKKPVMAKGVRVL
RWDSGGRRNEALDCFVYALAALRISQQRFGLDLDQLERVRVDPVPEPVAQQQPSNDNHASTSQGWLNTGSGPWL

1361589..1361804 PHAGE_Pseudo_MD8_NC_031091: primosomal replication protein; PP_01299; phage 4.73e-45 - NONE NONE NONE
MALTAQQMLDKYLEAEAAVLEGRTVIFNGRTHTMEDIEKIRAGRQEWERRAAAERDRAAGRRPGPALAEFC

1361804..1363450 PHAGE_Pseudo_F10_NC_007805: Putative portal protein; PP_01300; phage 0.0 - GO:0044423 - virion component [Evidence IEA]; GO:0005198 - structural molecule activity [Evidence IEA]; GO:0019068 - virion assembly [Evidence IEA];
MNLIDRLLEPLAPELVARRLAAREAIQAYEAARPGRTHKAKRQPLGADTSLQKSAVSMREQCRKLDEDHDLVTGLLDRLEERVVG
GSGIGVEPLPLRLDGSVHAELAMEIRSAWAEWSLSPETSGELTRPQVERLMCRTWLRDGEGLAQKLMGRVPNYTFATSVPFAL
ELLEPDYLPFSYNNLSKGIVQGIERDTWRRKRAYHLLKDHPGNLQTLGGSLAVKRVEAERIIHIAYRKRIGQNRGVPMLHAVLIRLA
DLKDYEESERVAARISAALAMYIKKGNPDSYTVEPGKDRKNRTIPIAPGMVFDDLEPGEDVGMIESNRPNPFLEGFRNGQLRMIG
AGTRSTYSSVSRAYDGTYSAQRQELVEGWLGYDLLQHEFIDYWCRPVYRSWLQMYLLARKERLPADVDHRTLYAAVYQGPVM
PWINPMHEANAWELLVKAGFADEAEVARARGRDPRELKKSRETEIKANRAAGLVFSSDAYHQLVKSGMDPVEAVQKVYLGVGK
MLTADEARELVNRYGAGLPVPGPDFPNESNNGGADGQPSNPDP

1363422..1365503 PHAGE_Entero_c_1_NC_019706: head maturation protease; PP_01301; phage 0.0 - NONE GO:0004176 - ATP-dependent peptidase activity [Evidence IEA]; GO:0004252 - serine-type endopeptidase activity [Evidence IEA]; GO:0006508 - proteolysis [Evidence IEA];
MGSHQTLIHKSLMLPMAAALTEANAPHESWYSIKAAGRGVAEVLLYDEIGVWGITALQFARDLKAMGDLNKINLHIHSPGGDVFE
GTAIYNLLRNHPASVDVYIDGLAASMASVIAMAGDTIYMPENAMMMVHKPWGIQGGDADDMRRYAELLDKVEDTLVMAYANKTG
KSADDIKALLKEETWMNGREAVAAGFADQLTEPLQAAAHLSSKRMQEFAHMPEALKTLLAPRAQTPAAPTNTPAPTPAPAVPAA
PVAAAPTEADIRARILAEESGRRSAITAAFGAFASGHAELLATCLNDMTITVDQAREKLLAAIGADTKPAATPGAGAHIHAGNGNLV
GDSVRASVLARIGRGERQADNAYNGMTLRELARASLVDRGIGVASLNAPQMVGLAFTHTSSDFGLILLDVANKSVLAGWEEAEE
TFPLWTKPGILTDFKPARRVGLGEFSSLRQVREGAEYKYVTLGERGEQIILATYGELFSITRQAIINDDLQMLSDIPFKLGQAAKATI
GDLVYAVLTGNPAMSDGKALFHADHSNLLTGAASALSIDSLSKAKTQMATQKAQVEKGKGRTLNIRPGFVLTPVALEDKANQIINS
ESVPGADVNSGIVNPIRAFAQVIGEPRLDDASATAWYMAAKKGSDTIEVAYLDGVDTPYLEQQEGFTVDGVASKVRIDAGVAPLD
FRGLQKSNGA

1365570..1365887 PHAGE_Shewan_1/44_NC_025463: minor capsid protein; PP_01302; phage 1.67e-10 - NONE NONE NONE
MAKNYVEDGNVLTLIAPAGGVQSGVPAVIGDLVVVPLVDAAAGEPFAGKTGGVWSLPAAAGLTQGAKCSVLDGELVAAATADSV
AFGKITEPTVDGFASAMLIQQ

1365884..1366213 PHAGE_Pseudo_MD8_NC_031091: hypothetical protein; PP_01303; phage 2.27e-73 - NONE NONE NONE
MSAPGRFGRLIQRLHERGQERLSDAVGEFRGIGRPPIKGIPLQVDRNLSYDGPDGVFITDKVGISWLAKDVPTASRGDLFVIGSSR
YLVEKLIANDGWLLTAATIEEEA

1366210..1366680 PHAGE_Pseudo_F10_NC_007805: hypothetical protein; PP_01304; phage 3.08e-109 - NONE NONE NONE
MKPNVLTIGRLALLARLQTITPNQGYRTDAGTRVLSGWFNELVKERHEGFPLIVVQPGKEQPPEHLDAAVRFHRGFDVVGAVQG
GYDHYEEALEDLQLDLLACLMPAPKGQFLRWLPRERGITGVTLGAPEPYPPGDGVAAAVIRIPVYLKTIIEA

1366684..1366878 PHAGE_Pseudo_F10_NC_007805: hypothetical protein; PP_01305; phage 4.77e-38 - NONE NONE NONE
MKSDPQVPAAVDAAPPAALNKAVEVTLAKVHWHQGKEKAAGEKINVSPDQVEFLRREGVIKKEA

1366880..1367632 PHAGE_Pseudo_F10_NC_007805: hypothetical protein; PP_01306; phage 0.0 - NONE NONE NONE
MAIEKETYVIGGPFKIRESGATTPFQFAGLVSTIQQTIETNEITLPDTTTPQGGEYDAVSRITSVGLSINFRELKTSILAALVWGDATN
VPSATHTDEAHTAVPGGTIALDFMPLEITSVKSDDGTTTYEEFDDWNMTGAGIEIVEGGAISAATPIKVTYKSATVDVIEALTNSGK
TFECLFEGENAAGTQRRIQARYFRCRLNPSSQQDWLNTEDFLAAEATAKVLMDPTKVGAGKSKYFNIKKELATV

1367714..1368235 PHAGE_Pseudo_MD8_NC_031091: prepilin-type N-terminal cleavage methylation domain-containing protein; PP_01307; phage 3.26e-126 - GO:0015627 - type II protein secretion system complex [Evidence IEA]; NONE GO:0015628 - protein secretion by the type II secretion system [Evidence IEA];
MYSRSRGFSLIELMVVVVLLAVLAFMAVPSFKAMQEGNNHLAGKEVFLQHLEFARSYALSKKTTVEVCAESGGWTDGYIVRTDS
GKTVLLKENKYKNIHPVGAWKGSMESGCVRFVSNGSAPAVPAPAGEYYDSGFFGGEELDKAAWRVTFKPSGWNCTEKDPKDP
KCAKKPT

1368307..1368834 PHAGE_Pseudo_MD8_NC_031091: hypothetical protein; PP_01308; phage 1.02e-124 - NONE NONE NONE
MTRIFPVLALILAVSSASGATVFKCVGPDGKVTFTQQNCPDNQSLNDVVSATNQRPSGSGASAVMAKPKQPSGRTYRGSHQVG
SGVIVVGGSSPSPTCSTGLSERDLRKAKVQGKVVPGMSREDVESIYGKVNRNGSTAGAGAVTYWNDKYVDQTTVSFDRNGCV
QGSYQSGHKN

1368899..1369201 PHAGE_Pseudo_MD8_NC_031091: hypothetical protein; PP_01309; phage 5.12e-66 - NONE NONE NONE
MSSFTASRVVDIDGVELTVRELSVADVRKLMQEVSDQDLVNNVLFEDIRLSDLCLMTSVTKSQINDLRPSQLAKLRDACKEVNPH
FFGMLGRLSKLHDKP

1369304..1371787 PHAGE_Pseudo_MD8_NC_031091: tail length tape measure protein; PP_01310; phage 0.0 - NONE NONE NONE
MADVKIRLTADLDDALREVSGFRKEYAELVRQVAQPLKRLNDFTALESTLEDTQRQARSAREQIRTLGNELASTIRPSRELQQAY
RDSISDLRSLERAETVQVAKLGAMRRELKQAGLDTRSLTSERQRLQRELDRNLQAGRNDAATTSLRQQAAAIKQSAIEQRRFNLE
QARSTLGVARVRELQAAIGQLNQQYRLLRSSGTLSTRELAVAQRALKKQIAETKSELNSLGAGSRLSSIGSLRGSGPALAVAGLA
AAVGAATAKLANGADTVGRLDSRLRLATRSQEEFNTAQIELDRIADDVQGDVGDLIGLYSRLQRPLRDAGMDQRAALETVEAVSL
GLKIGGASAEESASVITQFSQAIASGVLRGEEFNTVLESSDRIAGALADSFGVTVGRLREMAAAGELTSEQIVIALRKELPKLREEM
ASFAPEIGAGLNRIFSETQKYWGRRAKETGIVDWVANQLNDVAKGINTANTLVKKGEGSLTATLAAEKARQEQIVKRQNDALKRA
RDQNVADLQSEVVRTKALLEQSTKNLNDALSRQADVRKEFADLVKGIQATPTSGTQTFGDATAAQASARNALTAGNNQKAIEEA
RRALQILQQLKDAGANSYGFEGVAKEVERIANKAAEVEAGNAKAADDVNRLNLADLEERIKAVQNVEVSFGMDFESAETLKQQV
ADIAAGLAEQLVIPITLVPPPEMGLPGVPSITPKIPGFATGTQSAPPGMAWVGERGPELMMMRGGERIFNAVQSLQMSQRYQRTL
PEIPEIPTAALQQANPPAAMQNLGSLTLNLGGDDAGFTVFGTHDTLRDIRKAASKFGRTRPK

1371828..1372349 PHAGE_Pseudo_F10_NC_007805: hypothetical protein; PP_01311; phage 9.42e-123 - NONE NONE NONE
MIIPNVMLGGVPIVIHGGAPQCQYQAVDGGVERLRLSGGAAVQMTHWRKTAITISGSGWIGTGMLGLDFDNPLELRCNASLGISG
RTAADRVFTIPGEVRPDASPWGLALVGREWVRTDVSSAGQVVTVSEIPGAQLYRVEWWPLFHVFASVPPEALDSSNNSRTWQI
VAEEI

1372349..1374046 PHAGE_Pseudo_F10_NC_007805: hypothetical protein; PP_01312; phage 0.0 - NONE NONE NONE
MLNGGPLNSAELNSAAQSVVPGPEPIIPGYAFTWRAIVRVGDDDVTPLLTGEIEVDREEGAAGVASFSIYLGDGPVVPTDWIGRT
VTIDYATETAGELSQGRRFTGRVTQPAWNPVRRVLDVSCTDQLQQRVEAMEIAAVDALVGGAWSADVFEPVDGRSRWDYAQE
RLTSVTGSLDCSPYGALRVTSWLSVAPAFEFGQGSTVYGSLAVELADLSSQTNRIEIECDYRFSRLWQLNASYGWQHPGTGNAV
GEAGFCNWRGDDTELPDVEMITSATESSGQTLFYATWYPLPPTGVYCNPPAAWRNDFTELLLGGNWIAGRRWVQSVTERYRL
VMEVQPSVAATGPIVGRQRASFEIESDKAERWESDPITGGSTGHDDEKDGSRRLSALNCLLAQGATTLIAAHRGTTVTWDVPTS
MVLPIDLVHTLRLDDQGARAVGKCRRIVDRLDLASGSALTTISIAVMRGGGGAADPLVPPAGSSGPVSPPSGGGQLSTQLGGRN
GSPPYDDDADGFSGNWSNRDPGAELFPRRFSLTANDIPETYRDEHAPEIAATYRVSVPDDVLEM

1374049..1374459 PHAGE_Pseudo_phi2_NC_030931: tail fibers protein; PP_01313; phage 5.08e-70 - NONE NONE NONE
MARAWINNWKTTLSAGLAPGATSLTVPDAAAALLPLSGGSWVLLTLADAAGAQHEIVKATVRAGGVLTIERRQEGTTDGTWPAG
TAIYAAVTAGDLMALQARIAALEGGTPDGALVDASGSALVDGAGNNLIMENI

1375016..1375438 PHAGE_Pseudo_MD8_NC_031091: hypothetical protein; PP_01314; phage 1.81e-92 - NONE NONE NONE
MALSDERRGIGARNEAIRRAGGQRVEAERRGDQGLTAALNRLIEPERQARALRKIDPRGALDAKRGRADYNPAGKQLGGGGGIA
SPLIEEDAAQREYYELQTIPTSDGLAWLRYRSVKKIVMTDASGAEVVMEYANDVSQ

1375599..1376924 PHAGE_Pseudo_MD8_NC_031091: hypothetical protein; PP_01315; phage 4.75e-153 - NONE NONE NONE
MPVPNVETDDPDERWLNRAIMRGTGLAEAYGGVSLQPAFIRGYTVRCGVEVTFNSFLGTASAICSIRDGVTGFVGQITSNTIAPS
ALGIPVQPAGMSFQVLDVNHDGTRRVFRVDYQETVDGTVIAGGMVEVRISPSGASGFQAELVVVATWSQVQFTTLSSSKPDVDP
DTHTRFWWDSASGSYVSGTSDPPGIPVDTRVLQGAWTATLRAESIAAAWYGVSGDLEFVHIEVSVSHSLQRAASAAGDHVAFN
QTDDWVVDYRLRSASGEVLETLRNKVEISGPIYNDGGPGTANITDSISGQTVATGTRPITLQDQNIYTPDVGDTYARGLNWGSRY
HVFPIAADGYLSSFAAQYAWPVQRYSNKMLGIIAVRNSAIGTPDERYRLAGVAFTPHGVHGTRQVDVDVGAYSGIQFEAWSKGA
YNPITGDAIRNDPNAFYSYV

1376947..1377534 PHAGE_Pseudo_phi2_NC_030931: hypothetical protein; PP_01316; phage 1.38e-133 - NONE NONE NONE
MTPACVPLRIEKGATFRDTMRIMQPSLVYRPITQIAPAAPVRLTIPGHGLPGTWLAWIDGVQGMPELNRARLRQLPHRVASIDDNT
VEINLLSAVGLAPVGGQLIYQPPVDLAGAEVRMQIRDAPGGTVLMTLALGSGLEIAGAGTISREISASATAALEWSAAVYDVDVTY
PDGTVHRYYSGPITVSRGGGCDG

1377527..1377718 PHAGE_Pseudo_MD8_NC_031091: hypothetical protein; PP_01317; phage 1.27e-37 - NONE NONE NONE
MDDTAEPWALAIEVDCEPLVLSEMQEYAVTVTPPADVLVVVAGDQGPPGRDGVDGAQWGATDW

1377723..1378283 PHAGE_Pseudo_phi2_NC_030931: hypothetical protein; PP_01318; phage 6.16e-131 - NONE NONE NONE
MAQIRFFKVATLPGTLEPDSFYFVENGSYSESYLTNSAGVARSIGNSAMINALINEALSSLPGTGAPILFVADIAARDALEPESAIFV
LVQDASADSTVESGAALYAWNPATSAWLKVAEYESMDVELNWDAINGRPTSTPAQIDTAVSQAHTHANKSTLDKFSEDGGLVRF
NGQPIPAEWNGTAW

1378283..1378564 PHAGE_Pseudo_F10_NC_007805: hypothetical protein; PP_01319; phage 3.33e-60 - NONE NONE NONE
MAVLQTHKVVAQLPASLEPNAIYFVRRSTGYDQFVTNGAGVVVAYPMNVRIPAAVPGYLADGSMLRLTMNPDGQLPAYTAGGAT
LNLQVLFNG

1378557..1379120 PHAGE_Pseudo_F10_NC_007805: hypothetical protein; PP_01320; phage 1.71e-60 - NONE NONE NONE
MADVRPTKLQADGNGYGSLREFSDGDTVPIALGGTGAATAAGARTSLGLGSAAVRAALGSTGALYSRDSILGSVSQSSGVPTGA
VIQRGSNANGEFVRFADGTQICIVTLLGDGSQQPGTSISLPLPAAFLGNWTTGVSVSWASHVSNPSVANGLKVAYANGSTLFFIL
QDALATNRLIFTLVGRWF

1379120..1379422 PHAGE_Pseudo_YMC11/02/R656_NC_028657: hypothetical protein; PP_01321; phage 9.79e-54 - NONE NONE NONE
MIIKLSPYAPLPGSDEHLSLSRAGDVLTVNGQSFDFTPLPDGGELPAEAIGSEWFAGSAVRRGDRLELILRFPLAADASGAARFPE
PLLIEADGPVELPR

1379419..1379658 PHAGE_Pseudo_KPP25_NC_024123: hypothetical protein; PP_01322; phage 1.07e-42 - NONE NONE NONE
MIDWSKVKTTEQQAQDRRQAEYDAAAAARANAYRLESDPLKTEAEFDAIKAGTEPDYSAWIAKVEEIKARYKLPDAGGV

1379687..1379959 PHAGE_Pseudo_phi2_NC_030931: hypothetical protein; PP_01323; phage 1.02e-59 - NONE NONE NONE
MLVVRLKKGWTLKLDRKVNDANRAGVWSFHCSESTFVPGMDNLLRHAAIRPAEPAEGKSTEVEVAICRPGDPEEKWIPVGKGV
AVYEAER

1380105..1380130 attR N/A
CAGGCTTTGATGCCGTAGAGAACGTA

#### region 4 ####

2508737..2508751 attL N/A
CTTGAAAACCGTCGA

complement(2508911..2509978) PHAGE_Pseudo_F10_NC_007805: Integrase; PP_02388; phage 1.29e-132 - NONE GO:0003677 - DNA binding [Evidence IEA]; GO:0015074 - DNA integration [Evidence IEA];
MRPKQPKNRDLPPRMIRRTRKLKGGKLWVGYYYDGRGEDGKRKEIPLGTDLDLAKLEWARLDASPAPKTLRKWGDVFDRYEKE
IIPGKAPRTQKDNLLSLTQLRKAFSEAPVEALTPQVLAQYRDKRSAKVRANRELSLFSHIFNIAREWGIVTTENPVKGVRKNRETP
RDFYARAEVWNAVYGAAPPELRDAMDLAYLTAQRPSDVLIIREADIQDGHLQIAQGKTSKKLRIMLDVDGSPTALGELVARLCEQ
RRQRGVAGPYLITTPDGRRMTSSMLRIRFDEARSAAAGAALEDLDETLATAIRQFQFRDIRPKAASEIADLGRASRLLGHTDKRIT
ETVYRRVGEIVEPTK

complement(2509980..2510216) PHAGE_Pseudo_F10_NC_007805: hypothetical protein; PP_02389; phage 1.74e-23 - NONE NONE NONE
METPSEFLSKEELEAMIGAKSSKKQVEWLASHGWKYELNAAQRPVVGRIYARLRLAGVKPNGTVAVQEPWTLDLSKVS

complement(2510295..2512658) PHAGE_Pseudo_YMC11/07/P54_PAE_BP_NC_030909: hypothetical protein; PP_02390; phage 9.57e-145 - NONE NONE NONE
MSSTQHQLIEQCATRLRGIVEALDNIHDSTQHRSPHRWSTDLDEVHSSAESLLAMIKDQAPPSEDQLIAAGLSYPLAKEDAVQLW
YAGFRSEVVTVLEAWEAIGHDIGMNPSKGELLDSLRNMAAICNAHGNDMPAQSAIDQRQVIADAITGALAFGAQASQPPAEDHW
LRPFYDIGRAEGQRTQELAMLVRMLAASLKRHAPESNLVARATNYLAAKGLAGTPLRDPPAPVEQAGGDERAAFEAWATHLPM
DRQPLRPDLYMPQTQWAWEAWQFRAALAQPSQSQYEASFEEWLANELEGEDGQPVPAAVCDITLARRAFNHWPKLEQPAKV
GGVRFSAGVSSRLVVEAAQRLNEFESTPEKEAERIERLQAFREQLDPLNLAPHAEAFNEAPDEALRPEQAEAERPEGPTEDELE
AAGLGYPLHKEEAVKLWYSGFRSEVITVLEAWEAIGHDIGMNPDKGELLDSLRYMLEKCEAHDAALARVAELERQQPVAWMHD
QPNRVDVIHRDVKDLLQRVPGSSRGIYRPLDVSEHYTIPLYAAPVAQAQHSVPEISGIGRDAEHPRAVVLYLRNEPSEEDMRAIQN
FLRAISADVLTQAQHSAGYAEARMCANCRHIGINDAADYAACHDCNWTGPEPDEDKCPGCAGENCMAAACPECGCRYDLVAE
ANITAPAGQVPQAWLDVQAERRRQITAEGWTPEHDDEHADGQMARAAACYALAGSSAPNDGTAALLVSLAWPWDEQWWKPS
TARRDLVKACALALAEIERLDRACISQSPQPGATTASS

complement(2512756..2513262) PHAGE_Pseudo_H66_NC_042342: hypothetical protein; PP_02391; phage 2.63e-116 - NONE NONE NONE

MTEPATDYSITAADAKELAGAVLLPADLRRQVLEKMAAQRDPATMLDLFAQVLGMANAVAENCRAMVELILIERGEHPHTAEQAN
LPTMFGALQGVVLAATVNPRGTCAGCAYRLGTPANTSPVTTSDAIYCRQELSRFYCHADLDDQGNPVRTCVGHAKAMKQDATK

complement(2513259..2513660) hypothetical protein; PP_02392 N/A -
NONE NONE NONE
MPNYYPKGGRCRACASRHDDCSSLPFETMPVRRRDQEQTMSEPFQKTFDQTGTFEALYACQQWLSANGYSYSSTCRDGPVG
VMKGDYGIAKWRNLTREERAELHGTVDGDFREGPLVLRLKAGFGPQASGVAA

complement(2513668..2514429) PHAGE_Pseudo_F116_NC_006552: hypothetical protein; PP_02393; phage
0.0 - NONE NONE NONE
MTRSNAPLVQSEAELCAAFIDEFNRVPGWTCYPETAGFDILVVHEDGRQIGVEAKLQLNAKVADQILPQYWQDRYGAPGPDHRL
VIVGRITEASAGIKRLLEMCGIAVLAPSRGHRRRDGKFVDFPEFHLRYWLQHSSGPQLFDWNPAERCHVPIVVPDVPAGVPAPLR
LTEWKEGALKVIATLRRQGFITTKQIAECGVSATNWTRSWLDKGAERGTWVESARMPAFDQQHPEAFTKIQQALDKSAQPTLFT

complement(2514426..2514914) PHAGE_Stenot_vB_SmaS_DLP_5_NC_042082: DUF1643 containing protein; PP_02394;
phage 4.19e-43 - NONE NONE NONE
MSAIISECGQYRYLLTRPGDCLADKGTAVFLMLNPSTADAALDDPTIRRCRNFASAWGCNGIAVVNLYALRATNPSDLWQHSDPV
GPDNDWRLRAIAREYTDIVCAWGANAKPERVEAVTSILTAAGGRLWCLGTTKDGHPRHPLYVPGNQALQPWAPRVTP

complement(2514911..2515588) PHAGE_Pseudo_phiPSA1_NC_024365: hypothetical protein; PP_02395; phage
1.18e-74 - NONE NONE NONE
MKERPILFTGPMVRAILEGRKTVTRRVMKPQPDFLGSMVDPNTPFKALDAGLHARIICPYGEPGDRLWVRETWHVGKPHDKTAP
ADILAPLLAEGRGITVLYTAGGWQSVGPAGREEPIYPDDQPLPDWAGKGRPSIHMPRWACRILLEITAVRVERLQDISEVQARAE
GITDGGCSNCGNHEPCGCECPAPSAVDSFVHLWRSTGGNWESNPWVWVVEFKRVTP

complement(2515585..2516028) PHAGE_Pseudo_YMC11/07/P54_PAE_BP_NC_030909: hypothetical protein; PP_02396;
phage 2.95e-76 - NONE NONE NONE
MNTEQFIRNAAARGLSRRATMQALGLGRWKFDLIIGAMGPIEWAKNGTTLGNRLAYEASRGRFTPAQAAALERAHERWSESRR
FTVDGVTGTIAELVEHFQSPVHATTVRRRVAAGMSLRDALLSPRQQPKPGRRHPWNRSQKQVQP

complement(2516025..2517803) PHAGE_Pseudo_phiCTX_NC_003278: hypothetical protein; PP_02397; phage
0.0 - NONE GO:0008168 - methyltransferase activity [Evidence IEA]; NONE
MIKRTLYHFHFCCGLGGGAAGFNRARPRVGNVEAEWVCLGGIDVDPAGLRDFERLAGVPGTLLDLFTRDQYIRFHGTEPPAGW
REATPEDIRRAAGGRRPDAVFISSPCKGASGLLSEKMSLTPKYQALNELTLRCIWLMGEAWADDPVPLIVFENVPRLASRGRHLL
DQINSLLGGFGYAVAETTHDCGELGGLAQSRKRFLLVARHVEKVPPFLYEPEKKSLRAVGDILGRMPLPGDIDAAGPMHRVPSLQ
WKTWVRLALVRAGSDWRSLNDLAVEDGYLRDLIIVPKYRAGYMGVHGWNDSTGIIAGRSSPTNGAFSVADPRAPANALQYQQY
GVRRWTDTSGAIIGVKSPGQGTYSVADPRGQSFGKYPVTDWDGPAGTVIAASTTGQGAFAVADPRHRGPAKHSNEFRIVPWDR
HAQAVTSAHGTGQCVEDPRVLSRTKGDPYLTGGHYGVVGFDQSAGAVSASARHDNGRWSVADPRMPKANDRLTCIIQSLDGT
WHRPFTTLELAALQSLVDPEEQLILDGLSDSDWRERIGNAVPPAAAEAIAGVMGTTLLLAEAGETFMLSNTPIWVRPVAVALSVAQ
QEVNP

complement(2517800..2520430) PHAGE_Pseudo_YMC11/07/P54_PAE_BP_NC_030909: hypothetical protein; PP_02398;
phage 0.0 - NONE NONE NONE
MTAYEDFLRAKVRLAEPKGFEVKPSAFHPLLKPHQRAIATWLVRQGRAACFAAFGLGKSVMQLEVARVTRELAGGYALITIPLGV
RQEFYRDAAMLGITVRFIRSFDEVDDPNTIYLTNYETVRDGKLDPRRFSVASLDEASCLRGFGGSKTFREFMALFAGDDRAAGIR
GDGVRYRYVATATPSPNEYIELLAYSAFLGVMDVGQAKTRFFKRNSEKADQLTIHAHKEGEFWMWVASWAIFVQRPSDLGFSDE
GYALPELDIRWHEVPSDHSHAGHERNGQGRLLRNTAIGVQDAAAEKRESLPARIAKLMEIRAEAPDAHRIIWHDLEAERHAIEAAV
PTAVSVYGSQDLEERERAIVQFSDGEFQELAAKPVIAGSGCNFQRHCSWAIYLGIGFKFNDFIQSIHRLHRFLQTGRVRIDLIYTEA
ERDIRRQLERKWQQHNTMVQRMTEIIKQYGLSIAAMAQTLTRSMGVERIEIKGKDYTIVNNDTVLETRRMENNSVGLTITSIPFSTQ
YEYSPNYADFGHTDDNAHFFQQMDYLIPEMLRVTIPGRLACIHVKDRIVPGGMTGLGFQTVYPFHMEVTRAFVKHGWAYMGMK
TIVTDVVRENAQTYRLSWTEQCKDGTKMGVGMPEYLLIFRKPPTDNSNAYGDIPVVKAKPLCIDEDGQIVPFAMDKKLTVTRGNG
YSRARWQLDAHGFTRSSGNRPLTEEDFEGIPHDVMFKLYRDYSLSTVYDFEHHVRIGESLEVTGKLPTGFMLLPPQSWHPDVW
TDVARMRTLNAQQYSKGQEMHLCPLQFDIVDRAIVQYSMEGDLVFDPFGGIMTVPYCALKLKRRARAHELNSRYFLDGAGYCKS
AEEEMAMPDLFALLEADADIIHKEPAA

complement(2520577..2522319) PHAGE_Pseudo_YMC11/07/P54_PAE_BP_NC_030909: hypothetical protein; PP_02399;
phage 0.0 - NONE NONE NONE
MRITKLEITNFQGLRHAALDVSAPVLLVAGHNGAGKSSLLDAIAMAFNGQPRRVSLKKEMDKLVTEGAKKGEARVEWLDEAGEV
QACGVALPSGKGSPLADSPFLPYVLDASLFAALDAKDRRRVLFDLTGASASPAEVGKRLKAKGIDLALFEKVKPLLRSGFSAMVG
QAKDYASEARGAWKAVTGENYGSEKAIDWAPELVATAVTNDQVEEARNALQALEDDLAEAQQTLGASKQARQAADGRAQRIAN
LRELVDLEPRRRNKLSTDEQNQDEWSEKVMAAELASSGSVPHQPLTCPHCQGAVDLQAGALVVHQPPEKIADAEAAKRLPEYR
EYLASAQRAVANSQRDLDECLAAAEQIKALETESADAPSAEAIANGEQAINELRQARDASRAKLVALQEAMEAAAQREASIAKAQ
AAHRDVVAWTGMADALSPTGIPAEILADAIGPVNELLQRLSGTAGWSPVQISADIDVTFGGRLYGLLSESERWRCDATLALTIATIS
GLRLALLDRLDVLDLPSRSQALTLLRAVTMDKEIDSVIVAGTLKEAMAKTPTWLQAVWIDAGQLADQQQQAAA

complement(2522323..2523090) PHAGE_Pseudo_YMC11/07/P54_PAE_BP_NC_030909: hypothetical protein; PP_02400;
phage 3.02e-161 - NONE NONE NONE
MSQNNAAFLHMTADTLGKSLLQGLIQEIRILPDVWQKLSEAKQTDVIERLEQQVRNAATIAVHTIAGAERETVYGKLESIAAKDKMK
AVIVVNHSSPNKHDLLDAVNEDCLLIIGGAAEFLDGMKDVKADPDQNPLDLNGGDHDMEDPGAWGGMQPADDSDVVDAEFQEL
PQLTVERFAGHTLGEIAIGVATKKDVFDAAWLQSRFALTTEEAERVILQLLDQGVIVLEQENEESRELNTYRVVKKPGDIALDLE

complement(2523101..2523301) PHAGE_Pseudo_YMC11/07/P54_PAE_BP_NC_030909: hypothetical protein; PP_02401; phage 5.34e-42 - NONE NONE NONE
MPSRTIEEQFDRVEEFNSLLGAAELNAATTWEEEFTADLRANFQRYGPRMFLSESQHTTLERIANQ

complement(2523308..2524207) PHAGE_Salmon_epsilon15_NC_004775: RecT; PP_02402; phage
2.13e-141 recT NONE GO:0003677 - DNA binding [Evidence IEA]; GO:0006259 - DNA metabolic process [Evidence IEA];
MSATALKAAATGNVANNGQPKTLAHLMTDPKIKAQMALALPKHMTADRLARIALTEIRKVPALAKCNQESFLGAVMQCAQLGLEP
GNALGHAYLLPFGNGKAKDGLSNVQLIIGYRGMIDLARRSGQIVSLTARTVHQNDQFSYRYGLDEDVQHVPGEGERGVMTHVYA
VAKLKDGGVQFEVMSKADVDKVRATSKASGNGPWVTHYEEMAKKTVIRRLFKYLPVSIELQTAVTLDERADAGLDQDNASILTGE
YSVVDDQSQDQVPDGVNTETGEITEPAPGQQSDTGDTGDDGLNLE

complement(2524220..2525128) PHAGE_Salmon_epsilon15_NC_004775: hypothetical protein; PP_02403; phage
2.24e-151 - NONE NONE NONE
MSLLKIAPEHHDRSKLLGGSDVAGILGISPWRTPLDVYLDKVQPRTGAVDPAKQKIFTRGQRMEPYVIDLLAEETGLKIVGRGNRY
RDQQHDFMAAEIDAEAASGENIEIKTVSPFKAKDWGEVQTDAIPVHYTAQAMHGLMVTGRQVCIFGVLIGGDDFRVYRVERDDE
TIAAIREKEVEFWGRIQRLDPPEATAVSDILRLFERDAGTSIEADGKVVEVFNRLRELKAKAKGLEYEIESAEERIKLFMQDHAQLTV
NGKSVLTWKSQTTNRFDQSAFKEAHPALFEQFKKTSESRVFRLK

complement(2525103..2525600) hypothetical protein; PP_02404 N/A -
NONE NONE NONE
MTATLASVGALDRTKYLGGQQSKRVPLLNEGLVRHLYESGMTLEEVSAEIGCTCRALRLFMIRCGIERRIAAKRDQRGAKNSSW
RGEAVKYKPAHNRVYAARGRPMKCEHCGTTDPKARFEWANVSGRHHDPNDYIRLCRSCHCKYDGLLKNLGDYACVPPQNRT

complement(2525611..2525820) PHAGE_Pseudo_D3_NC_002484: hypothetical protein; PP_02405; phage
4.60e-05 - NONE NONE NONE
MTTRPVRSIIDDQLDDIEEFAGKSIRQAVELANRHGYNNPFFADICGDLCVLRFRRSSRLHATTTLTLK

complement(2525817..2526038) PHAGE_Pseudo_phi297_NC_016762: hypothetical protein; PP_02406; phage
2.24e-45 - NONE NONE NONE
MAWANERAEGVIEEAIVAMRRSVIPRHDQLVWRGQIEMAYTLDAIGTRQYDDMRRRLDAAADARQQELRSIDL

complement(2526022..2526174) PHAGE_Pseudo_H66_NC_042342: hypothetical protein; PP_02407; phage
1.04e-15 - NONE NONE NONE
MWTYRERRNRAAFSNAQHTWDFARDPLWDQPEPEPEHEDEEQEDDDGLGE

complement(2526583..2526831) PHAGE_Flavob_Fpv10_NC_031932: hypothetical protein; PP_02408; phage
2.07e-05 - NONE NONE NONE
MRRVMTMKVTCDRNGRRTGLEDSGEALFHQWGVDFEEFETGAGNYTVAVIERPGGTVELLPVHHIRFLDTAPDFPDTEDLTM

complement(2526845..2527213) PHAGE_Pseudo_YMC11/07/P54_PAE_BP_NC_030909: hypothetical protein; PP_02409; phage 7.38e-82 csrA NONE GO:0003723 - RNA binding [Evidence IEA]; GO:0006402 - mRNA catabolic process [Evidence IEA];
MLILTRRVGETLHIGDNITVTVLGSQGDQVRLGITAPDDVAIHRSEIYQQIGNVRPVPPAELVEAWNREHPAPALIEYRPYRGAEPQ
RTRTVGRASVSLGGAAVIWIEGQSAPVALRACTAI

complement(2527248..2527460) PHAGE_Pseudo_H66_NC_042342: hypothetical protein; PP_02410; phage
7.57e-44 - NONE NONE NONE
MKRNANPAATVAAWNSAYPVGTEVDYRFHRAAAPKRTRTTTEAQVLGGHTAVVWLAGVSGCVALSHCEPA

complement(2527523..2527744) hypothetical protein; PP_02411 N/A -
NONE NONE NONE
MRVHEKFSKKGARPLECVQPTVRTIAGAVHPVAVEFFQTSDSCDGRTLTAFMTPQEAMKLALHLLHVVQGAMR

complement(2527949..2528227) hypothetical protein; PP_02412 N/A -
NONE NONE NONE
MNQISIVGYESDCNCEHCGRALKHGVRLSDGRLVGATCLDKKLTKPRQYKGKSFRFGAEHIIKIAKVVQFYSPSNWARFGVSASS
TTFEGIA

complement(2528287..2528454) hypothetical protein; PP_02413 N/A -
NONE NONE NONE
MNAYKAGDKACYLGRARATVLGKTSRGYRIEYWGQGARDGELIRATVPARDLMPI

complement(2528969..2529175) PHAGE_Pseudo_phi2_NC_030931: hypothetical protein; PP_02414; phage
7.46e-44 - NONE NONE NONE
MAQFNVDAHLSNGKRLDWIALPEGNETPDDVLIKVRQAAMKKFGDLIWFNRWDHVVASNGYITVRMHA

complement(2529186..2529488) PHAGE_Pseudo_phi2_NC_030931: hypothetical protein; PP_02415; phage
4.16e-66 - NONE NONE NONE
MTRMQVRRNTDFTGWPSTEGQTGSQHALEEQKMNEKSSRAVRQALRVLRKAEDDREARIEYHETVGMLRGLYYGGEIDSMEL
VALTQLAGNAYINAGKPW

complement(2529853..2530341) hypothetical protein; PP_02416 N/A -
NONE NONE NONE

MRLIAIAAIMIMLSGCAVSQQKPVPRIPFPAAEFAALPTKGTGTLTGQVFMKTVGGDVKFGAGSTVYLVPVTSYSKQWYEVNYIGG
QALEAPDPRSGQGSITTVADGNGNFTFTDIPPGDYFLSSTVTWQAPSKYGLLPQGGVVAKVVSIADGMKLREMLTR

complement(2530389..2531054) PHAGE_Pseudo_JBD44_NC_030929: cI repressor protein; PP_02417; phage
4.90e-56 - NONE NONE NONE
MDFSDRLNQRMDALGISAADISREIKVSKGTLSHWTNGTNKARGKNLIALAKVLRCSASWLETGKGEKELPAHEGAPSEADYALI
PQLTAKGSSGNGYLNDHVEVKGGLAFKRDWLRRMGLKAENLRVAYNQGDSNWPTLSDGEVVLIDVSCKEPANGKMFALHDAD
QEVIFKRLIREISGGWLIRSDNQDKNRYPDQPVTDDGMRGVDIIGRIVWRGGAM

2531344..2531655 PHAGE_Phage_Gifsy_2_NC_010393: bacteriophage transcriptional activator; Lambda gpCII analog; PP_02418; phage 9.24e-20 - NONE GO:0003677 - DNA binding [Evidence IEA]; GO:0006355 - regulation of DNA-templated transcription [Evidence IEA];
MTASQLNPERDARAREFESLVLNRLLSVGQKTVADAIGVSESTVSRWKEGEIERWCKVLALLELQVVPMSAQCHPFEYIQALKTL
AELGLQAEKKRPGPLGWD

2531924..2532106 PHAGE_Pseudo_YMC11/07/P54_PAE_BP_NC_030909: hypothetical protein; PP_02419; phage
1.46e-36 - NONE NONE NONE
MQIPEPLVPLECDVRDSPIPTDMLIELAMTIFGLSMEEAESKVRAAISDNPVNLSEIGHG

2532099..2532899 PHAGE_Pseudo_YMC11/07/P54_PAE_BP_NC_030909: hypothetical protein; PP_02420; phage
0.0 - NONE NONE NONE
MANQWFRMYAEFATDPKVQMLSEVDQRRYIMLLCLRCGNGDVTFHDDEVAFQLRINSEEWAASKGRLLGKGLITEDNIPANWD
KRQFSSDSSTARVAAHRARKKQECNVSRNSNGTKANALDTDTDTERDSPTDVGLVDASPQPGQSNDQDLFEPDQPEHLNGHQ
HGIKPCPAQAIADLYHQVLPELPAVALLNDTRRRHLQARWREHEAHRSLDFWRELFETVKASPFLMGNVPGRNGAKPFRATFD
WIIAPSNFVKIVEGNYHA

2532892..2534307 PHAGE_Pseudo_phi297_NC_016762: replicative DNA helicase; PP_02421; phage 0.0
dnaB NONE GO:0003678 - DNA helicase activity [Evidence IEA]; GO:0006260 - DNA replication [Evidence IEA];
MRDPFSLEAEHGVLGAMLLRNELIDVLSAELTPEDFYWPENGDLYRAILALHSDSQPADIVTVGEFLGDRYQVQTTDGMITGLAYI
GQIIQNTPSVANAGTYSRIVRERAVDRALAAAGDRLHELALSEAAQADKVGAAQAMVMALDSKTSTHEVRHAADVLTDHIEELQR
RSDLGGKLDGLATGIGDLDQKLMGLKPGDMVVIAGRPAMGKTALAINIAEHVACDLGDPALVVSLEMTNGGLMDRILASLGRIPLT
AIKDGSAPSSHGAELGSASLKVKRSKLYMADRPGLNAARLRALARRHKQRHGLSLLVVDYLQLLESSGKSTRTEDVSDMSRQCK
LLAMELGIPVIVLSQLNRSLEQRPNKRPMMSDLRESGAIEQDADVIMFVYRDEVYHPDTQYRGVAELIIAKHRNGEPSTVRCAFLG
KYSRFEQLAPGALDEFDFDEPQQAPKVTSMAERYRGMKGGRANG

2534300..2534743 PHAGE_Pseudo_phi297_NC_016762: holliday junction resolvase; PP_02422; phage
1.42e-68 - NONE NONE NONE
MADLRPVMFTVPGEPQGKGRPRIGRVGAHARMFTPAKTVAYEGLVAMAAQQAMAGRPLITRPCLIEIWMYHQVPASWSKRKRA
QALAGEIAAMRKPDADNCLKAICDACNGVVWRDDVQATRGIFQKLWSETPGVRVKIVPLLEGEQ

2534772..2535641 PHAGE_Pseudo_YMC11/07/P54_PAE_BP_NC_030909: hypothetical protein; PP_02423; phage
0.0 - NONE NONE NONE
MRLISARQAWHDAFYESRSSVLAVAADKAALGKKGRVANETHPDRKDTNGRSAHMLAAGLVQAAIRSLPKPLQHFGHTLYSPLA
TGDDVAIAHGLVWIGAGLGQLTQRQGERAYWMALAAINSHKRAVNGRDTLRPAEVCLFIEERLGCRIDPGNWARDYASTWERLA
RHIDKLDAQALRPVAEVVAKQSGLRKGPGWRWHQVDRDVVALQRAEAYAERREHHQQRLAERLRGMSDQELARWAARMKRY
GEAYREEWGEDILECPSVHQRYHDRVAAYWTQRERLKRVA

2535756..2536145 PHAGE_Pseudo_YMC11/07/P54_PAE_BP_NC_030909: hypothetical protein; PP_02424; phage
6.44e-83 - NONE NONE NONE
MAEPTSSGAVAAAGAVGLTATAIIPGVDVNAVIGGFAGALLFVLWAHDLTIARRVGYLLASWVGGYYAATEAVGRGATQFSGLPA
LVTAALIVTILIGVLDWMIGGRAPAWLQIVLQRIVGMIGGRKDG

2536138..2536422 PHAGE_Pseudo_YMC11/07/P54_PAE_BP_NC_030909: hypothetical protein; PP_02425; phage
4.99e-59 - NONE NONE NONE
MVDLVTLTAAAVCGAISCRIFTYQRHGATYRFGVSLCAYILAAGTGMQALSISLAVLMARHATPISPYLLAVLLVLLVLVYRNKGNIA
PILRLS

2536431..2536973 PHAGE_Pseudo_YMC11/07/P54_PAE_BP_NC_030909: hypothetical protein; PP_02426; phage
1.76e-129 - NONE NONE NONE
MALTAKQRRFVAEYLLDLNATQAAIRAGYSKNRASEIGYQLLQKPDITSAIQAAMKERAERTRSDADYVVRRLEEIDQMDLLDIVN
DDLTLRPLSQWPKAWRQYLSGFDLAEMFEGKGDSRAAVGILKKIKWPDKVKNLELLGRHHGVFTDKFEHSGPGGGPIPTMPTMI
ELVAPGESTD

2536957..2538210 PHAGE_Pseudo_YMC11/07/P54_PAE_BP_NC_030909: hypothetical protein; PP_02427; phage
0.0 - NONE NONE NONE
MKARIELPPKLIPVFSGPARYRGAYGGRGSGKTRSFAKMAAIRAYMFAEAGISGGQILCGREYMNSLEDSSMEEVKQAIRSEPWLN
AYFEIGEKFIRTRNRRVWFSFSGLRHNLDSIKSKARILIAWVDEAENVSEIAWQKLVPTVRECDSEVWITWNPEKDGSPTDTRFRK
NMPAGAKIVELNYTDNPWFPDVLDQERLNDRESLDDQTYAWIWDGAYRENSDAQILSGKYRVAEFTPEPGWDGPYYGLDWGF
SQDPTAGVKLWVHDRRLWVEYEASKVGLENDDIAQFMIDRLPGIELHAVRADSARPETISHVKSKGRDHKRANLPRIEPVAKWQ
GSVEDGIAHLRSYVEIVIHVRCTGFLREARLYSYKVDRLTGDVLAEIIDKNNHFMDASRYALGPLIKRRGAVGMLLPGAR

2538210..2538407 PHAGE_Pseudo_phi297_NC_016762: hypothetical protein; PP_02428; phage 3.63e-40 - NONE NONE NONE
MAIFILKERATSRSMVVRARCTTCARTVAVENAGAEGTMVWRDPNLSSVELVRETDKPGLILKSD

2538410..2539729 PHAGE_Pseudo_YMC11/07/P54_PAE_BP_NC_030909: hypothetical protein; PP_02429; phage 0.0 - NONE NONE NONE
MTDKLDLAVNHAMSSAIARARMSLLNQGIGHDAKRPQAWCEYGFPQEITFNDLYTMYRRGGIAHGAVEKIVTTCWKTNPQVIEG
DDQDRSKDETEWERKNKPLIAGGRFWRAVSEADRRRLVGRYSGLLLHIRDSQPWDRPVTGKVNGLAKVTPAWAGCLKPKTFD
EKQDSETYGQPTMWEYTEASQAGRPGLVRDIHPDRVFILGDWTGDAIGFLEPAYNSFISLEKVEGGSGESFLKNAARQLLLNFDK
EIDLNNIASMYGVSLDQLNQRFNDATRQLNRGNDVMLPTQGATATQLVSAVSDPGPTYNVNLQTAAAGVDIPTKILVGMQTGER
ASSEDQKYHNARCQARRVQELTFEINDLFGHLMRIGVVPLKAEFTAIWDDLTVPTKAERLANSKTMSEINSAAIGTGEPVFTAEEI
REEAGYDPLVGGDPLPDT

2539815..2540666 PHAGE_Pseudo_phi297_NC_016762: putative head morphogenesis protein; PP_02430; phage 0.0 - NONE NONE NONE
MREFDRRMRKIRDGYVAALDRIPAQPVVNEQYTYRLDQALLSAIFADTNLMVDEILQEGGERDLWFFESYVGVAYIRGTAQTHAN
LAQQSPAYRAGRESLDVLLRSDAYRARMALLRAREFEEMKGLSGQVKADMARILAEGMGRGKNPREIARDLTAQTGIEARRGH
RIARTEVTTALRRARWDEKDAAEADYGVQSKLMHMSALSPSTRATHAARHARLYTSDEVRDWYSRDGNSINCKCGQVEVLVDD
EGNPVVPAIVERARRNYQVMKAKGRGPWAKED

2540670..2541947 PHAGE_Pseudo_JBD44_NC_030929: hypothetical protein; PP_02431; phage 0.0 - NONE NONE NONE
MPMQVNITTQVNSASIRRETHNGREHLVLPSYTLPAGVIMNGGLYTAEQIDKHYPGLEGTLAPLGHPMVDGKFVSAFSPEGINAA
HVGAWNRNVKKSGNRVYMEKWVDVEFAKSTEGGRELLQRVEALEKGEDAPPIHTSVAAFLNRIEPNESQRAQGAEWVADIQSM
DHDAILLHEVGAATPEQGVGLMVNADQAVPLQPNSGALVGESYREREQRLDRAAKERFASGPDQYAWVADFTDSQAVISRNGG
VTEVYGYKLEAGKIVFDESGQPVVRQESWVAMVANSIKNIFTHRQARPDQPEKEGDMPLTPEEKAEIVKEIGTNTSSAIKELADTII
KPLADKVDGLVANHKALADTLTANQRAEEDSMREAVKAKFGEVIANSLAGDALKEMFKQCGESAPLGANAATDKGGLTADIANL
PKE

2541951..2542400 PHAGE_Pseudo_JBD44_NC_030929: hypothetical protein; PP_02432; phage 5.02e-104 - NONE NONE NONE
MSRYRRVNIDGKSLFKTETRKTAAELLPGTFAVINGSDLFAQASASVGRLYVIDCAHHEGLNIRDEVPAGHSAVGNYVEEGRELA
VLCPAGTYKKDTPIKLGTSGQGAIASSDTDTVLGYSQDDAVIASGETDFIRIRFRVGSVAAPAP

2542416..2543510 PHAGE_Pseudo_phi297_NC_016762: putative coat protein; PP_02433; phage 0.0 - NONE NONE NONE
MFLTQQAIAAHPRLMGHYQELQANRNIWNNQNAAMITHHRGAMTPEMLACNALAGLGREFWAEVDAQIIQYRNQETGMEIVNDL
LQVQTVLPIGKTAKLYNVAGDIADDVSVSIDGQAPYSFDHTEYNSDGDPIPVFTAGYGVNWRHAAGMNTVGIDLVLDSQAAKLRK
FSKRIVAYTLDGATNIQVENYPAQGLRNHRNTIKVNLGSGAGGANIDLTTATPQQIIDFFTKGAFGQAARANKVDAYDVLWVSPEI
NANLSQPYMITMGGGANAVVAGTVLDAVMRFIPARAVRQTFALSGNEFLGYQRQRDVVTPLVGMATGVVPLPRPLPQVNYNFQI
MSAMGIQVKKDDEGLSGVIYGANLA

2543521..2543979 PHAGE_Pseudo_JBD44_NC_030929: hypothetical protein; PP_02434; phage 1.46e-79 - NONE NONE NONE
MPKYEVIKPWNGVSKGQVLELESLAAALLPNVREVGALKNGSLTLDVSAQVDEAARQALAEARASVDAMIDEAKAQAEGIIAAAN
AEAASIREQAKAQAGTLTPAIPDGSERRELIKARLKELKIEFDGRQGEEALAALLPEGELAKLFPAK

2544291..2544692 PHAGE_Pseudo_YMC11/07/P54_PAE_BP_NC_030909: hypothetical protein; PP_02435; phage 1.02e-91 - NONE NONE NONE
MITFEQARQYLQSQGIDNVPDFILAAWIEQLQQIQDCLDAHYPASTSLLIQAYLLALFALAQADKYISSQTAPSGASRSFRYQAFAD
RWKAQLALLNALDKYGCATGLIPPNPTQTAHGGLWIARGGCMCGDS

2544689..2545009 PHAGE_Pseudo_YMC11/07/P54_PAE_BP_NC_030909: hypothetical protein; PP_02436; phage 4.63e-72 - NONE NONE NONE
MSTTANWSYTNTATVRPFLHFDLSTQEAVYGPEYEIACTWVAKGEQVRDNSGAEFVSRHQIFTEDRRPKYLDLIQFDGSNGWEE
IRSVTNWDMSFFGEQPDFLLVT

2545011..2545415 PHAGE_Pseudo_phi297_NC_016762: hypothetical protein; PP_02437; phage 3.19e-92 - NONE NONE NONE
MAIQGIDRVRRNLRVAVENIAGGVSERAVYEVLSQGAAMAQTMTPIDTSTLVNSQTAPQITVGPNGVEGSVGYTAAYAAAVHEAP
GTLAGQPRDENDPSRGDYWAPNAEPEFLTKGFDQIIPAIPAILRRTYRV

2545412..2545786 PHAGE_Pseudo_JBD44_NC_030929: hypothetical protein; PP_02438; phage 1.06e-86 - NONE NONE NONE
MTPYDAFQDWLASILGEGYQYSRGMWVDHPSLDSAFIAAIQQTGGPPTQVDVRRLRFKVILLGPKGVRKHVVDVGNSIETLAQA
ALGDSVPCGAASVRAIGEPIGPGYTTENRAWYSLDLEVLY

2545801..2546796 PHAGE_Pseudo_JBD44_NC_030929: hypothetical protein; PP_02439; phage 0.0 - NONE NONE NONE
MACKKLKFPGRDVVLEYYIGCGDALPAENDWRRFGSLRTKEFTVEWDTIDATDSDSVGALRENLASFQTLTISGDGTVKASGAG
AQNLIDLTKHVVKPDSTGGQPVVWMRMTFPDLTFTAFMLISNLSRSAPYDDVTTYSFEASATASDFGLIVEDTPDADAPDPTSIQV
VPETLSLTVGEGFNFEGVVLPVGAPQGLRWTSTAPTVAAVNAVTGEVSALSAGSATITAASSVVPGVTDTATVTVIPLVQGITVSP
TSVSIAEGATQQLTAAVSPTGAAPGLVYESAAPAIATVSSSGLVTGVDVGTTTVKITSAARPSVSVTVPVTITAP

2546793..2547410 PHAGE_Pseudo_JBD44_NC_030929: hypothetical protein; PP_02440; phage 3.42e-149 - NONE NONE NONE
MILTEIGEIGVHTASGECFLLRPSLYAMTQLGTPAEIVDVFARVMSDPITEKHQADQFADALAVVVACSEQDLSDVFGYYDQDLVY
RPGTADVEHLVPLARCLLKHGVTGALPPLPRRHDEEPNYSGEFVAREYVATAIAHLGLSEREAWSMTMTGLIGALRAKYPPTESN
APGARAPTAAEHDATMEWFDKIEAKRKARAKGAP

2547410..2550421 PHAGE_Pseudo_phi297_NC_016762: tail tape measure protein; PP_02441; phage 0.0 - NONE NONE NONE
MAENVGSIYYTVEADTSGLVNGTNAADRSLDQMQATMRRADSEAARLNTTVTKLSSAIKTIIAASALREMASMVQSYQEMADRV
RLASASQEEYENVQARLLRTANGTYRALSEAQELYIRTSAGLKALGYDTTSALDVMDSLSYAFVTNATKADAAEAAISQFSKAINT
GKVSADQWETISSAVPSVIEDIGAAAGKTGAEVRSLGAQGQLTAQMLTEGLRKSLEENSKAAAGMSNNLTDAGVRIRTAFTQVLV
SLEDQTGALQTFTNGLISAADALLEFGLDSEKMAAFLDTATVAAASLASVVAGRLVTSLYAAGAAQVQRLRATLEQIAADRNAAIG
ALRRAEAEKAAAAAAVALAQADLNAARGSNAHATALNALLAAKERDLAATRALTAAQATLNGVATTGTVVMGGLRSAMAFLGGP
LGVVLLAATAIATFATNAREAKEPTDLLTLSVEKLGQAQLKVAQLDIDKRIQAVSDKLKLLGENYAFAAKEAQGSGRRANRYAEDA
VRIQGAVEELTQELDQLQKKRSDVDAALDKKSSSPSGNGPDRQANPEDTKALQNLRDEAELSALAGEERAKLAARKKLSADATK
EEIAEAERLAVQIFRNSEARKQEKKSASDTASTVKKSMEDQRRAALDNEKTIGDLSQQLAQAGLKGKELAEAGAQSRLNPFATPE
QVAQVRALAAALYEAQQVEANKQLLGQMDPIAGEDQRYQTELENLKKLNEAKLLEDQRYLELKAQAEQQHDATMKQLEEERFR
RQAAGNEMIMATLDQVQQAGTNALTGLITGANNGADAMRQLAGAMLNQVVGALVKVGIEQAKNFIMGQAQQAAAATTAAATGA
AMASAYAPAAAAASVASFGGAATAGLTAMAAAIPAMLGMFGGGRQYGGPVGAGGMYRINENGAPEVFQAANGRQYMLPNTRG
EVISNGDATAQGSPQISLQIINNGPPVSATATMDGNNLRVTLDAVEQDFANKVSSGQGLYPKAIEGAYGFKRAGR

2550418..2550885 PHAGE_Pseudo_phi297_NC_016762: hypothetical protein; PP_02442; phage 1.96e-111 - NONE NONE NONE
MIKWPDGLPFPLREGYGFKTVEPMARTSLQSGRARYRRNFSNVPVALEVSWLFTAEQARLFKGWYRDVLKDGVKWFECDLRT
EEGIVPCNLHFEGIYDGGYLVGRDHWRFNATVVMRERSIIDPGWAEILPEYILLADIFDIAMNREWPRHGDGS

2550869..2551360 PHAGE_Pseudo_PMG1_NC_016765: hypothetical protein; PP_02443; phage 1.31e-115 - NONE NONE NONE
MATALERFYASDGPDIPIATIEITRPSRPDPILICQGFKDLTCMTEDGRLLTFIAGAIDVSIPKRDNSGNQNVGFAIDNVTGFAQQYIA
EAIDAGEPVTLVLRIYLGSDLTAPAERPYRMRVKGVDFESLTVQVEAGYYDLINTAALRHIYNVSEFPGLKYWP

2551365..2551772 PHAGE_Pseudo_YMC11/07/P54_PAE_BP_NC_030909: hypothetical protein; PP_02444; phage 3.14e-92 - NONE NONE NONE
MPNRYLTAIYTEGGRALPCLDCWGLTLIARVELFGLPMLTDFGGVTRRTPVSMQRACDAEIHRALEQCEPGPGVIAAAYRGRLLD
HVGLLVEVDGRLRVLEINPGSGVSLTPLQKFSDKYSKVVFYRDRNLPIAP

2551744..2554473 PHAGE_Pseudo_PMG1_NC_016765: putative tail protein; PP_02445; phage 0.0 - NONE NONE NONE
MIEIYPSLLDGEPLERHPIGRRMTIHAWLTANSPGYRCHDVHPFSIGVVPAEVALCDDLTDKQKKAHEDFIHPGEWAERIIDRGDIV
RIYKLPRGTDPFTITAALFKGAQSVFRMLMPQLPGMPTNPGQGASLSETSARGNKVKLGDAIREVAGRRLIYPDYILPPRKYFAGP
REQWTEMLLCIGRGRFQIAEGAAKIGDTSFLALGADASFQIFEPGQNVSGHPASVWWHLVEEVGASSTGNAGLDLTESSNLTPN
PSATTFTFSGTNIIISAGAGSFPSDWVAGTILRVEAMYPYSVNDGGGTNRDVVTGDIAQLGLDVGDEIEVVGTNGGLYLVNDITST
SMTLNYSNGSPANALQTGSGNAAIGPRGLRYRITAYSAQQLTVERLTSAGGVDVDWPGFTALNSSTSRVTIDPTSLEGGWRGPF
PACPVSEKTNFVEIDVFCPEGLCGVGREGGQIYQIRTYYDIQWRDMAIGGAWTTVSKNHAGSSLDQQGFTDGIPLPYMMRPEFRIR
KVFVNQGGNSTSEYRDRTQWYGMRARLQAPSSYAGVTTMAVRYRSSDRIAAQTESRVSVEATRMLTTRQNGAWTSEIATRDIV
PFLCYIAKERGYTDADLDLEELDRLDAIWKARGDKFDMIYEDGKVTVAQIMDDVLAAGYAEKTIKRGVISAARDEPRTTFGHMYSP
QNMDGPLKISISAPSEDDYDGVDVEFVNANGWIEDTVQCRLPGDVGRKVEKITAVGVTDRNRAWRYGMRRRMAQRYRRTEYS
FDTGLDALNSDFWDYVALAGDVPGPGLAQSAYLKSFVISGSSVLIESSEPLDWSLLNSPALYLRRPDGTVSGGYPASRIDDYRLSI
PSIDFIPDVSWEIEPPHLLLGNPYPALISSIDPNGNTSASVRAVNYDPRVYTYDNASAPN

2554534..2556213 PHAGE_Pseudo_O4_NC_031274: putative pectate lyase; PP_02446; phage 0.0 - NONE NONE NONE
MTTYATGNPLGSKDPRDLYDNAENFDTAMNDRENLAWSDRFGVSRKTWFGLEQQVADFLAAQGYEPVPLEYVDGSPLTVDRP
TQLIERDGNLYSVKLPASFPVELTGNWATDQNLLVAQVDRSLRQQLRDSGGSGMLGFNASESYPSDTIGYEVNTLMALKVVVVT
NYGATGNGTTDDTAAIQAAIAAAGPYSDVVFPSGTYLITSTLTSLTGQRWLGRGGQRGTTIKKGANIDMVVVGTLSTILDINLEGV
GATYTGKGFRIVSGFSQTITRCRAVNMGGEPLYFDSNAGGGANVTVFEGYPVDTDAYAGCAIAGDTAPHPRFFRGMWLSGANF
ALGPGAGNGGSMTEFYIRDLRFDATSTLFHISNGRCATLGATTTLKGFDHSIDGVAFAGPVALDSAQGINLGPSCSVPSLTENATN
SQYNSVYVQRRTYTPTWTQTSATPAIGNGTLTGNYVRAGHMCHVQIELVAGSTTTFGDAASGYRFSLPFPGHLSFNQRGFPVRI
YDTSAGADFTGWASIGAGQGYITISVGAQQVRATSPMTWANGDTLQCSFSYMTR

2556322..2558301 PHAGE_Pseudo_PAJU2_NC_011373: putative acetyl transferase; PP_02447; phage 1.27e-62 - NONE GO:0016747 - acyltransferase activity, transferring groups other than amino-acyl groups [Evidence IEA]; NONE
MSVLLFHAGFSSLAGGFVGVDVFFVISGFLITQLIYKEISTAGTFDYHRFYSRRVRRLFPALFVTVLVSFICANLFFSPEHLSRFSGE
VIYSLFSLSNFYFWSESGYFNTASDFKPLLHTWSLSVEEQFYIFWPILVVFFGKKFGSKGVVSFLLISGIASLLGNVSFIDGSSVLVS
WAGKVVSGWFSDGASTIFYLTPFRVFEFCLGAIIVFLPKVNSSSVHNFLFASGVALIGYSVFEFNALTPFPTYNALIPCAGSALVIYS
SGSYFSRLTISTSPFVFLGKISYSIYLVHWPIIVFYKYYYSGDVSISAKVAIVIVSVVLGYLLFRFVETPFRSQSGKTISSNGFNLSCLM
LSCLLVVPSATAWGNSGWTWRVSEPPKGIAAQLADSKKFHIDQYGGNGYQERGWISGGGIADVVVIGDSHARQYAYGLDQVLG
TPEKLNIYLSSVSCILLPGMTRLTPGTDWDSLCSAALDDALAALDRNPKAVLVIAQLWVDQLTIAATNPGHVPVPDSKGAGGYSLLI
EKIRELKSRIGSRKMIVIGNVPGAGSPDIAGCYNRPSFARGYCLSKIGIPYSDVRSVAINKALADVSKIPGVLFINPHDVFCHDGFCK
SIANDAILYSDSNHLSKAGSEYFVSKEKDNILTHIKRPPELSLSKGS

2558346..2558975 PHAGE_Pseudo_JBD44_NC_030929: lytic enzyme; PP_02448; phage 5.02e-141 - NONE NONE NONE
MPITEQQLLHVLPNAGPRAGVFVGALNRGMTRFGITSPVRVAAFLAQVGHESGQLTRLVENLNYSAQGLAATWPSRYLGADGQPNALAQRLARNSRAIANNAYASRNGNGDEASGDGWRYRGRGLLQITGRANYRAAGAGLGQPLEQEPELLEQPEWAAISAAWWWASHGLNDLADRGEFAAITRRINGGTNGQAERLALWERAKAVLS

2558972..2559340 PHAGE_Pseudo_PMG1_NC_016765: hypothetical protein; PP_02449; phage 6.56e-60 - NONE NONE NONE
MISARVISIALACLVLVGLGTAGGVWIGARHYRPQLDAALADLAACRSARGSLEAAVVEQGGQIAALRQAGEQRARDAAQAVDRGRQQAAEQYAAAQRLLRERSAGDQCLAAEVVIDQELGL

2559337..2559600 PHAGE_Pseudo_PMG1_NC_016765: hypothetical protein; PP_02450; phage 3.60e-49 - NONE NONE NONE
MRVVLMLVMVALAGCAGRQEAEPRTVRVEVPVAVPCRVPAVEVPAWAAAGLKKSDDLQTKVRALLAERRQRIGYEAQLLAANQACQD

2559636..2559899 PHAGE_Pseudo_YMC11/02/R656_NC_028657: hypothetical protein; PP_02451; phage 5.00e-56 - NONE NONE NONE
MLVIRFKGWSVKLDHQVGSAGKHGIWSFHGSESSYVPDMETILRHAAIRPAEPKEGGEVEVFICDSRMPQDEWRAVGTGVAAYEAER

complement(2559903..2560415) PHAGE_Pseudo_H66_NC_042342: hypothetical protein; PP_02452; phage 4.09e-113 - NONE NONE NONE
MSYSDPRHCHHQRVTQWLAAMRQHAAWLYAADEQYLYLVAEANELYQCGIVGLQDRHDMVTDALGMYSWAIEHGITRETHYCADCCYDVLDGGRAVGTVDSEGIYHGPAPARQRLGYISRDPLDGITYLRLGQALERAGVVRGLEIELDAGGTLQLAEQIPDDFRPWRWA

2560649..2560663 attR N/A
CTTGAAAACCGTCGA

complement(2560841..2562424) rhodanese-related sulfurtransferase; PP_02453 N/A - NONE GO:0016783 - sulfurtransferase activity [Evidence IEA]; NONE
MSQIAVRTFHDIRAALLARRELALLDVREEDPFAQAHPLFAANLPLSRLELEIHARVPRRDTPITVVYDDGEGLAPVAAQRLLDLGYSDVALLDGGLSGWRNAGGELFRDVNVPSKAFGELVEAERHTPSLAAEEVQALLDARADAVILDARRFDEYQTMSIPGGISVPGAELVLRVAELAPDPRTRVIVNCAGRTRSIIGTQSLLNAGIPNPVAALRNGTIGWTLAGQQLEHGQTRRFGAISQDTRKAAAQRARAVADRAGVERLDLAGLAQWQDEHDRTTYLLDVRTPEEYEAGHLPGSRSTPGGQLVQETDHVASVRGARLVLVDDDGVRANMSASWLAQMGWQVAVLDGLSDADLSERGAWSAPLPRQPRADTTDPTTLADWLGEPGTRVLDFTASANYAKRHIPGAAWVLRSQLKQALERLGTAERYVLTCGSSLLARFAVAEVQALSGKPVFLLDGGTSAWVAAGLPTEDGESLLASPRIDRYRRPYEGTDNPREAMQGYLDWEFGLVEQLGRDGTHGFFVI

complement(2562421..2563026) 3-mercaptopropionate dioxygenase; PP_02454 N/A - NONE NONE NONE
MSSILRLDRLRQFIGELATLLDSRPDESTLLAQAHPLLAELVRQDDWLPEDCARPDPQRYQQYLLHVDSRQRFSVVSFVWGPGQITPVHDHRVWGLIGMLRGAEYSQPYAFDAGGRPHPSGARRRLEPGEVEALSPRIGDVHQVSNAFSDRTSISIHVYGANIGAVRRAVFSAEGEEKPFISGYSNSRLPNIWDLSKENPA

2563139..2564035 PHAGE_Klebsi_ST437_OXA245phi4.1_NC_049448: LysR family transcriptional regulator; PP_02455; phage 6.98e-11 - NONE GO:0003700 - DNA-binding transcription factor activity [Evidence IEA]; GO:0006355 - regulation of DNA-templated transcription [Evidence IEA];
MKIDDIDAFVAVIRNASLSQAAESLGLTQSAITRRVQSLEESLGVALLDRNTKPLKPTASGLRVYEQCRRVLREVDGLRELVAGDATPSGVLRLGVPQSIGEVVLLDALRRLADEYPELRAQVGTGWGSHLLARLENAELDAAVVLFPPSKVFPEELGATPLGRMELCVVVARDSTIQARRLLDCYHHGWVLNPDGCGFRAGLQRALADQGLGLQLNLETFGSELQLGLVAAGRGLGLVPAPALARSRYRDQLQVLQLEDFQPLIQLWLVRPRLLGNLETPARLFGRAVAEGLDMQAG

#### region 5 ####

5102870..5102881 attL N/A
CGCCCGCTCCAG

5102938..5102951 attL N/A
TTGGGGGCCGTTTC

complement(5104641..5105696) PHAGE_Pseudo_Dobby_NC_048109: portal protein; PP_04774; phage 0.0 - NONE NONE NONE
MSKRRSHRRQQPVTVQSAQEGEFIPRQGGRAEAFTFGDPMPVLDGRGILDYLECWSNGRWYEPPLSMEGLAKAVGSSVYLQSGLKFKRNMLAKTFIPHRLLSRATFEQFSLDWLTFGSAYLEQPRSRLGTRMPLQAPLAKYMRRGTDLETFYQVRSWKDEHEFEKGSVIQLREADINQEIYGVPEWFCALQSALLNESATLFRRKYYNNGSHAGFILYMTDAAQNEEDIDALRTALKTAKGPGNFRNLFVYAPNGKKEGIQLIPVSEVAAKDEFGSIKNISRDDQLAGLRVYPQLMGVVPQNAGGFGSISDAAAVWASLELEPMQARLQQVNELIGEEVVRFTALGLPSLR

complement(5105696..5107456) PHAGE_Pseudo_Dobby_NC_048109: terminase large subunit; PP_04775; phage 0.0 - NONE NONE NONE

MNAAVEIPIRDNRRQAKFLYWMGWRVCDIADHLGEKDKTLHSWKDRDGWDRADSVERIGGALEARLVQLILKDGKTGGDYKEID
LLHRQLERQARIQRYQGGGTETDLNPELAKRNEGPKRKPKRNDISEELTEKLVEAFLDGCFDYQKDWYRAGNQRTRVILKSRQI
GATFYFAREALIDALETGRNQIFLSASKAQAHIFKAYIQAFARDAVGVELKGDPIILPNGAELHFLGTNARTAQGYHGNFYFDEFFW
TFKFKELNKVASGMAMQKRYRRTYFSTPSSMAHEAYTFWTGERFNKGKPAADRIKIDVGHDALQQGRLCEDRIWRQIVTILDAE
ARGCDLFDIDELRLEYDAEAFQNLLMCQFVDDGASIFPLTMLQPCMVDSWDLWSEDYKPFALRPFGDRQVWLGYDPAETGDTA
GLVVVAPPAVPGGKFRVLERHQFRGKDFAEQAEFIRKVTQRYWVTYIGVDTTGMGSGVAQLVRQFFPGVRTFSYSPEVKTQLV
MKAWSVIKNGRLEFDAGWTDLAQALMAIRKTITAGGRQFTYTAGRNDNTGHADLAWALFHALQNEPLEGQTPANTGRMEIF

5107612..5108433 PHAGE_Pseudo_Dobby_NC_048109: GPO family capsid scaffolding protein; PP_04776; phage
0.0 - NONE NONE NONE
MKKFRSKWFRIAVEGATTDGRNIERDWIEQMAAQYDPNTYGARINCEHIKWAWPAGEFGAYGDVLACKAEEIDINGQKKLALFA
QLEPNQALLELNKQRQKVYTSVEIDPKFADTGKAYLVGLAITDSPASLGTEALSFSAKNGTLASRKTNPDTLFSAAEEGTLEFEEY
EDKPSVGAALFTKVKELLKGKEARTQAEFGQVGEAVEAIAEHSRDLGEQLGEQKTQTQQLASQLDKVTKELADLKSTLDSTRDH
SQQQRPPVTGGGSVALTDC

5108469..5109485 PHAGE_Pseudo_Dobby_NC_048109: major capsid protein, P2 family; PP_04777; phage 0.0
- GO:0019028 - viral capsid [Evidence IEA]; GO:0005198 - structural molecule activity [Evidence IEA];
GO:0019069 - viral capsid assembly [Evidence IEA];
MRNETRKQFDAYLAQLAKLNGVNSAVQTFAVEPSVQQKLEQRIQESSEFLKQINVYGVDELQGEKIGIGVSGTIASRTDTTGDGV
RKPRDVSALDNQRYECKHTDFDTAITYAMLDAWAKFPEFQALLRDAILKRQALDRLMIGFNGTSAAATTNRAANPLLQDVNIGWF
QQYRNNAPARVLKEGKAAGKVVVGNGADADYKNLDALVFDVVSSLIDPWHRRDPGLVVILGRELVHDKYFPMVNKDQPATEKIA
TDLILSQKRMGGLPPVEVPYVPEKGLMVTTLKNLSLYWQIGGRRRYLKEVPEKNRIENYESSNDAYVVEDYGLGCVVENIEVAE

5109491..5110192 PHAGE_Pseudo_Dobby_NC_048109: terminase endonuclease subunit; PP_04778; phage
6.99e-167 - NONE NONE NONE
MAFSPAKAHFLRVTAAQEAAATAPHQGMEGANAYELQLAQLYQDRSRLKNIQSGEGKAALKVELLPAYQPYISGVLQAGKGAQD
EVVTTVMLWRIDAGDYAGALDIADYVLAHDLVMPDRFARTAGCVIAEEIAEAALKAQKTGGSFDLATLHRTLLLTDQADMPDEAR
AKLYLAAGHATLEGLSVESPGQPGQVQAGIDLLKRAIQLHDKCGGKKDLEAAERLQKKLTASGG

5110296..5110757 PHAGE_Pseudo_Dobby_NC_048109: head completion/stabilization protein; PP_04779; phage
2.35e-106 - NONE NONE NONE
MSGFIANGPVPSGHINSDPFWPTIELEHVRANLRIDSSVDPARLEVAVIAAVISVNRELRAWRLEKSAAGYAELAQVPSDKVRDTS
ELVQLYLRAVQSATAAEVAERYRWYDTTTNGNDKAQDIATTIDDYRRDQRWAIRDFLKRPRTTVELI

5110757..5110969 PHAGE_Pseudo_Dobby_NC_048109: tail protein X; PP_04780; phage 1.10e-45
- NONE NONE NONE
MAAVAIAHQNDTVEALCWRHYGRTAGVTEAVLEANHGLADHGPTLPPGLKVTMPDIPTAAPERQMVNLWD

5110994..5111347 PHAGE_Pseudo_Dobby_NC_048109: membrane protein; PP_04781; phage 6.33e-
76 - NONE NONE NONE
MADLTTTATAGAIMGLGLGVTLPVDGGMLFGALLGAWLATGTKQDLKAWSRLLSLILPTCVGYLFADVALARVPWLTNLAFSAFV
CALVVIPLSLKAVAWVDKVDFDDLWRRIRGGR

5111349..5111621 PHAGE_Pseudo_Dobby_NC_048109: holin family protein; PP_04782; phage 4.12e-
54 - NONE NONE NONE
MLMTAVPLIAALAYIAAALRLVCYQRCGARFRRSVSLLASLLGASMAICGLEILLYRPPVSIWHAIVAALLCLLIFRSRGNVAALLRPS
A

5111618..5112424 PHAGE_Pseudo_Dobby_NC_048109: DUF3380 domain-containing protein; PP_04783; phage
0.0 - NONE NONE NONE
MTLRYGDRSQEVRQLQRRLNTWAGANLYEDGHFGAATEDAVRAFQRSHGLVADGIAGPKTLSALGGADCSHLLQNADLVAAG
ARLGLPLATIYAVNQVESNGQGFLDNGKPAILFERHIMYRRLAAHDQVTADQLAAQFPALVNPRPGGYAGGTAEHQRLANARQID
DTAALESASWGAFQIMGFHWQRLGYVSVQAFAESMGRSESAQFEAFVRFIDTDPALHKALKARKWTDFARLYNGPDYKRNLYD
VKLEHAYKQHTEAHKETT

5112421..5112663 hypothetical protein; PP_04784 N/A -
NONE NONE NONE
MRTGAQSGEHFPYKELLERMTKLSPTGCVAVVLPRDTPMEDCQKMADALKHVMAVPPLVICGDVQSLDEAAMNAAGWYRK

5112660..5113022 PHAGE_Pseudo_phiCTX_NC_003278: predicted lysis; PP_04785; phage 6.82e-50
lysB NONE NONE NONE
MTTLRQALYGAALLSALGLLLWVQQQRIDLAQARLAQAELARKASDAQLSRQADTITALEAALSRERQAQADLDQQRQQLRQAL
AIRERLIEDLKRDDEPYRQWADQPLPDVARRLQQRPA

5112946..5113215 PHAGE_Pseudo_phiCTX_NC_003278: hypothetical protein; PP_04786; phage 9.86e-
58 lysC NONE NONE NONE
MTMNPIASGLTSLCLMLLAGCNSAPLSPERPLTISGCPAVTPCSLESAAPKDNGGLQTEVERIGLAWAECAAKVDMIIRTQGAPHE
QAR

5113199..5113735 PHAGE_Pseudo_Dobby_NC_048109: tail protein; PP_04787; phage 2.33e-126
- NONE NONE NONE
MNKPDSLKAHLLAAVPELRNNGDRLVIFIDNGRVRSTSAESLSFEYAYDLQVILTDFAGHPDSVFLPLLGWLLVNQSDLLANLTKV
QDGITFEADMLDRSKVDLGIVLPLTERVVVKRREDGRYDVSHPEEPQLTEAIEVDGPMQMLANGELLAEWTPPTPTEAVMLETP
QIRRPANG

5113728..5114186 PHAGE_Pseudo_Dobby_NC_048109: virion morphogenesis protein; PP_04788; phage 5.72e-93 - GO:0044423 - virion component [Evidence IEA]; GO:0005198 - structural molecule activity [Evidence IEA]; GO:0016032 - viral process [Evidence IEA];
MADSLEALEDWAGPILRALEPGPRAALARSLARDLRRSQQKRVMAQRNPDGNAYEPRKKRELRGKQGRIRRKIKMFQKLRTVR
YLRAKGDAQAITVSFAGRVTRIARVHQYGLKDRAERGAPEVRYAQRRLLGFTEADLEMIREGLLAHIPA

5114256..5114828 PHAGE_Pseudo_Dobby_NC_048109: baseplate assembly protein V; PP_04789; phage 2.84e-134 - NONE NONE NONE
MNDFAALSRMIENLIRLGTIAAVDHAAQRVRVLTGDLLTGWLPWASPRAGADREWNAPTLDEQVLLFSPSGQTANGVVLTGLFS
DLIPPNGDRDALHRTTYRDGAVIEYDSAAHHLRAVLPAGGTTELISDGGIRIVGDITHQGDYIQTGNQTVTGKVTVSVDVIAKGISLV
GHTHGGVMPGGATTGKPQ

5114825..5115169 PHAGE_Pseudo_phiCTX_NC_003278: baseplate wedge subunit; PP_04790; phage 2.80e-76 - NONE NONE NONE
MNAHTGGAIDRAAHIRQSIADILTTRIGTRVMRREYGSQLPELIDAPFNDTTRLQVYAATAMALMRWEPRIRLSRVQITGQNLAGQ
VLMEIDATLVDSNEPHNLSIPLQMGASA

5115166..5116080 PHAGE_Pseudo_phiCTX_NC_003278: baseplate assembly protein; PP_04791; phage 0.0 - NONE NONE NONE
MTTNFVAIDLSQLPPPHAVEQLDYEQILAERKAYAISLWPEDQQAEIAARLALESEPLTKLLEENAYREMLWRQRVNEAALANMLA
SAQGADLDQLAANYNVKRLVIQPGDPSKVPPVQELLESDDSLRERAQMAWEGLSTAGPRNSYIFHARAADGRVGDASAVSPSP
AVVVVTVQAAQGNGSAPADLLDIVDAYLNDADRRPVADRLTVQSAQVLEYRVDATLYLATIGPESEPILDAARARLTAYVHQRRR
LGMEVSESAVHAALHVEGVRKVTLSNWSDIAATPAQAPYCTGITLTLGDE

5116080..5116616 PHAGE_Pseudo_Dobby_NC_048109: tail protein I; PP_04792; phage 1.27e-68 - GO:0044423 - virion component [Evidence IEA]; GO:0005198 - structural molecule activity [Evidence IEA]; GO:0016032 - viral process [Evidence IEA];
MPSLLPRNATELERLAAEALAQIERVPIPLRQLWNPSTCPVALLPYLAWAFSVDRWDSTWPERVKRQVIRDAYLVHSHKGTLSAL
RRVVEPVGSLTDILEWWQQTPTGVPGTFEITVDVSDNGLDEETVLELERLLNDVRPVSRHLTRLDLRITPDILARHGLATIDGDTLE
ISPWKQ

5116618..5119008 PHAGE_Pseudo_Dobby_NC_048109: tail fiber protein; PP_04793; phage 8.76e-41 - NONE NONE NONE
MTTPKYGGLLTDIGAAALIAASEAGKKWQPTHMLIGDAGGAPGETADPIPSAAQTKLIRQRYRAQLNRLFVSEQSANVLVAELVLP
MAIGGFWIREIGLEDADGKFVAVANCPPSFKASVESGSARTQTIRVQIILSGMEHVELIIDDGIVYATQDWVTAKVAADFKGRKVLA
GNGLVGGGDLSADRTIALPASGVGAGTYRAVTVNANGIVTAGSNPTTLGGYGITDALHASEAVTTPTANKLLRLNAAGLLPASITG
NAATASRLAAPITLSASGDATWSARFDGATNVNGVLTLANSGVTAGTYAKVTVNAKGLVTGATGLVASDIPALDAGKITSGILPAA
RGGTGNGIGQAATAVKLAAPRTIYLGGDASGSTTFDGSSNAGITVTLANSGVSAGSYPKVTVNAKGLVTGGGGLTAADIPALDAS
KIATGRLDLERLPLVSQGLATAVHTSVDPNSVVIPLVLTNHANGPVAGRYYYIQTMFYPSVEGNATQIATGYAGVADMYVRYAYG
SPATTDPSKREWSAWVRCDLGGAFAHAPDGVLGGGVNLDSMIASGWWHQPFSANAQNGANYPVGEAGILTVHAPTSSMIYQT
YRGYAAGGLYWRCRYNGTWGGWFRAWDSGNFNPSNYLAKSEYSWASLPGKPATFPPTAHIHDASQITSGILPLARGGLGANN
ATTARSNIGAGAIATASRGGNGWFKDNDTGLIFQWLHLPVGDHPGGFLDKVVTFPTPFPNACLHVIPTVRELGRPATSASTVTVA
EKAMSTTSVTIVTTEYISTVQNFGINVFAIGY

5119060..5119521 PHAGE_Pseudo_Dobby_NC_048109: tail protein; PP_04794; phage 1.69e-40 - NONE NONE NONE
MTDYMFSPSIVAFYPVSMREAYEVSGSWPEDGILVSEEVHSRIMDEQSAGRIICAGPDGQPMTKEPPPPTVEEQATRERVWRN
QQLKDTDTLIMRHRDELEFGTTTLSAEQYQALQVYRRQLRDWPELGAFPLAEHRPTAPDWLYHQIEDGVL

5119612..5120787 PHAGE_Pseudo_Dobby_NC_048109: tail sheath protein; PP_04795; phage 0.0 - NONE NONE NONE
MAADQYHHGVRVQEINDGTRPIRTIATAIIGLVATAEDADATAFPLDTPVLITNVQAAIGKAGTSGTLPASLQAIADQANAATVVVRV
KPGEDEAATNSAVIGGVSADGKYTGMKALLAAKARLGVVPRILGAPGLDTQPVATALIAIAQQLRGFAYVSANGCKTKEEATAYR
ENFAAREAMVIWPDFLTWSTVVNQTVPAPAVAQALGLRARIDQEVGWHKTLSNVAVNGVTGISADVFWDLQSPSTDANYLNEN
EVTTLVQEGGFRFWGSRTCSDDPLFAFENYTRTAQVLADTIAEAHMWAVDKPMHPSLVRDILEGVNAKFRELKGLGLIIDAQAWY
DPSMNDKDTLKAGKLRIAYDYTPVPPLEDLTFFQKITDSYLVDFASRVNA

5120844..5121359 PHAGE_Pseudo_Dobby_NC_048109: major tail tube protein; PP_04796; phage 1.69e-124 - GO:0044423 - virion component [Evidence IEA]; GO:0005198 - structural molecule activity [Evidence IEA]; GO:0016032 - viral process [Evidence IEA];
MAMPRKLKNMNLFNDGGSYQGLVKSCTLPPLARKMEAFRGGGMNGPVKADLGHDDDGIQFEWTVGGLELTVLKQYGAVSASG
VMLRFAGAYQQDDTGAVTSVEIVVRGRHETIEMGDAQPGEDTEHKITTTCSYYKLVVNGEEVIEIDLLNFVEKVNGKDLLEAQRKA
IGL

5121414..5121743 PHAGE_Pseudo_Dobby_NC_048109: tail assembly protein; PP_04797; phage 5.03e-70 - NONE NONE GO:0098003 - viral tail assembly [Evidence IEA];
MKTEQTPADLQNAPDNVVTLDQPIKRGAQSIESLTLRKPSSGELRGLHLLDLLQFDVTATMKILPRISQPTITEPEAAGMDPADLLA
CGQVIAGFLLQKRAKAAASLIA

5121752..5121871 PHAGE_Pseudo_Dobby_NC_048109: GpE familygtail protein; PP_04798; phage 4.92e-21 - NONE NONE NONE
MADLAVTFHWAPDHMDRLSLTELMEWRERARVRSSADGE

5121861..5124620 PHAGE_Pseudo_Dobby_NC_048109: tail tape measure protein; PP_04799; phage 0.0
- NONE GO:0005198 - structural molecule activity [Evidence IEA]; GO:0016032 - viral process
[Evidence IEA];
MANDLQLRVLLSAIDRATAPLRRIMQGSDATARALKATRERLKQLNAQQSDVRAFRTQRGALEQVSTALAAQQTRVKALAQQMA
AAGNPTRALTRDYNRAIREAGFLKQQHLQQSQALQQLRTRLSNAGISTRNLGQHERDLRAQIQAANGAINSQAQRLRNLSQQQE
RLTHARNTYSRGIQSAAALAGTGMAARATGMYTGDKLRQMLGVGYEFDATMSATQAVTRIERKDDPQMQALRQQARTLPLSSK
FTDKEVAEGQYFLGRTGYNAKQILGAMPGMLNLAAAGDMDLGASADIASNIQTAMGIPAEKMDQVADVLTAAFTRNNVDIRMLG
DSLKYSAGVGREYGQSLETVTAATALLGNAGVQGSQAGTSMRSVLTRLGLSKAVAQLGVKTQDANGNMRDMLDILKDINDKTKK
MGNIQRGAIYKDIAGQYAVTAFGTLMRAVESGQFQSMRGSLDNSEGEAARVASTQLDNLKGDMTMLHAALENISVELFDKNSPW
LRELAADLSHLLHNVGEFLKANPQVSKGIVITVAAFSALMATVGSLAITLAGILGPMIAVRFMLSTIGIRLPGLIGLLKLLFAPIRMLAG
LLIGPLVTALRVVSIALWGLAANPVVLAIAAVVAVLAGAAYLIYRNWDAVKAYLLGLWEEIKAGFDGGIGGILSTLMNFSPLGLIYRA
FSGVLGYLGIDLPARFTDFGNMIVQGLVNGLLAGIGGQIKRAVQRVGGAAIDWFKDKLGIHSPSRVFADLGGFTMAGLAQGLDAGQ
AGPLGVIARIGQGLVNAGRQAVAGLDSDLTRSARPTITPPAVVTELVAAQRQRSPTFDQPLLAMLGDLGKSAGAIGALVLGASAP
AQAITIDNRPPVSSAQAAVSIGGDTYYITIQAGPGSDAADLKRTLSQLLDERERNKAARLRARLQDRE

5124626..5125066 PHAGE_Pseudo_Dobby_NC_048109: tail protein; PP_04800; phage 2.74e-100
- NONE NONE NONE
MMLSLGMFVFSLHTLAYQEFQRQTEWRHASSSRIGAQPARQFVGRGDDTITLPGVLLPELAGSALSLDVLRQMADTGSAWPMV
EGTGRIYGLWVIERVTETRTLFFADGTPRRIEFSLELKRIDDGRTDLLGSVLGTAGNLLRRIL

5125063..5126337 PHAGE_Pseudo_phiCTX_NC_003278: tail protein; PP_04801; phage 0.0
- NONE NONE NONE
MIDAALARVTGYLTSAVDQLQRDAGYPVPVFRLTVDGNDIAQLISPRLIALDLTDNRGIEADQLSVTLSDHDGLLAIPPRGAVLHLW
LGWSDSGLVDKGTYTVDETEHSGAPDVLSIRARSADLRKGLKVKRERSWSSPKTLGDVLTDIALGNNLKPVLAPALAGLPILQLD
QANESDANLLTRLGEDFDAVATVKAGCLLCLPAGGGKTASGLALPHITLTRQDGDQHRYLQADRDSYDGVRAYFYDVNSAKKQ
EAIAGAKGDNLKDLRHTYSDRQSALRAARAEWNRLQRGSATLSYVLAKGRPDLIPELTYTLQGVKTEIDAIIWYGGNVQHSLSAD
GGYITSLELESKLPEDLVSDLADDTGGDYTGIIAYYRDGKSGTEKNVTAGDQSKPRRLRYLYSTKASAKRAVDREWGRMQKINR

complement(5126383..5126952) PHAGE_Shigel_SfII_NC_021857: hypothetical protein; PP_04802; phage
2.49e-27 - NONE NONE NONE
MLVEQLLRPFGYLAIRHPHKWRVDWLYPGTLALVTTGLILLGSPKKIIASDGGGLVQLVLSFVQGLPGFYIAALAAIATFGRPDIDEV
LPAPTPKVVIRSRGVDNLVDLTRRRFLAMLFSFLTAESLLIIVFSLFLISHGAGLFSFYDFRFRSEAFVAGGICFFYFLLFFQMIVSTF
WGLYYLGYKLHE

complement(5126957..5127958) hypothetical protein; PP_04803 N/A -
NONE NONE NONE
MEVHRTATFYDLKLTAKGYSRSEEYAADFEAAPKTLLELYSFIKQVFDGGDQIIQKGRTEKSVKHYLADMELRDTKLVLLVNRSDP
TAPDAVSTDPENKSRVVHKKPPNHGGDFSAHVVIELKPVKGDNYYLCVIETVYGSGLHASAVAGYIRHVIRYCKKQFPEEFKIAHI
GGVKDKSGAQVMVSHLHYVELQGHPSEEFEKDLEGGTLGDIELLDFSEKGAEWDEQGEILENSRIVKLSPQKKIIGDLKKVIGQV
RNRALKKDDASYFQMRIRFKNEKEEPREATISTDTGYLIDEKKYVKKHVIRAEIVNTASLEVISNVIVKEVISLME

complement(5128324..5128755) PHAGE_Faecal_FP_Lugh_NC_047912: LexA family transcriptional regulator; PP_04804;
phage 4.12e-07 - NONE NONE NONE
MSTDKNAQTETSVRLRAALEAKGLSIKEAAEACEIPYRSFQNYTLGLREPNAEALGTISSRLGISVDWLLTGDGQMLRGASVKVA
HDGAENPREQALLALWRELDEGEQREIQLAAEEKKRLKILEQRLAELEAVVADAKRLA

5128821..5129051 phage-associated protein, BcepMu gp16 family; PP_04805 N/A -
NONE NONE NONE
MTPNQIRARLVEKGSSYRKFALARGYEPRNVTQVVARWAGAERLPNGRLAYAILKDLSEEIGADVVPGIRQPTTEQ

5129081..5129584 PHAGE_Pseudo_phiCTX_NC_003278: hypothetical protein; PP_04806; phage 7.71e-
106 - NONE NONE NONE
MKRPLLETRRQVVSAIIGAYPGGRECAAARLGLDLKKFDNHAYENAGSKPLSDDQLRLLEQETGTSHFPEYVAHLYGGMFVQMP
EPTQLDNLDLYARGVATAIRRGEVDRIIAEALRDGEIDEAELAEIIVAHRKHLAARHAEVGAVITLHRKAPHGQRVSGSGEHE

5129581..5129859 PHAGE_Pseudo_phiCTX_NC_003278: hypothetical protein; PP_04807; phage 1.19e-
20 - NONE NONE NONE
MSTYKLVCPHCASRMRIRTSDGKHIFLRVAYLQCTNEACGWSVRAEFEMTHEMSPSGMPNPSVYLPPAPTALRREAMRQQGD
NQLDLLEATA

5129856..5130215 PHAGE_Pseudo_Dobby_NC_048109: hypothetical protein; PP_04808; phage 5.87e-
10 - NONE NONE NONE
MNVTPFTQQQADDYRTFMQQAARRFIEKHAAEHLDDDGLFERTVSYLVNSLDVPAFMAGRLALLAMSERLPKGLAWVAFDMAA
GPDQSVRMVLDRRTGQMTPLPSRFLPHRFLAAPATH

5130283..5130516 PHAGE_Pseudo_phiCTX_NC_003278: hypothetical protein; PP_04809; phage 1.60e-
12 - NONE NONE NONE
MPDAVSIQFDMPKPVAEALLTSLRTELRRGLVQHWYDDRYRTVPEGLRSSRILDDYPALAGQKRTIGALRAALAANL

5130535..5133252 PHAGE_Pseudo_Dobby_NC_048109: toprim domain-containing protein; PP_04810; phage
0.0 - NONE GO:0003896 - DNA primase activity [Evidence IEA]; NONE
MHEKLRAKVLPKLEADYGLKHMKGTNFMRKGKCPAHRCGQRTLYTFFDSPWMLICGRPEACDHRVHIKDVYPELFNDWSDQAP
ATPDNPTATARAYLEFARGFRLELIDGWFTQENYWDNRLKIGSATVRFALEKGGYWERLIDRPERFGKQKARFKQGESYKGVW
WCPPTLDPTEVDELWIVEGIFDAIALMHNGRAAVSMMSSAPFPAESLKALKKRCQDEDKRMPRLIWALDNEPVAKANARRWAKE

ARELGFKCDAAVIPQRGAKKIDWNDLHQRWAFIEGDDARTKRVEMDLEEARYEGSLLLAETAEEKGLLMYSWNERKEFHFSFRS
RLYWFKLDIEAYDRAIKELESSENAEDQLLNDKQRRERALRRAGSVVRIGNCYFQALYFMRNEQTDEAWYYFRVERPDAPTVKA
TFTSAQISASAEFRKRLLNVCNGAMFTGTPQQLERMLEPQLDRLKSVHTIDWIGYTREHGVYVFNDLAIAGGKVHKLNEEDFFDID
RLSIKSQSQSPVLHINPDLAAYNEGWFEMFWKCFGVRGVVVLAWWLGALYAEQIRQIHKSYLFLELIGEAGSGKTTLVELCWKLT
GRTEYEGFDPSKATPASRARNFAQVGNLPVVLIESEREQKEGAPVKHFDWDELKTAYNGRSVRSTGVKNNGNDTREPPFRGAL
LIAQNNAVNASEPILQRLGHVNLTREHQTPETKLLAELLERMPVEQLSGFLVKALQPEAKIMALLDERTSGYEQELLAIPGIRTVRIA
KNHAQLRSLVDCLQLVVPLDGERAQLVHAEVSRMALERQQAINADHPVVREFWDLFEFLNGPLNELPGNLNHSRKKEFVAVNLN
EFIEVAANKRQQVPNLGELKRLLKTSKSPKFIESNKAINSGRQVDAFDKPKTVRCWLFQLV

5133306..5133446 hypothetical protein; PP_04811 N/A -
NONE NONE NONE
MQNDETPLYEHSRGEWIATLTLTGLTLVVLVIAGYYTPAVLAAVIH

5133499..5134230 PHAGE_Pseudo_KPP25_NC_024123: hypothetical protein; PP_04812; phage 1.03e-38 - NONE NONE NONE
MQKHFTITQAMREEVADRLTLQAVAQHGPRIAADLQALNDQFWSKHNAAVEALPGLDKKHWADLIVAGALASVIKCDVYYMKPR
DGMEPTRCGLHLIYHEYKGDRRNALVQRIIDSPAYAGVKPFVERNAHHDRHWSLRLTNPNGSVPRLRGMDVLTDPAHETLALLC
SEDLQGVMKGAEAFRAQALDVLLACRTSRQVEDLFPEAAKLLPQPVKTERALAPTELAANVRGMLNAGVPPVVAA

5134325..5134582 PHAGE_Pseudo_phiCTX_NC_003278: hypothetical protein; PP_04813; phage 1.71e-29 - NONE NONE NONE
MEALQRLLPVAQRDTGQSRIVGRFLLSLYNGNAFPFCLTDLRGLDTQLWEDCLALLRLDRRPEVEIHQYVQDGEHIWSALKHAW
A

5134652..5135092 hypothetical protein; PP_04814 N/A -
NONE NONE NONE
MQVQTPEVGAEKASTPRYDTIVIRGATGKDVPREVDGGEVVSWARGHELAAGDALLEFVNYVADGDCGITPELSTKARKALDLM
ERRSRLGWEADEQPEDWPASVNRAAQTAREVFNGSHEDAIQAIEYMHALLLQAAPVVQGGDV

5135089..5135265 hypothetical protein; PP_04815 N/A -
NONE NONE NONE
MKPCTLGKRHSWTFVRNVVTSHLSGRFGRITKRGFYRCECGAEKYGNAGDLSGGSTHA

5135258..5137168 PHAGE_Pseudo_Dobby_NC_048109: DNA cytosine methyltransferase; PP_04816; phage 0.0 - NONE GO:0008168 - methyltransferase activity [Evidence IEA]; NONE
MLKRTLYHFHFCCGLGGGAKGFNRARPRVGNVEAQWECLGGIDVDPAGLRDFERLAGVPGTLLDLFTRDQYVRFHGKEPPAG
WREATPEDIRRAAQGKRPDAVFISSPCKGASGLLSEKLSLTPKYQALNELTLRCIWLMGEAWADDPVPLIVFENVPRLASRGRHL
LDQINSLLGGFGYAVAETTHDCGELGGLAQSRKRFLLVARHVEKVPPFLYEPEKKSLRAVGDILGRMPLPGDIEAAGPMHRVPSL
QWKTWVRLALVRAGSDWRSLNDLAVEDGYLRDLIIVPEYHRGVLGVNHWGDSCGVVAGASRPMNGRFSVADPRAPANALQYQ
QYGVRRWTDTSGAIIGVKSPGQGTYSVADPRGQSFGKYPVTDWDGPSGTVIAASTTGQGAFAVADPRPGGVRHNNVFRVVSM
GSHAGTVTGGHSPSSGGQAVADPRYHNWHPGASSRKLHVGEWGSATGTVTGSQQVASGALSIADPRVLDRTKGDAYLTGGH
YGVVGFDQSAGAVSASARHDNGRWSVADPRMPAANDRLTCIIQSLDGTWHRPFTTLELAALQSLVDPEEQLVLDGLSDSDWRE
RIGNAVPPAAAEAIAGVMGTTLLLAEQGETFMLSNTPIWVRPVAVALSVAQQEVQQ

5137165..5138397 PHAGE_Pseudo_Dobby_NC_048109: phosphoadenosine phosphosulfate reductase family protein; PP_04817; phage 0.0 - NONE GO:0003824 - catalytic activity [Evidence IEA]; NONE
MKALSPRQSDIFAAGAQRLQMTESIELTIQSMQAYGADHEHWAVAWSGGKDSTTTLTLLIWLIDTGRVKAPKTLTVFYADTRQEL
PPLAIAAHQIMDELRDRGIHVEVVCAPLDKRFMVYILGRGVPPPNNNTLRWCTRQIKIDPMQSALEQRLAALDGNVLMITGVRQG
ESSIRDKRIEMSCGTDGAECGQGWYQKVLPEAKGLKGRLATLAPLLHWRVCHVWEWLKHWAPLAEFGDWSTAMIADAYGGDE
AEEINARTGCTGCPLASEEKALETVLTMPHWAYLAPLRGLKELWRELREPQHRLRKAGIERLKDGSIAANPQRMGPILLESRLMG
LERVLAIQAECNAAADRLGRPRIDLINAEEESRIRELIAAGTWPDGWDGDEPIATTPLDKIFADGAVQPLLFC

5138408..5138584 PHAGE_Pseudo_Dobby_NC_048109: hypothetical protein; PP_04818; phage 9.69e-21 - NONE NONE NONE
MHDLLKMLDNPRSLLNFSLAVLVVLAVFFMLKSGAQAASQPASSLSTSTEAQSRGKQP

5138581..5138949 PHAGE_Dickey_vB_DsoM_JA11_NC_048077: putative ASCH domain-containing protein; PP_04819; phage 3.17e-39 - NONE NONE NONE
MKALSIRQPWAWLVANGHKDIENRDWATNFRGRFLIHAAKSMTRDEYEEARDFAAYNGVTIPAPHELERGGIVGEASIIGCVDRC
NSLWFFGRYGFELADAKPLPFQPMKGQLGFFEVEVAQ

5139142..5139345 PHAGE_Pseudo_C5a_NC_047790: transcriptional regulator; PP_04820; phage 1.38e-05 - NONE NONE NONE
MAELDRFMREAEVLEATSLARSTLWREVKAKRFPSPVQITPGRVGWRQSDINRWLENPMGWTPTRAA

complement(5139352..5140554) PHAGE_Pseudo_PPpW_3_NC_023006: putative integrase family protein; PP_04821; phage 5.02e-40 - NONE NONE NONE
MLTERQIRALKPAEKEYTVSDGRSARGEGVLMLRVRPNGTKEFYFQRRKNGRKLKTKLGTWPTMALTEARDRSREEKEIQVEA
GTFKELMAAYVAKLKQEGAASAEHVEWSFKHYVSEPFPTLVERPAVLIGPADIRDILAKMIAGGVTTMTNRVRSRLHSAFQSALQ
QDYNPRTYLEQENRFGLTSNPVASIPVQEDWEQPGDRALTEKELQALWHLLPEKLSLTTSELLKFLIASGGQRPEQLLRSDRTMY
QRDHVMIRNGKGGEGERAMHVVPYNKLMRASLKEMDCISEKSAYPFQGKEEGKSLNPQSLSRAVTKLYGRHHKSFNGPFTLRD
IRRTCKTLMAKAGLTKELRDRIQGHAFNDVSSKHYDRYDYFQEKKRGLDRWAAWLEKNVIDTKK

5140569..5140582 attR N/A
TTGGGGGCCGTTTC

complement(5140889..5141236) type II toxin-antitoxin system RelE/ParE family toxin; PP_04822 N/A
- NONE GO:0008657 - DNA topoisomerase type II (double strand cut, ATP-hydrolyzing) inhibitor activity [Evidence IEA]; GO:0042803 - protein homodimerization activity [Evidence IEA]; GO:0030541 - plasmid partitioning [Evidence IEA];
MSPVVIRFTDTAEQSIEDQVHHLAPFQGEQAAFQSVLSLLDEIEEKISLAPKGYPVSQQASLLGVLSYRELNTGPYRVFYEFHEEQ
GEAAVILVLRQKQSVEQQLIRYCLVGPIE

complement(5141246..5141497) type II toxin-antitoxin system Phd/YefM family antitoxin; PP_04823 N/A
- NONE NONE NONE
MRVETISYLKRHAADLDLSEPMVVTQNGVPAYVVESYAERKQRDEAIALVKLLAIGSRQYAEGKHRSVDDLKARLSRRFAQPE

complement(5141711..5142694) PROPHAGE_Pseudo_PAO1: bacteriophage integrase; PP_04824; phage
0.0 - NONE GO:0003677 - DNA binding [Evidence IEA]; GO:0015074 - DNA integration [Evidence IEA];
MSITKLPDGRWFVDVEPIKGKRFRKRFKTKMEAQQFEATARQKCAENPCWTLRPKDRRRLSELVELWYELHGQTLSNGHRCVA
ILRLVAKDLGDPVAVSLEPAKVARLRSRQIANGMSGKTANNRLGYLKSMYNELCQLGVIDYENPVGRMRPLKLQERPLSYLTKHQ
VSELLTALDARTTSPHPKMVARICLATGARWGEAQALTPERLKGNAVIFANTKSKRVRSVPISEELGADLRLHWQTHGPFTNCLG
VFRLVLLSTSIKLPKGQASHVLRHTFASHFIMNGGHIVTLQHILGHASLSMTMRYAHLSQDHLSEAVRFNPLIG

complement(5142694..5143986) PHAGE_Pseudo_Pf1_NC_001331: hypothetical protein; PP_04825; phage 0.0
- NONE NONE NONE
MKKISHQIRVSIESDGQVLESPKGRLFFDDTTAQFTDLSGVRILRCGVDTVRQLYNGKLRPEVMALFDLSVDVVEFAGYEWSKGR
IGRDSGYQYRLQNAEMGLILLIKNHNIKVDTIGSHLKIEVSPHALDGADPRILQGVLDDLAAVLSHCETNQAAVHIALDVQGWKPP
RDLVDRMHCRSRRVRQISGIERIEFDGNASVYGRGETYMFGSANGLQLSIYNKTLQARATDKLDYWESVWATLNGDPFGDGDP
AYNPLETVWRLEFRFHHSIVQQFSEGSRMASGEVIGCRTYEGLCPHLQGLWNYACESFKLLSRTAVYDPFWSLISQDARVQVEC
DPLIERTEYRRYYKTAKGFSGRNCEMFLGQFVSLIARERVPAKKAIESARKLEFWHVIEDHYLAKGWTRRDLERHIHKLMCDRYL
RRGYAV

complement(5144245..5145506) PHAGE_Pseudo_Pf1_NC_001331: putative assembly protein; PP_04826; phage
1.73e-08 - NONE NONE NONE
MAIKIHHGPNGSYKTSGAIQDDLIPAIKKAASSSPTCAAPANGSSKCRRRPPAATSSTSTSRTWMTWKRCAPGSCGRRVARSSS
STKPSSFSPGAKPTSSASTSRTARKRPRQPGGPWAGWMPGPGTGISTGTSSSPRRTSPISATTSARRKRPICTPTSPSSAFGAA
TRKASTRRRTTNRRPATSSRSRKSARRPSPSMNRQPPAPSPTPSPARAFLDNLRFFYSWQFRPLLLGLWFMTADLVCSWATLY
RRLLLELLRLLKPVLLWVLRVLLVRLILMLLMMYLGTQAFRALLLGIPSPAATSSSRRPCCPPPGAAPICSPSGARTAANSLSPIATP
TPAMPWCRGATALRNASRAVGPAMPPAPGVVPWAMRRRLRPPRRACRPPPRTAPPCGRWFLIPAACRARSTGA

complement(5145508..5145858) PHAGE_Pseudo_Pf1_NC_001331: hypothetical protein; PP_04827; phage
2.05e-17 - NONE NONE NONE
MDIPFLSDILAWMQSLWDFLYSGVYDFVTDAFVLLTKMAIKGWFEMQLFVAEIGYKAFREVVGGIGIGSTITSYYSSLDGDLRSLLA
FFGLPDAVNMIFAAIGTRFSMSFIPFIGK

complement(5145868..5146998) PROPHAGE_Pseudo_PAO1: coat protein A of bacteriophage Pf1; PP_04828; phage
8.62e-10 - NONE NONE NONE
MIITLLFGSAAHAEYYYWYMGYFNKKVSSPTAGCDLYFSSFSKDPGRVFVMEPSSNPTEAGKVFYCVVRSGDWILFNTDVYLKG
DRCPEGTELDLSAGECRENKCKILAGSLYEKGGHQAPISRFINYLGCEIAVSSIDGCIGPAEGEAGGTFCRVIGSFTGNWFTSKGS
CAFGCDVGPGDGPPPGGDGGTGGDGGSNPPGGDGGSDGGTKPGNGGGDDGSSGGGGGGGGGGNNPCQGHVGSDCGTT
PGGDGSSGGDGDGSGSSGGTDGGDGSGGGGLKEPKQGSFDKTIKEYDDAIAKAQKDFQELQGKFESVLASKFDIHLGTGGGS
LPCWDFTALGQRYDVCLTQYAQELSVIRYVVLFIAAILAGWIVFYRS

complement(5147126..5147344) hypothetical protein; PP_04829 N/A -
NONE NONE NONE
MEKMKTLFRNASIATAGLAVANVSFAESLLDETTKGVLAQASTDGGSVAKLVIAAVAVLVGLALVIGAMRKA

complement(5147358..5147609) PHAGE_Pseudo_Pf1_NC_001331: hypothetical protein; PP_04830; phage
8.52e-21 - NONE NONE NONE
MEGSVSVQVCKTWVQNADGTVGCTHLEWIQTYLLPPEAEGYLTLLMGGFDPSAFRLGFAGTIGLFAVGLGAGLIISAMRKARN

complement(5147730..5148164) PHAGE_Pseudo_Pf1_NC_001331: ssDNA binding protein; PP_04831; phage
5.85e-101 - NONE NONE NONE
MNMFATQGGVVELWVTKTDTYTSTKTGEIYASVQSIAPIPEGARGNAKGFEISEYNIEPTLLDAIVFEGQPVLCKFASVVRPTQDR
FGRITNTQVLVDLLAVGGKPMAPTAQAPARPQAQVQAPRPAQQPQGQDKQDKSPDAKA

complement(5148680..5148966) PHAGE_Pseudo_Pf1_NC_001331: hypothetical protein; PP_04832; phage
4.28e-49 - NONE NONE NONE
MSSPNYLRQTHAPDCGCSVCWSARQVIPLHSPSPCPDCRPPGLPYLEDGRWLCRPRSFCAKHDPSRRPPKYWHVVYDSGKP
RPLCPCAKHSNWRAGA

5152806..5152817 attR N/A
CGCCCGCTCCAG

#### region 6 ####

complement(5787383..5788246) PROPHAGE_Salmon_LT2: integrase; PP_05419; phage 1.06e-06
- NONE NONE NONE
MDDLTYTLRQLCQRNRDGSHNTQADRMRSLALAARQLREAGFRQMKASSLKGKHVQALLDRWQGEGLSSGTLKNRLSHLRW
WAEKIGKSGILPADNTQLGVSERRYVTNVSKARELGSGLDLVTDAHVRMSLKLQAVFGLRREEAIKFQPSYADRGDHLALKGSW
TKGGRERTVPITTPEQREVLQAAHHLAGAGSLIPAHKTYIQQRHVYDGQCKTAGLSHMHGLRHQYAQSRYETLTGWPAPAAGG
PSVKGLSPAQRVEDSRARQTISRELGHERVQITAIYLGS

5789613..5789689 tRNA-Met; N/A
GGCTACATAGCTCAGTCGGTTAGAGCGCAGCATTCATAATGCTGATGTCCCAGGTTCAAGTCCCGGTGTAGCCACCA

5789829..5790239 hypothetical protein; PP_05420 N/A -
NONE NONE NONE
MFSELGFSNFDVLLPIACTIGAIFGSLAQTIIANYNLNSLPRKEGEMKSASPQLQEMRSAWLFLRLFVGGVLGFVVGLYFVGALQE
TPAVFAKIWALSFVVGYAAPKIWVVQERNLLNRIDSSLDQPEKASTGVPD

complement(5790220..5791221) PHAGE_Erwini_ENT90_NC_019932: phage integrase family protein; PP_05421; phage
9.12e-93 - NONE GO:0003677 - DNA binding [Evidence IEA]; GO:0015074 - DNA
integration [Evidence IEA];
MTVRKDGKTWTADFYENGRSGRRIRKKGFATKSAAIRYEQDFFAVKGETGRPLDDRLSDLVKVWYDLHGCTLKDGKQRLARCE
ALAKRLGNPLAFEFDSLAWARYRQRRLTEVKPETVNHEQRYLSAVFSELIRLGSWHKENPLGKVRQIKTDQVELTFLSLDQVARL
LEECKASTNNHTYPVALLCLATGARWEEAESLTRGAVHGGKVHYHRIKNRQSRSVPIPDELERLIFKVGMPGSGRLFMSCRAAF
RCAYQRCGFQTPGQMTHILRHTFASHYMMGGGDILTLQRILGHSSITMTMRYAHLSPEHLASAMSLSPLYQIKHFASQVHQ

complement(5791218..5792510) PHAGE_Pseudo_Pf1_NC_001331: hypothetical protein; PP_05422; phage 0.0
- NONE NONE NONE
MKTPIHPTRLVLEENGDFHKSPKGMLFMDPLNGQFTDLSGVRILRCGVDTVRQLYNGKLRPEVMALFDLSVDVVEFAGYEWSKG
RIGRDSGYQYRLQNAEMGLILLIKNHNIKVDTIGSHLKIEVSPHAIDGADPRILQGVLDDLAAAVLSHCETNQAAVHIALDVQGWTP
PADLVDRMHCRSRRVRQISGIERIEFDSNASVYGRGETYMFGSANGLQLSIYNKTLQARATDKLDYWESVWATLNGDPFGDGD
PAYNPLETVWRIEFRYHHSIVQQFSEGSRMASGEVIGCRTYEGLCPHLQGLWNYACEAFRVLSREGMYDAFWSLISQDARVQV
ECDPLIERTEYRRYYKTAKGFSGRNCEMFLGQFVSLIARERVPAKKAIESARKLEFWHVIEDHYLAKGWTRRDLERHIHKLMCDR
YLRKGYAI

complement(5792769..5794030) PHAGE_Pseudo_Pf1_NC_001331: putative assembly protein; PP_05423; phage
1.58e-08 - NONE NONE NONE
MAIKIHHGPNGSYKTSGAIQDDLIPAIKKAASSSPTCAAPANGSSKCRRRPPAATSSTSTSRTWMTWKRCAPGSCGRRVARSSS
STKPSSFSPGAKPTSSASTSRTARKRPRQPGGPWAGWMPGPGTGISTGTSSSPRRTSPISATTSARRKRPICTPTSPSSAFGAA
TRKASTRRRTTNRRPATSSRSRKSARRPSPSMNRQPPAPSPTPSPARAFLDNLRFFYSWQFRPLLLGLWFMTADLVCSWATLY
RRLLLELLRLLKPVLLWVLRVLLVRLVLMLLMMYLGTQAFRVLLLAIPSPAATSSLRQPCCPPPGAAPICLPSGARTAANSLSPIATP
TPAMPWCRGATALRNASRAVGPAMPPAPGVVLWAMRRRLRPPRRACRPPPRTAPPCGRWFLTPAACRARSTGA

complement(5794032..5794382) PHAGE_Pseudo_Pf1_NC_001331: hypothetical protein; PP_05424; phage
2.05e-17 - NONE NONE NONE
MDIPFLSDILAWMQSLWDFLYSGVYDFVTDAFVLLTKMAIKGWFEMQLFVAEIGYKAFREVVGGIGIGSTITSYYSSLDGDLRSLLA
FFGLPDAVNMIFAAIGTRFSMSFIPFIGK

complement(5794392..5795522) PROPHAGE_Pseudo_PAO1: coat protein A of bacteriophage Pf1; PP_05425; phage
8.77e-10 - NONE NONE NONE
MIITLLFGSAAHAEYYYWYMGYFNKKVSSPTAGCDLYFSSFSKDPGRVFVMEPSSNPSEAGKVFYCVVRSGDWILFNTDVYLKG
DRCPEGTELDLSAGECRENKCKILAGSLYEKGGHQAPISRFINYLGCEIAVSSIDGCIGPAEGEAGGTFCRVIGSFTGNWFTSKGS
CAFGCDVGPGDGPPPGGDGGTGGDGGSNPPGGDGGSDGGTKPGNGGGDDGSSGGGGGGGGGGNNPCQGHVGSDCGTT
PGGDGSSGGDGDGSGSSGGTDGGDGSGGGGLKEPKQGSFDKTIKEYDDAIAKAQKDFQELQGKFESVLASKFDIHLGTGGGS
LPCWDFTALGQRYDVCLTQYAQELSVIRYVVLFIAAILAGWIVFYRS

complement(5795650..5795868) hypothetical protein; PP_05426 N/A -
NONE NONE NONE
MEKMKTLFRNASIATVGLAVANVSFAESLLDETTKEVLTQAGTDGSSVAKLVIAAVAVLVGLALVIGAMRKA

complement(5795882..5796133) PHAGE_Pseudo_Pf1_NC_001331: hypothetical protein; PP_05427; phage
8.52e-21 - NONE NONE NONE
MEGSVSVQVCKTWVQNADGTVGCTHLEWIQTYLLPPEAEGYLTLLMGGFDPSAFRLGFAGTIGLFAVGLGAGLIISAMRKARN

complement(5796254..5796688) PHAGE_Pseudo_Pf1_NC_001331: ssDNA binding protein; PP_05428; phage
2.39e-100 - NONE NONE NONE
MNMFATQGGVVELWVTKTDTYTSTKTGEIYASVQSIAPIPEGARGNAKGFEISEYNIEPTLLDAIVFEGQPVLCKFASVVRPTQDR
FGRITNTQVLVDLLAVGGKPMAPTAQAPARPQAQAQAPRPAPQPQGQDKQDKSPDAKA

complement(5797204..5797494) PHAGE_Pseudo_Pf1_NC_001331: hypothetical protein; PP_05429; phage
2.95e-63 - NONE NONE NONE
MAASPYYLRQTHAPDCACSVCWSARQAIPLHSPSPCPDCRPPGLPYLEGGRWLCRPRSFCAKHDPSRRPPKYWHVVYDSGKP
TPFVPVREAFQLEG

complement(5797706..5797978)	PHAGE_Pseudo_Pf1_NC_001331: hypothetical protein; PP_05430; phage
1.88e-60	-	NONE	NONE	NONE
MEESGIVGFTVTGAVEKVTDFRTAPFCSQAVFAQMLGLEDITEDVVRGWVETKTIPTAKIGRRRVVNLHRIRRDLDRGKSIFCQG
DYDGD

5798088..5798354	hypothetical protein; PP_05431	N/A	-
NONE	NONE	NONE
MIKDRLITLFNKERTSVWFEKQTGIDRYRWGNVRNGKARITDAEIEAVIQIFPQYALWLVTGNIAPESGQTSPDYDEANRNLASPN
AG

5798486..5799322	PHAGE_Methan_psiM100_NC_002628: hypothetical protein; PP_05432; phage	4.91e-
18	-	NONE	NONE	NONE
MSTVGSVRARMEDEMPKTAQVIAAVYDEDHDAGRVVGRSLKSLREAVGLTQLQMARKLGVGQAAISKIEARGDVQISSLKKYVD
ALGASLRIEAAFKADSEISTRLREELALEEHSDRQLVLPIFSSDEIFLEESKDLILSIHPQYSDKILAGKKTVELRRRFPLTTAKGTKVY
IYSTSPVRAIVGSAEIAGIIKLPIKDMWKKYSKCAFIKKQAFESYFEGLSEGFALELKNAQAFDKPIELLELRERFNFTPPQSFIYAKQ
EMRRALMDEQTSLSN

5799297..5800835	PHAGE_Methan_psiM100_NC_002628: hypothetical protein; PP_05433; phage	6.06e-
47	-	NONE	GO:0008080 - N-acetyltransferase activity [Evidence IEA];	NONE
MSRQAYLIDTNVIIGLEDNKAVQPAFAAFMKLATKHKVDFYIHEAARDDIARDKDVQRREISLSKLEKFSCINKVRGFNAHVLGDEF
GQIRKPNDIVDVTLLHALHIGTADFLVTQDRGLHERARRHSSELGRRVLHVADAVELLKTTFEPIESPVRFVEEVAAHTIPLSDSIFA
SLREDYPPFDQWWKDKCVREHRTCWIISSDQNQNIAGLVVRKDESPENTDATLPAKKILKICTFKVRPESRGFKLGELLLKKVFW
FAQKNSYDLVYVTTYDGQAALIDLLEYYGFQHTATKPDGELIYEKTFSQDVLVRNSGEDLFKTARLNYPRFVTDQDVRAFGIPIKE
GYHDILYPDLKSDYQGDLFENAGLVGPQRPGNTIRKVYLCRAKSNLGEPGSLLFFYKGKSKHDPSQAFTTIGILEEVATASSTKEL
MKLAGGRSVYSEKQLEDWAASSHNPIKVINYLLACYIDPPVGMIELQEMNVFTGHPPQSIFEIKVNIDRLLARSGVGFKV

complement(5800847..5801374)	ATP-binding protein; PP_05434	N/A	-
NONE	NONE	NONE
MTVFVAGVHGVGKSYLCEKYAQAHDVLHESASSLIRKEKNQLNWSTDKKVVNADDNQVALVAAVRRISEQGNSLLLDGHFVLIG
PHAEFIRLPPSVFKDLNLTGVILIEASPEIISSRLSSRDDSKSTVDIALFLEAERAHAKLICETLNLPLEILFQPTHEVFADVAKKIIGKI

complement(5801751..5802056)	PHAGE_Gordon_Daredevil_NC_048021: antitoxin; PP_05435; phage
4.35e-20	-	NONE	GO:0003677 - DNA binding [Evidence IEA];	NONE
MATNGMRPIHPGEILRDEFLMELDISPAALARALKVSAPTVNDIVREQRGISADMAIRLGRYFDTSAQFWMNLQSEYSLATAYAAN
GKQIEHEIEPLLAHG

complement(5802068..5802346)	PHAGE_Gordon_Daredevil_NC_048021: toxin; PP_05436; phage	5.55e-
27	-	NONE	NONE	NONE
MILTFRCDETRQLFETGLSRRWGAILTVATRKLAMLHAARELRDLRSPPGNRLEPLQGKRAGQHSIRINDQWRVCFVWTDAGPE
EVEIVDYH

complement(5802399..5802527)	PHAGE_Klebsi_4LV2017_NC_047818: tyrosine-type recombinase/integrase; PP_05437;
phage	1.38e-08	-	NONE	NONE	NONE
VALQRILGHSSMMMRYAHLSPEHLESAMRFSPITQSGYKLTT

Supplementary Information 2: EMBOSS STRETCHER output of the global pairwise alignment between the tail-collar fiber protein from the pyocin-encoding gene cluster of *P. aeruginosa* ATCC 27853 and tail fiber protein from the pyocin-encoding gene cluster “S14”.

```
########################################

# Program: stretcher

# Rundate: Thu 27 Nov 2025 09:58:04

# Commandline: stretcher

#    -auto

#    -stdout

#    -asequence emboss_stretcher-I20251127-095801-0073-54089967-p1m.asequence

#    -bsequence emboss_stretcher-I20251127-095801-0073-54089967-p1m.bsequence

#    -datafile EBLOSUM62

#    -gapopen 12

#    -gapextend 2

#    -aformat3 pair

#    -sprotein1

#    -sprotein2

# Align_format: pair

# Report_file: stdout

########################################


#=======================================

#

# Aligned_sequences: 2

# 1: ATCC27853

# 2: S14

# Matrix: EBLOSUM62

# Gap_penalty: 12

# Extend_penalty: 2

#

# Length: 701

# Identity:     701/701 (100.0%)

# Similarity:   701/701 (100.0%)

# Gaps:           0/701 ( 0.0%)

# Score: 3654

#

#

#=======================================
```

```
ATCC27853  1 MTTNTPKYGGLLTDIGAAALAAASAAGKKWQPTHMLIGDAGGAPGDTPDP    50
             | | | | | | | | | | | | | | | | | | | | | | | | | | | | | | | | | | | | | | | | | | | | | | | | | |
S14          1 MTTNTPKYGGLLTDIGAAALAAASAAGKKWQPTHMLIGDAGGAPGDTPDP    50

ATCC27853  51 LPSAAQKSLINQRHRAQLNRLFVSDKNANTLVAEVVLPVEVGGFWIREIG   100
             | | | | | | | | | | | | | | | | | | | | | | | | | | | | | | | | | | | | | | | | | | | | | | | | | |
S14          51 LPSAAQKSLINQRHRAQLNRLFVSDKNANTLVAEVVLPVEVGGFWIREIG   100

ATCC27853 101 LQDADGKFVAVSNCPPSYKAAMESGSARTQTIRVNIALSGLENVQLLIDN    150
             | | | | | | | | | | | | | | | | | | | | | | | | | | | | | | | | | | | | | | | | | | | | | | | | | |
S14          101 LQDADGKFVAVSNCPPSYKAAMESGSARTQTIRVNIALSGLENVQLLIDN    150

ATCC27853 151 GIIYATQDWVKEKVAADFKGRKILAGNGLVGGGDLSADRSIGLAPSGVTA    200
             | | | | | | | | | | | | | | | | | | | | | | | | | | | | | | | | | | | | | | | | | | | | | | | | | |
S14          151 GIIYATQDWVKEKVAADFKGRKILAGNGLVGGGDLSADRSIGLAPSGVTA    200

ATCC27853 201 GSYRSVTVNANGVVTQGSNPTTLAGYAIGDAYTKADTDGKLAQKANKATT    250
             | | | | | | | | | | | | | | | | | | | | | | | | | | | | | | | | | | | | | | | | | | | | | | | | | |
S14          201 GSYRSVTVNANGVVTQGSNPTTLAGYAIGDAYTKADTDGKLAQKANKATT    250

ATCC27853 251 LAGYGITDALRVDGNAVSSSRLAAPRSLAASGDASWSVTFDGSANVSAPL    300
             | | | | | | | | | | | | | | | | | | | | | | | | | | | | | | | | | | | | | | | | | | | | | | | | | |
S14          251 LAGYGITDALRVDGNAVSSSRLAAPRSLAASGDASWSVTFDGSANVSAPL    300

ATCC27853 301 SLSATGVAAGSYPKVTVDTKGRVTAGMALAATDIPGLDASKLVSGVLAEQ    350
             | | | | | | | | | | | | | | | | | | | | | | | | | | | | | | | | | | | | | | | | | | | | | | | | | |
S14          301 SLSATGVAAGSYPKVTVDTKGRVTAGMALAATDIPGLDASKLVSGVLAEQ    350

ATCC27853 351 RLPVFARGLATAVSNSSDPNTATVPLMLTNHANGPVAGRYFYIQSMFYPD    400
             | | | | | | | | | | | | | | | | | | | | | | | | | | | | | | | | | | | | | | | | | | | | | | | | | |
S14          351 RLPVFARGLATAVSNSSDPNTATVPLMLTNHANGPVAGRYFYIQSMFYPD    400

ATCC27853 401 QNGNASQIATSYNATSEMYVRVSYAANPSIREWLPWQRCDIGGSFTKTTD    450
             | | | | | | | | | | | | | | | | | | | | | | | | | | | | | | | | | | | | | | | | | | | | | | | | | |
S14          401 QNGNASQIATSYNATSEMYVRVSYAANPSIREWLPWQRCDIGGSFTKTTD    450

ATCC27853 451 GSIGNGVNINSFVNSGWWLQSTSEWAAGGANYPVGLAGLLIVYRAHADHI    500
             | | | | | | | | | | | | | | | | | | | | | | | | | | | | | | | | | | | | | | | | | | | | | | | | | |
S14          451 GSIGNGVNINSFVNSGWWLQSTSEWAAGGANYPVGLAGLLIVYRAHADHI    500
```

```
ATCC27853 501 YQTYVTLNGSTYSRCCYAGSWRPWRQNWDDGNFDPASYLPKAGFTWAALP    550
              ||||||||||||||||||||||||||||||||||||||||||||||||||
S14       501 YQTYVTLNGSTYSRCCYAGSWRPWRQNWDDGNFDPASYLPKAGFTWAALP    550

ATCC27853 551 GKPATFPPSGHNHDTSQITSGILPLARGGLGANTAAGARNNIGAGVPATA    600
              ||||||||||||||||||||||||||||||||||||||||||||||||||
S14       551 GKPATFPPSGHNHDTSQITSGILPLARGGLGANTAAGARNNIGAGVPATA    600

ATCC27853 601 SRALNGWWKDNDTGLIVQWMQVNVGDHPGGIIDRTLTFPIAFPSACLHVV    650
              ||||||||||||||||||||||||||||||||||||||||||||||||||
S14       601 SRALNGWWKDNDTGLIVQWMQVNVGDHPGGIIDRTLTFPIAFPSACLHVV    650

ATCC27853 651 PTVKEVGRPATSASTVTVADVSVSNTGCVIVSSEYYGLAQNYGIRVMAIG    700
              ||||||||||||||||||||||||||||||||||||||||||||||||||
S14       651 PTVKEVGRPATSASTVTVADVSVSNTGCVIVSSEYYGLAQNYGIRVMAIG    700

ATCC27853 701 Y    701
              |
S14       701 Y    701


#---------------------------------------
#---------------------------------------
```

Supplementary Information 3: Raw data for the absorbance values (OD_{600}) recorded as part of the absorbance-based assays.

Absorbance data for Figure 3A:

Time (h)	*P. aeruginosa* 008			*P. aeruginosa* 008 + pyocin			*P. aeruginosa* 008 + UC (chloroform)			*P. aeruginosa* 008 + UC (freeze-thaw)		
	#1	#2	#3	#1	#2	#3	#1	#2	#3	#1	#2	#3
0	0.19	0.2	0.19	0.18	0.18	0.17	0.17	0.17	0.18	0.17	0.19	0.17
1	0.36	0.37	0.36	0.17	0.18	0.16	0.26	0.27	0.26	0.35	0.37	0.35
2	1.1	1.07	1.06	0.18	0.17	0.15	0.58	0.63	0.61	0.91	0.94	0.9
3	1.76	1.79	1.85	0.16	0.16	0.17	1.36	1.39	1.44	1.65	1.63	1.63
4	2	2	2	0.16	0.16	0.14	1.92	1.92	1.96	2	2	2
5	2	2	2	0.16	0.16	0.14	2	2	2	2	2	2
6	2	2	2	0.16	0.14	0.14	2	2	2	2	2	2

Time (h)	*P. aeruginosa* 008		*P. aeruginosa* 008 + pyocin		*P. aeruginosa* 008 + UC (chloroform)		*P. aeruginosa* 008 + UC (freeze-thaw)	
	**Mean**	**SD**	**Mean**	**SD**	**Mean**	**SD**	**Mean**	**SD**
0	0.193333333	0.005773503	0.176666667	0.005773503	0.173333333	0.005773503	0.176666667	0.011547005
1	0.363333333	0.005773503	0.17	0.01	0.263333333	0.005773503	0.356666667	0.011547005
2	1.076666667	0.02081666	0.166666667	0.015275252	0.606666667	0.025166115	0.916666667	0.02081666
3	1.8	0.045825757	0.163333333	0.005773503	1.396666667	0.040414519	1.636666667	0.011547005
4	2	0	0.153333333	0.011547005	1.933333333	0.023094011	2	0
5	2	0	0.153333333	0.011547005	2	0	2	0
6	2	0	0.146666667	0.011547005	2	0	2	0

Absorbance data for Figure 3B:

Time (h)	*P. aeruginosa* 008			*P. aeruginosa* 008 + Proteinase K treated pyocin			*P. aeruginosa* 008 + Proteinase K		
	#1	#2	#3	#1	#2	#3	#1	#2	#3
0	0.19	0.2	0.19	0.17	0.18	0.19	0.17	0.18	0.19
1	0.36	0.37	0.36	0.16	0.16	0.16	0.36	0.37	0.37
2	1.1	1.07	1.06	0.16	0.17	0.17	1.01	0.98	1
3	1.76	1.79	1.85	0.16	0.15	0.15	1.74	1.76	1.72
4	2	2	2	0.15	0.15	0.14	2	2	2
5	2	2	2	0.16	0.15	0.14	2	2	2
6	2	2	2	0.15	0.15	0.14	2	2	2

Time (h)	*P. aeruginosa* 008		*P. aeruginosa* 008 + Proteinase K treated pyocin		*P. aeruginosa* 008 + Proteinase K	
	**Mean**	**SD**	**Mean**	**SD**	**Mean**	**SD**
0	0.193333	0.005773503	0.18	0.01	0.18	0.01
1	0.363333	0.005773503	0.16	0	0.366666667	0.005773503
2	1.076667	0.02081666	0.166667	0.005773503	0.996666667	0.015275252
3	1.8	0.045825757	0.153333	0.005773503	1.74	0.02
4	2	0	0.146667	0.005773503	2	0
5	2	0	0.15	0.01	2	0
6	2	0	0.146667	0.005773503	2	0

Absorbance data for Figure 3C:

**Time (h)**	***P. aeruginosa* ATCC 27853**			***P. aeruginosa* ATCC 27853 + pyocin**		
	#1	#2	#3	#1	#2	#3
0	0.19	0.19	0.2	0.2	0.2	0.21
1	0.33	0.33	0.34	0.35	0.35	0.36
2	1.22	1.2	1.2	1.16	1.15	1.15
3	1.77	1.76	1.77	1.73	1.72	1.72
4	2	2	2	1.79	1.83	1.83
5	2	2	2	1.92	1.89	1.91
6	2	2	2	1.92	1.91	1.92

**Time (h)**	***P. aeruginosa* ATCC 27853**		***P. aeruginosa* ATCC 27853 + pyocin**	
	**Mean**	**SD**	**Mean**	**SD**
0	0.193333	0.005774	0.203333	0.005774
1	0.333333	0.005774	0.353333	0.005774
2	1.206667	0.011547	1.153333	0.005774
3	1.766667	0.005774	1.723333	0.005774
4	2	0	1.816667	0.023094
5	2	0	1.906667	0.015275
6	2	0	1.916667	0.005774

Absorbance data for Supplementary Figure 4:

**Time (h)**	***P. aeruginosa* 008**			***P. aeruginosa* 008 + pyocin (chloroform treated)**		
	#1	#2	#3	#1	#2	#3
0	0.19	0.2	0.19	0.18	0.19	0.2
1	0.36	0.37	0.36	0.17	0.17	0.18
2	1.1	1.07	1.06	0.17	0.17	0.17
3	1.76	1.79	1.85	0.15	0.16	0.16
4	2	2	2	0.13	0.15	0.14
5	2	2	2	0.13	0.14	0.15
6	2	2	2	0.13	0.14	0.15

**Time (h)**	***P. aeruginosa* 008**		***P. aeruginosa* 008 + pyocin (chloroform treated)**	
	**Mean**	**SD**	**Mean**	**SD**
0	0.193333	0.005773503	0.19	0.01
1	0.363333	0.005773503	0.173333	0.005774
2	1.076667	0.02081666	0.17	0
3	1.8	0.045825757	0.156667	0.005774
4	2	0	0.14	0.01
5	2	0	0.14	0.01
6	2	0	0.14	0.01
